# Physiological functions and pharmacological targeting of transient receptor potential channels

**DOI:** 10.1016/j.pharmr.2025.100089

**Published:** 2025-09-10

**Authors:** Vladimir Chubanov, Christian Grimm, Kerstin Hill, Michael Schaefer, Michael Köttgen, Ursula Storch, Michael Mederos y Schnitzler, Veronika Kudrina, Anna Erbacher, Thomas Gudermann

**Affiliations:** 1 Walther-Straub Institute of Pharmacology and Toxicology, Ludwig Maximilian University of Munich, Munich, Germany; 2 Immunology, Infection and Pandemic Research IIP, Fraunhofer Institute for Translational Medicine and Pharmacology ITMP, Frankfurt, Germany; 3 Department of Pharmacology, University of Oxford, Oxford, United Kingdom; 4 Rudolf-Boehm Institute of Pharmacology and Toxicology, Leipzig University, Leipzig, Germany; 5 Renal Division, Department of Medicine, Medical Center, Faculty of Medicine, University of Freiburg, Freiburg, Germany; 6 Institute of Pharmacy, Clinical Pharmacy, University of Regensburg, Regensburg, Germany; 7 Comprehensive Pneumology Center, a member of the German Center for Lung Research (DZL), Munich, Germany

## Abstract

Transient receptor potential (TRP) channels represent an extensive and diverse protein family fulfilling salient roles as versatile cellular sensors and effectors. The pivotal role of TRP and related ion channels in sensory processes has been well documented. Over the last few years, a new concept has emerged that TRP proteins control an exceptionally broad spectrum of homeostatic physiological functions such as maintenance of body temperature, blood pressure, transmitter release from neurons, mineral and energy homeostasis, and reproduction. This notion is further supported by more than 20 hereditary human diseases in areas as diverse as neurology, cardiology, hematology, pulmonology, nephrology, dermatology, and urology. Most TRP channel-related human disorders impinge on development, metabolism, and other homeostatic functions. The remarkable diversity of pathologies caused by TRP channel dysfunction underscores these proteins' broad spectrum of roles in vivo. Here, we provide a comprehensive overview of our progress in the identification, characterization, and clinical relevance of pharmacological agents targeting mammalian TRP channels.

**Significance Statement:**

Accumulating evidence links transient receptor potential (TRP) channels to various human diseases and highlights TRPs as the most appealing pharmacological targets. The review provides an overview of this quickly developing research area, focusing on identified pharmacological modulators of mammalian TRP channels.

## Graphical abstract

**Figure:**
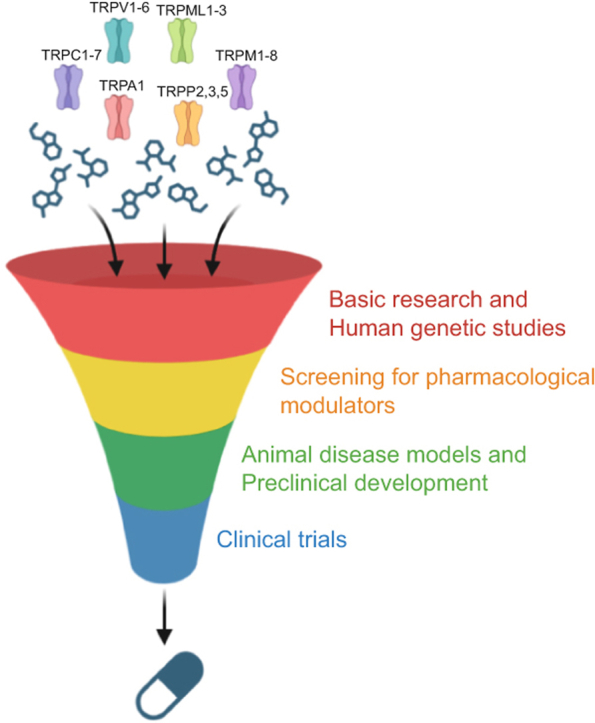

## I Introduction

The transient receptor potential (TRP) gene superfamily represents a large, evolutionarily conserved group of ion channels initially identified in a mutant strain of *Drosophila melanogaster* that displayed an abnormal response to light illumination.^1^ The mutant flies exhibited a rapid decay in the light-induced electroretinogram response of photoreceptor cells, referred to as transient receptor potential, to distinguish it from the sustained receptor potential observed in wild-type (WT) flies.2., 3., 4. Subsequent genetic and molecular investigations unveiled a new type of ion channel, now known as the TRP channel.^5^

A systematic search for homologous proteins led to the discovery of TRP channels across a diverse array of eukaryotes, including algae, fungi, choanoflagellates, invertebrates, and mammals.^6^ In humans, 27 TRP proteins are known, which are subdivided into 6 families (Table 1): canonical TRP channels (TRPCs), vanilloid receptor and related TRP channels (TRPVs), melastatin-like TRP channels (TRPMs), ankyrin domain-enriched TRP channels (TRPAs), mucolipin-related TRP channels (TRPMLs), and polycystic kidney disease-related TRP proteins (TRPPs).^7^^,^^8^

**Table 1:** Nomenclature, structural data, and expression profiles of TRP channels

Gene	Protein and UniProt Enty^a^	Structures in PDB^b^	Expression Pattern
TRPCs			

*TRPC1*	TRPC1 (TRP1); P48995	only as TRPC1/4 heteromer: 8WPL, 8WPM	ubiquitous
*TRPC2*	TRPC2 (TRP2) pseudogene in humans and Old-World monkeys	n.d.	VNO of rodents and other macrosmatic mammals
*TRPC3*	TRPC3 (TRP3); Q13507	7DXB, 7DXC, 7DXD, 7DXE, 6DJR, 6CUD, 5ZBG	brain, heart, lung, blood vessels prostate, placenta, kidney, testis
*TRPC4*	TRPC4 (TRP4, CCE1); Q9UBN4	7B0J, 6G1K, 7B05, 7B0S, 7B16, 6JZO, 5Z96, 8WPN, 7B1G; as TRPC1/4 heteromer: 8WPL, 8WPM	high levels in brain and placenta, lower levels in heart, pancreas, kidney, endothelium
*TRPC5*	TRPC5 (TRP5, CCE2); Q9UL62	7E4T, 7D4P, 7D4Q, 7WDB, 7X6C, 8GVW, 7X6I, 8GVX, 6AEI, 6YSN	high levels in brain, lower levels in kidney, blood vessels, liver, stomach
*TRPC6*	TRPC6 (TRP6); Q9Y210	7DXF, 7DXG, 6UZB, 6UZA, 5YX9, 7A6U	placenta, lung, blood vessels, spleen, ovary, small intestine, neutrophils, podocytes
*TRPC7*	TRPC7 (TRP7); Q9HCX4	n.d.	hypophysis, kidney, heart, lung, blood vessel, eye, spleen, testis

TRPVs			

*TRPV1*	TRPV1 (VR1, OTRPC1); Q8NER1	5IRX, 5IRZ, 5IS0, 7L2H, 7L2I, 7L2J, 7L2K, 7L2L, 7L2M, 7L2N, 7L2O, 7L2P, 7L2R, 7L2S, 7L2T, 7L2U, 7L2V, 7L2W, 7L2X, 7LP9, 7LPA, 7LPB, 7LPC, 7LPD, 7LPE, 7LQY, 7LQZ, 7LR0, 7MZ5, 7MZ6, 7MZ7, 7MZ9, 7MZA, 7MZB, 7MZC, 7MZD, 7MZE, 7RQU, 7RQV, 7RQW, 7RQX, 7RQY, 7RQZ, 8GF8, 8GF9, 8GFA, 8JQR, 8T0C, 8T0E, 8T0Y, 8T10, 8T3L, 8T3M, 8U2Z, 8U30, 8U3A, 8U3C, 8U3J, 8U3L, 8U43, 8U4D, 8X94	small-to medium diameter DRG and trigeminal ganglion sensory neurons, brain neurons, astrocytes and microglia
*TRPV2*	TRPV2 (VRL-1, OTRPC2); Q9Y5S1	6OO3, 6OO4, 6OO5, 6OO7, 7XEM, 7XEO, 7XER, 7XEU, 7XEV, 7XEW, 7YEP, 6BWJ, 6BWM, 5AN8, 8SLX, 8SLY, 8FFL, 8FFM, 8FFN, 8FFQ, 5HI9, 6BO4, 6BO5, 6U84, 6U86, 6U88, 6U8A, 7N0M, 7N0N, 7T37, 7T38, 7ZJD, 7ZJE, 7ZJG, 7ZJH, 7ZJI, 9B3U, 9B3V, 9B3W, 9B3X, 9B3Y, 9B3Z, 8EKP, 8EKQ, 8EKR, 8EKS	medium-to-large diameter DRG and trigeminal ganglion sensory neurons, various immune cell types, red blood cells, neurons, microglial cells, melanocytes, vascular smooth muscle cells, urothelium
*TRPV3*	TRPV3 (VRL3, oTRPC3); Q8NET8	*Tetrameric* 6DVW, 6DVY, 6DVZ, 6MHO, 6MHS, 6MHV, 6MHW, 6MHX, 6OT2, 6OT5, 6PVL, 6PVM, 6PVN, 6PVO, 6PVP, 6PVQ, 6LGP, 6UW4, 6UW6, 6UW8, 6UW9, 7MIJ, 7MIK, 7MIL, 7MIM, 7MIN, 7MIO, 7RAS, 7RAU, 7UGG, 7XJ0, 7XJ1, 7XJ2, 7XJ3, 8GKA, 8V6K, 8V6L, 8V6M, 8V6N, 8V6O, 9JDM, 9JE5, 9JEE, 9JEF, 9JEG, 9BKU	keratinocytes, oral gingival and epithelial cells, glandular cells and enterocytes in the small and large intestine
		*Pentameric* 8GKG, 9DIJ	
*TRPV4*	TRPV4 (TRP12, VRL-2, oTRPC4); Q9HBA0	8T1B, 8T1C, 8T1D, 8T1E, 8T1F, 8FC7, 8FC8, 8FC9, 8FCA, 8FCB, 8J1B, 8J1D, 8J1F, 8J1H, 8JKM, 8JU5, 8JU6, 8JVI, 8JVJ	Ubiquitous in vascular endothelial cells, pancreatic, tongue and salivary gland exocrine epithelial cells, epithelial cells in kidney tubules, bronchial, tracheal and fallopian tube ciliated cells, skin keratinocytes and melanocytes, macrophages, hepatic Kupffer cells, placentar trophoblast, and decidual cells
*TRPV5*	TRPV5 (ECaC, ECaC1, CAT2, OTRPC3); Q9NQA5	6B5V, 6DMR, 6DMU, 6DMW, 6O1N, 6O1P, 6O1U, 6O20, 6PBE, 6PBF, 7T6J, 7T6K, 7T6L, 7T6M, 7T6N, 7T6O, 7T6P, 7T6Q, 7T6R, 8FFO, 8FHH, 8FHI, 8TF2, 8TF3, 8TF4, 8FFL, 8FFM, 8FFN, 8FFQ	DCT and collecting duct of the kidney, pancreas, small and large intestine, prostate gland, testis, brain, bone osteoclasts, and placenta
*TRPV6*	TRPV6 (CaT1, ECaC2, OTRPC3); Q9H1D0	5IWK, 5IWP, 5IWR, 5IWT, 5WO6, 5WO7, 5WO8, 5WO9, 5WOA, 6BO8, 6BO9, 6BOA, 6BOB, 6D7O, 6D7P, 6D7Q, 6D7S, 6D7T, 6D7V, 6D7X, 6E2F, 6E2G, 7D2K, 7K4A, 7K4B, 7K4C, 7K4D, 7K4E, 7K4F, 7S88, 7S89, 7S8B, 7S8C, 8FOA, 8FOB, 8SP8, 9CUH, 9CUI, 9CUJ, 9CUK	Small intestine, glandular cells of the salivary gland, pancreas, prostate, thyroid, bronchiae, placenta, testis, epididymis, endometrium, stomach, caecum, main olfactory epithelium

TRPMs			

*TRPM1*	TRPM1 (Melastatin, MLSN1, LTRPC1); Q7Z4N2	n.d.	melanocytes, retinal ON bipolar cells
*TRPM2*	TRPM2 (LTRPC2); O94759	6MIX, 6MIZ, 6MJ2, 6PUO, 6PUR, 6PUU, 6PUS, 7VQ1, 8E6Q, 8E6T, 8E6R, 8E6S, 8E6U	ubiquitous; high levels in brain and immune cells
*TRPM3*	TRPM3 (MLSN2, LTRPC3, TRPM3α2); Q9HCF6	8ED7, 8ED8, 8ED9, 8DDR, 8DDS, 8DDT, 8DDX, 8DDQ, 8DDU, 8DDV, 8DDW, 9B2A, 9B29, 9B28	DRG sensory neurons, brain, kidney, pancreatic *β*-cells, placenta, testis
*TRPM4*	TRPM4 (LTRPC4, TRPM4B); Q8TD43	9B93, 6BQV, 9B90, 6BCO, 6BCQ, 9B92, 9B94, 6BCL, 5WP6, 6BWI, 8RCR, 8RCU, 8RD9, 9B8W, 9B8Y, 6BCJ, 6BQR	ubiquitous; high levels in brain, heart, immune cells, and pancreatic *β*-cells
*TRPM5*	TRPM5 (MTR1, LTRPC5); Q9NZQ8	8SLE, 8SL6, 8SL8, 8SLA, 8SLI, 8SLP, 8SLQ, 8SLW	type II taste receptor cells, tuft cells, olfactory epithelium, and pancreatic *β*-cells
*TRPM6*	TRPM6 (ChaK2, Channel-kinase 2); Q9BX84	n.d.	kidney, intestine, placenta, lung, testis
*TRPM7*	TRPM7 (LTRPC7, TRP-PLIK, ChaK1, Channel-kinase 1, MagNum, MIC); Q96QT4	5ZX5, 6BWF, 6BWD, 8SI2, 8SI3, 8SIA, 8SI7, 8SI5, 8SI6, 8SI4, 8SI8, 8W2L; kinase domain: 1IAH, 1IA9, 1IAJ	ubiquitous
*TRPM8*	TRPM8 (Trp-p8, CMR1, Cold receptor 1); Q7Z2W7	8BDC, 8E4L, 8E4M, 8E4N, 8E4O, 8E4P, 9B6D, 9B6E, 9B6F, 9B6G, 9B6H, 9B6J, 9B6K, 7WRA, 7RWB, 7WRC, 7WRD, 7WRE, 7WRF	DRG and TG sensory neurons, brain, prostate, pancreatic *β*-cells, placenta, testis

TRPAs			

*TRPA1*	TRPA1 (ANKTM1, TRPN1); O75762	3J9P, 6PQO, 6PQP, 6PQQ, 6V9V, 6V9W, 6V9X, 6V9Y, 6X2J, 6WJ5, 7JUP, 7OR0, 7OR1, 9MOE	DRG, trigeminal and vagal ganglia, enterochromaffin cells, astrocytes, Schwann cells, bronchial, alveolar, renal and urothelial epithelial cells, keratinocytes, melanocytes, cardiac fibroblasts, pancreatic *β*-cells, enterochromaffin cells, T-cells, pancreatic and colon cancer, neuroblastoma, glioblastoma

TRPMLs			

*TRPML1*	TRPML1 *(*MCOLN1, Mucolipin1); Q9GZU1	7SQ7, 7SQ8, 7SQ9, 5WPQ, 5WPT, 5WPV, 7MGL, 9CBZ, 9CBZ, 9CBZ, 5YDZ, 5YE2, 9CC2, 5YE5, 9EKT, 7SQ6, 9EKS, 9EKU, 6E7P, 6E7Y, 6E7Z, 5YE1, 9EKV, 5WJ5, 5WJ9, 9HJ6, 9HJ8, 9HL3, 9HlL4, 9HL6, 9HL8, 9HLA, 9HLB, 9HLC, 9HLD	ubiquitous
*TRPML2*	TRPML2 *(*MCOLN2, Mucolipin2); Q8IZK6	7DYS, 9EKW, 9EKX, 9EKY, 9EKZ, 9EKO, 8EL1, 6HRS, 6HRR	thymus, spleen, kidney, trachea, liver, lung, colon, testis, thyroid, B- and T-cells, macrophages, dendritic cells
*TRPML3*	TRPML3 (MCOLN3, Mucolipin3); Q8TDD5	6AYG, 6AYE, 6AYF	hair cells of the inner ear, organ of corti, utricle, stria vascularis, alveolar macrophages, skin melanocytes, neonatal enterocytes, kidney, lung, olfactory bulb (sensory neurons), nasal cavity, thymus, colon, trachea, several glands (parathyroid, salivary, adrenal, pituitary), testis, ovary

TRPP channels			

*PKD2*	TRPP2 (polycystin-2, PC2, PKD2); Q13563	8HK7, 8K3S, 6D1W, 9DLI, 9DWQ, 5K47, 5MKE, 5MKF, 5T4D, 6T9N, 6T9O, 6WB8; as PKD1-PKD2 heteromer: 6A70	ubiquitous; high levels in the kidney, brain, heart
*PKD2L1*	TRPP3 (polycystin L, PKD2L1); Q9P0L9	5Z1W, 6DU8; as PKD1L3-CTD-PKD2L1 heteromer: 7D7E, 7D7F	brain, taste receptor cells, kidney, lung
*PKD2L2*	TRPP5 (PKD2L2); Q9NZM6	n.d.	testis and brain, low levels in the kidney, liver, heart, lung

n.d., not determined.

a accession numbers for human proteins in UniProt.

b experimentally addressed structures for human/rodent/fish (TRPC), human/rodent (TRPV, TRPM, TRPML, TRPP), or human (TRPA) proteins in RCSB PDB (Research Collaboratory for Structural Bioinformatics Protein Data Bank).

**Fig. 1:**
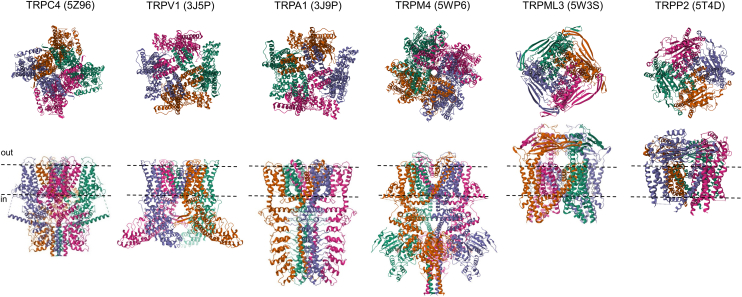
Representative structures of TRP channels. Cryo-EM structures of TRPC4, TRPV1, TRPA1, TRPM4, TRPML3, and TRPP2 channels are shown from extracellularly (top) and parallel to the plasma membrane (bottom), with the exception that TRPML3 is viewed from the extracytosolic side (top) and the lysosomal membrane plane (bottom). The 4 subunits are colored in blue, pink, green, and brown to outline the tetrameric assembly of the channels. TRPML3 and TRRP2 contain a cap-like structure above a channel pore entrance.

All TRP channels maintain a notable structural similarity in their channel-pore forming domains, which include 6 membrane-spanning helices and a short stretch of hydrophobic residues between the fifth and sixth transmembrane (TM) segments, often called the pore helix (PH). After the sixth helix, TRPCs, TRPVs, and TRPMs also feature a highly conserved segment known as the TRP domain. TRPPs and TRPMLs exhibit a more distinct topology of the channel segment because they include a long loop linking the first 2 TM helices and lack the TRP domain. Besides this, TRP channels display significant structural heterogeneity in their large N- and C-terminal domains. TRP proteins are assembled in tetramers, implying that 4 subunits contribute to a common membrane-spanning channel pore (Fig. 1).

Over the past decade, significant progress has been made in single-particle cryogenic electron microscopy (cryo-EM) analysis of TRP channels, resulting in more than 400 structural models that encompass nearly all vertebrate TRP proteins (Table 1). The structural data obtained enable a detailed examination of the 3-dimensional (3D) arrangement of channel subunits at the atomic level and elucidate the roles of certain amino acids in the tetrameric assembly of TRP channels (Fig. 1). Undoubtedly, these results provide a foundation for structure-function analysis of TRP channels, including insights into regulatory mechanisms, the functional effects of pathogenic mutations, and structure-based drug design.

Apart from considerable structural variability (Fig. 1), TRP channels also display fascinating diversity in functional characteristics, subcellular distribution, expression patterns, and physiological roles (Table 1).^9^^,^^10^ The pivotal role of TRP and related ion channels in sensory processes has been highlighted by the 2021 Nobel Prize in Physiology or Medicine, awarded to David Julius and Ardem Patapoutian.^11^ Clinical studies and experiments on preclinical disease models revealed the prominent role of TRP proteins in human health and disease.^12^ Accordingly, TRP proteins have been identified as the most appealing pharmacological targets.^13^^,^^14^ This review provides an up-to-date assessment of TRP channels, emphasizing our progress in developing pharmacological agents that allow selective modulation of mammalian TRP channels in diverse pathophysiological settings.

## II TRPCs

### A TRPC gene family

**Fig. 2:**
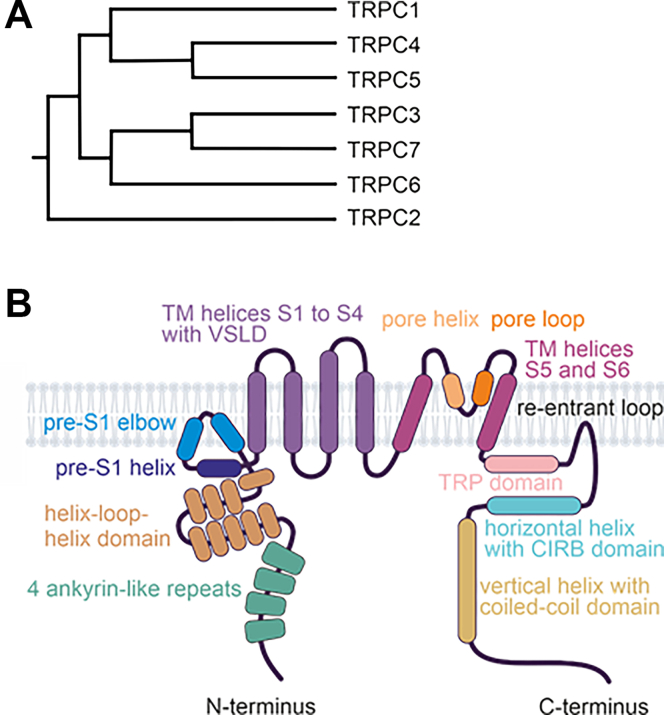
The relatedness and domain topology of TRPCs. (A) Phylogenetic tree of the human TRPC1–7 proteins. Since TRPC2 is a pseudogene in humans, mouse TRPC2 is displayed. (B) Schematic representation of a TRPC monomer using the example of TRPC6.

The TRPC gene family in mammals consists of 7 members (Table 1): TRPC1–7. Notably, TRPC2 is a pseudogene in humans, as well as in Old World monkeys and apes (Catarrhini).^15^^,^^16^ Based on amino acid sequence similarity, the TRPC family is divided into 4 subgroups: TRPC1, TRPC2, TRPC3/6/7, and TRPC4/5 (Fig. 2A).

Dysregulation of specific TRPCs has been implicated in various disease states, including pulmonary and renal diseases, as well as neurological disorders. Despite recent advances in the development of potent and selective TRPC modulators, substantial clinical benefits have yet to be realized. This underscores the need for further research to fully elucidate the role of TRPCs in health and disease.

### B Domain topology, assembly, and functional characteristics of individual TRPCs

#### 1 Domain topology of TRPCs

In 2018, the first high-resolution 3D structures of TRPCs were resolved using single-particle cryo-EM technology. Since then, several 3D structures of TRPC3, TRPC4, TRPC5, and TRPC6 channels, and TRPC1/4 heteromers have been identified, with resolutions ranging from 2.4 Å to 4.4 Å. These structures were obtained in the presence and absence of inhibitors, activators, and Ca^2+^, or in complex with interacting proteins such as calmodulin (CaM) or G*α*i protein subunits.17., 18., 19., 20., 21., 22., 23., 24., 25., 26., 27., 28., 29., 30. Table 1 summarizes the available Protein Data Bank (PDB) entries. Studies have revealed that all TRPCs exhibit a tetrameric structure, with each channel subunit adopting a Y-shaped arrangement, giving the tetramers rotational symmetry. The 4 monomers collectively form the central channel pore, which is permeable to both monovalent and divalent cations. Each channel monomer comprises 6 TM domains (TMDs) (S1–S6), formed by *α*-helices, with cytosolic N- and C-termini. The general structural features of TRPCs are depicted in Fig. 2B.

The N-terminus of TRPCs contains 4 ankyrin-like repeats, conserved across other TRP channel families such as TRPMs, TRPVs, and TRPA1.^31^ These repeats play critical roles in protein or cytoskeleton interactions^32^^,^^33^ and stabilize the distal cytoplasmic portions of the C-terminus.^29^^,^^34^^,^^35^ The first ankyrin-like repeat appears crucial for channel homo- or heterotetramerization,^36^ as it interacts with a connecting helix – the rib helix – and the C-terminus of the adjacent monomer, potentially stabilizing the tetramer.^20^ Notably, the 3D structure of TRPC5 in complex with the G*α*i3 protein subunit has revealed direct interaction between G*α*i3 and the ankyrin-like repeat domains 1 and 2.^27^

TRPC4 and TRPC5 channels feature 7 helices, while TRPC3 and TRPC6 contain 9 helices of varying lengths, connected by loops (helix-loop-helix domain) located at the proximal N-terminus. This domain interacts with the C-terminal TRP domain, a conserved structure that follows the S6 TMD. Adjacent to this domain lies the pre-S1 elbow, a membrane-associated structure consisting of 2 helices. The following pre-S1 helix itself is parallel to the cytoplasmic membrane surface.

The TM helices S1–S4 form a Y-shaped “shank” and a voltage-sensing-like domain (VSLD) that harbors binding sites for various channel modulators. The second Y-shaped "leg" is composed of the TM helices S5 and S6, connected via the PH and pore loop. The ion-conducting pathway is primarily formed by S5, S6, and the pore domain, which includes the PH, turret, and loop. The pore walls are constructed of 4 pore loops and their corresponding S6 helices, with the extracellular pore region carrying a negative charge. TRPC5 has one additional negatively charged amino acid compared with TRPC4,^23^ potentially explaining the higher single-channel conductance of TRPC5.^37^ Mutations in this extracellular pore region alter channel properties, underscoring its role in ion selectivity and conductivity.^23^^,^^38^

The leucine-phenylalanine-tryptophan (LFW) motif in the PH, along with upstream residues, stabilizes the pore domain by interacting with the S5 domain of the same monomer and the S6 domain of an adjacent monomer. The ion selectivity filter is formed by phenylalanine and glycine residues, which are located 2 amino acids downstream of the LFW motif at the end of the PH and at the beginning of the pore loop, with their carbonyl oxygens interacting electrostatically with permeating cations, thereby forming the ion selectivity filter.^28^ Below the selectivity filter lies a central, presumably water-filled, pore cavity formed by the S6 helix, with constriction sites at the cytoplasmic end. The narrowest part of the ion-conducting pore, the lower gate, is usually formed by 3 amino acids. These constriction sites, spaced by 3 amino acids each, consist of valine, histidine, and glutamine in TRPC1^30^; leucine and isoleucine^17^ or leucine, isoleucine, and phenylalanine^19^ in TRPC3; isoleucine, asparagine, and glutamine in TRPC4^22^ and TRPC5^23^; and leucine, isoleucine, and phenylalanine^28^ or the adjacent amino acids isoleucine, asparagine, and glutamine^18^ in TRPC6. Interestingly, it was suggested that, depending on the selected inhibitor, the lower gate of TRPC6 is formed by isoleucine, asparagine, and glutamine.^19^

Another conserved structure in TRPCs is the helical TRP domain, located proximally to the intracellular C-terminus. It includes the TRP box, which contains the amino acid motif glutamate-tryptophan-lysine-phenylalanine-alanine-arginine (EWKFAR). This motif stabilizes the cytoplasmic proximal section of the channel. The tryptophan and phenylalanine residues of the TRP box, along with tyrosine and lysine residues of the TRP helix, interact with the N-terminal proximal helices (helix-loop-helix domain), the intracellular loop between S4 and S5, and the cytoplasmic ends of the S1 and S4 helices. Consequently, the TRP domain forms a stabilizing contact surface between the TM regions and the proximal cytoplasmic sections of the channel.

Although the precise function of the TRP box remains unclear, growing evidence suggests that it plays a critical role in channel-gating regulation. For instance, glycine at position 503 in TRPC4 affects gating, as a substitution for serine results in uncontrolled channel opening.^39^ Glycine 503 interacts with tryptophan 635 in the TRP domain, stabilizing the S6 segment that constitutes the lower gate.^20^ Additionally, the TRP domain may interact with phosphoinositol-4,5-bisphosphate (PIP_2_), as observed in other TRP channels like TRPM8.40., 41., 42. In TRPC6, PIP_2_ binding likely occurs between the distal TRP box and the pre-S1 helix.^43^ A lysine-to-alanine substitution at position 771 in the TRP box potentiates TRPC6 currents, altering the channel state. In TRPC3, PIP_2_ binds at the intersection of the pre-S1 helix and the S1 TM helix, inducing conformational changes via the re-entrant loop and TRP domain.^44^ Altogether, the TRP domain is highly conserved within the TRP channel superfamily and is crucial for structural stabilization, gating regulation, and cofactor interactions.

Following the TRP domain, a loop containing 2 glutamates (in TRPC4 and TRPC6), 2 aspartates (in TRPC1 and TRPC3), or an aspartate-glutamate combination (in TRPC5 and TRPC7) extends into the cytoplasmic membrane layer. This loop interacts with the pre-S1 elbow of the N-terminus and the cytoplasmic end of the S1 helix. Although not resolved in all 3D structures, it is presumed to be a common feature of TRPCs.

TRPC4 and TRPC5 possess a unique extracellular disulfide bond between 2 cysteines near the S5 PH linker, which may play a role in redox sensing.^22^^,^^45^ Additionally, TRPC3 and TRPC6 differ from TRPC4 and TRPC5 in the length of their S3 helices on the extracellular side, which are approximately 4 helical turns longer.

A nearly parallel helix, slightly sloping toward the cytoplasmic membrane, has been variously termed “CH1”,^18^ “horizontal helix”,^29^ or “CTD rib helix”.^17^ In TRPC4 and TRPC5, it is referred to as the “connecting helix”^22^^,^^23^ or “Rib helix + CaM 1,4,5-trisphosphate (IP_3_) receptor binding (CIRB) domain”.^20^ The CIRB domain, present in all TRPCs, begins before the horizontal helix and encompasses most of it. The 3D structure of TRPC4 in complex with CaM reveals an interaction between 1 channel monomer and 1 CaM protein at the CIRB domain.^21^ Interestingly, CaM binding depends on calcium concentration: at low calcium levels, IP_3_ receptor binding is favored, while at high calcium levels, CaM preferentially binds. CaM stabilizes the inactive channel state, whereas IP_3_ receptor binding promotes activation.^21^^,^^46^^,^^47^

At the distal end of the C-terminus, a perpendicular helix forms another conserved feature, variously called “CH2”,^18^ “CTD pole helix”,^17^ “vertical helix”,^29^ “coiled-coil (CC) domain”,^22^^,^^23^ or “C-term helix”^20^ in TRPC4 and TRPC5. This helix exhibits a heptahedron-like pattern, and 4 such helices assemble to form a central, vertically extending tunnel in the distal cytoplasmic region. Together with the ankyrin repeat domains (ARDs), this structure is critical for tetrameric assembly.

TRPCs can be regulated by lipids, though the precise mechanism of lipid regulation remains incompletely understood. Recently, 2 distinct lipid-binding sites have been identified. Lipid binding site 1 is located in the inner leaflet of the VSLD, while lipid binding site 2 is situated in the pore region between the pore loop and the S6 helix of an adjacent channel monomer. In the apo states of TRPC523., 24., 25. and TRPC4,^20^^,^^22^ a lipid has been found in the pore region, interacting with the phenylalanine and tryptophan residues of the LFW motif. This lipid has been identified as ceramide-1-phosphate, phosphatidic acid,^23^^,^^25^ or diacylglycerol (DAG).^24^^,^^48^ Mutations in lipid binding site 2 have been shown to affect the DAG sensitivity of TRPC3 and TRPC6 channels,^28^^,^^49^^,^^50^ highlighting this region’s critical role in regulating channel activity.

In the 3D structure of TRPC6, a lipid identified as phosphatidylcholine was found in lipid binding site 2, interacting with the phenylalanine and tryptophan residues of the LFW motif.^28^ Interestingly, this lipid was shifted upward toward the extracellular side and rotated vertically compared with the lipids found in the TRPC4 and TRPC5 structures.

At lipid binding site 1, cholesterol hemisuccinate—introduced during protein purification—was detected. However, in the TRPC3 structure, a phospholipid was identified at this site,^17^ which may represent an endogenous lipid that binds to the channel. Additionally, the 3D structures of TRPC4 in both their ligand-bound and apo states^21^ suggest the presence of a Ca^2+^ ion in the VSLD domain. This ion appears to stabilize ligand binding in the VSLD through a bridging water molecule. Similar cation densities have been observed in the TRPC4 and TRPC5 structures.^22^^,^^23^ Deleting the Ca^2+^ binding site in TRPC5 abolished channel activation by elevated extracellular Ca^2+^ concentrations.^24^

To date, high-resolution structures of TRPCs in their open state are still lacking, and significant portions of the C-terminal region remain unresolved. However, comparisons of TRPC6 3D structures in the apo and activator-bound states suggest that channel opening involves critical movements of the TM helices.^28^ Upon activation, the S6 helix of TRPC6 relaxes and moves downward, widening the restriction point at the lower gate, which is formed by leucine, isoleucine, and phenylalanine residues. This movement is accompanied by a downward bending of the S5 helix. Conversely, Vinayagam et al^21^ propose a different mechanism, in which channel activation involves movements of the VSLD and the TRP box.

#### 2 Assembly of TRPCs

TRPCs can assemble into either homotetrameric or heterotetrameric channel complexes. However, TRPC1 alone may not form functional homotetrameric channels, at least in overexpression systems. Instead, it can form heterotetrameric channel complexes with other TRPC subunits^30^^,^51., 52., 53., 54., 55., 56. and even with subunits from other TRP channel families, such as TRPP2,^57^ TRPV4,^58^ and TRPV6.^59^

In neurons, TRPC1 can heterotetramerize with TRPC4 and TRPC5 to form TRPC1/4/5 channels.^54^^,^^55^^,^60., 61., 62. Within these heterotetramers, TRPC1 alters the permeation properties and reduces calcium permeability.^51^^,^63., 64., 65. Additionally, TRPC1 plays a regulatory role as part of a multiprotein complex that includes stromal interaction molecule 1 (STIM1) and the calcium release-activated calcium channel protein Orai, both of which are involved in store-operated calcium entry (SOCE).66., 67., 68. Heterotetrameric complexes can also form between TRPC3, TRPC6, and TRPC7 subunits,^60^^,^^69^^,^^70^ as well as between TRPC3 and TRPC4.^71^ TRPC2 channels, which are highly expressed in the vomeronasal organs (VNOs) of rodents^72^ but are pseudogenes in humans, are more likely to exist as homomeric channels. In the brain, TRPC3, TRPC6, and TRPC7 preferentially form homomeric channels.^62^ Assumably, the expression of TRPCs as homomeric or heteromeric channels might vary between different cells and tissues.

#### 3 Functional characteristics of individual TRPCs

TRPCs are widely recognized as nonselective, calcium-permeable, and receptor-operated cation channels. These channels are activated downstream of phospholipase C (PLC) following the activation of G_q/11_ protein-coupled receptors or receptor tyrosine kinases.^5^^,^^73^ PLC activation leads to the cleavage of PIP_2_ into the second messengers, inositol IP_3_ and DAG. IP_3_ promotes calcium release from intracellular stores, increasing the free intracellular calcium concentration, while DAG directly activates TRPCs, facilitating sodium and calcium influx and triggering cellular effects. All TRPCs can be directly activated by DAG,74., 75., 76., 77., 78. suggesting that DAG serves as an endogenous activator. However, the DAG sensitivity of TRPC4 and TRPC5 channels is tightly regulated and requires dephosphorylation of a threonine residue in the C-terminal postsynaptic density protein, *Drosophila* disc large tumor suppressor, and zonula occludens-1 protein (PDZ)-binding motif. This motif is unique to TRPC4 and TRPC5 channels and allows for the replacement of Na^+^/H^+^ exchanger regulatory factor (NHERF) 1 and 2 adapter proteins, which are essential for DAG sensitivity.77., 78., 79.

In the case of TRPC3, the use of the photoswitchable DAG derivative, OptoDArG, revealed that DAG might activate the channel through a fenestration involving a conserved glycine residue behind the channel's selectivity filter.^49^ However, the precise mechanism underlying lipid sensing remains to be fully elucidated. IP_3_ and IP_3_ receptors also modulate TRPC function. IP_3_ can directly activate TRPC7 channels,^80^ while IP_3_ receptors activate TRPC3 and TRPC5 channels.^47^^,^^81^ This interaction occurs via the C-terminal CIRB motif of TRPCs, where IP_3_ receptor binding competes with CaM binding.^21^^,^^46^^,^^47^^,^^82^^,^^83^ The binding of IP_3_ receptors establishes an active channel state, while CaM binding promotes an inactive state.

TRPC activity is further influenced by junctate, a TM protein expressed in the endoplasmic reticulum (ER) membrane that interacts with IP_3_ receptors.84., 85., 86. Junctate serves as a calcium-sensing structural component of Orai and STIM1 within the ER membrane at ER-plasma membrane junctions.^87^ Notably, junctate enhances the formation of ER-plasma membrane junctions containing TRPC3 and IP_3_ receptors,^86^ which may represent a mechanism by which IP_3_ receptors and TRPCs contribute to SOCE.

A store-operated activation mechanism has been proposed for TRPCs based on observations that the depletion of intracellular calcium stores triggers calcium influx through the plasma membrane.^88^ This phenomenon is associated with the highly Ca^2+^-selective calcium release-activated current.^89^ However, this current does not share the nonselective characteristics of TRPC currents. Despite this, TRPC1 has been suggested to function as a store-operated channel, either alone or in complex with Orai.90., 91., 92., 93. Additionally, TRPC1 may also be activated via a receptor-dependent mechanism that involves store depletion, effectively integrating both activation pathways.^94^

Currently, it is widely accepted that Orai and STIM proteins are the primary molecular components of store-operated calcium influx.95., 96., 97., 98., 99. STIM serves as a calcium sensor in the ER membrane and activates Orai proteins, which form the channel pore. Evidence suggests that TRPCs, particularly TRPC1, may interact with STIM and/or Orai, modulating SOCE.^100^^,^^101^ Nevertheless, while Orai is essential for calcium influx following store depletion, TRPCs are not strictly required, as demonstrated in studies using mice lacking all 7 TRPC genes.^102^

The membrane lipid PIP_2_ also modulates TRPC function. PIP_2_, as a substrate of PLC, plays a role in receptor-operated signaling pathways but can also act as a second messenger that regulates cellular processes, potentially influencing ion channel activity.^103^^,^^104^ Interestingly, the effects of PIP_2_ vary depending on the patch-clamp configuration used. In inside-out patches, heterologously expressed TRPC5, TRPC3, TRPC6, and TRPC7 channels are activated by PIP_2_,^105^^,^^106^ whereas endogenously expressed TRPC6 channels are inhibited.107., 108., 109. Whole-cell patch-clamp recordings of heterologously overexpressed TRPC5 channels show that PIP_2_ depletion activates TRPC5,^77^^,^^106^ while PIP_2_ application through the patch pipette inhibits the channel. TRPC4 channels are similarly inhibited by PIP_2_ in an isoform-specific manner, with PIP_2_ binding to the C-terminal region stabilizing the inactive channel state.^110^ Furthermore, the intracellular application of PIP_2_ reduces TRPC5 desensitization following receptor activation.^111^

In overexpression systems, PIP_2_ depletion induces a conformational change in the TRPC5 C-terminal region, causing the dissociation of NHERF and conferring direct sensitivity to DAG.^77^ This NHERF dissociation also occurs following protein kinase C (PKC) inhibition or threonine mutation in the C-terminal PDZ-binding motif of TRPC4/5 channels.^77^ Similar PKC-related modulation of DAG sensitivity has been reported,^78^^,^^79^ suggesting a regulatory role of PIP_2_ in TRPC function. Notably, PIP_2_ application through the patch pipette enhances DAG-induced TRPC5 currents after PKC inhibition.^78^

A PIP_2_-binding site has been proposed for TRPC5 near the linker regions between the S2 and S3 helices, the S4 and S5 helices, the TRP helix, and the helix-loop-helix domain.^27^ The intracellular application of PIP_2_ increases the open probability of TRPC5 channels.^27^ Moreover, trivalent cations and DAG allosterically modulate PIP_2_ binding to TRPC5, underscoring PIP_2_ as a critical factor in channel activation and inactivation.^78^ Additionally, PIP_2_ binding to TRPC5 is enhanced by the interaction with G*α*i protein subunits, making TRPC5 more readily open in the cell membrane.^27^

Lipid regulation of TRPC3 channels may require an interplay between PIP_2_ and DAG.^112^ Cleavage of PIP_2_ by PLC generates DAG, which can bind to lipid-binding site 2 within the channel pore, while PIP_2_ interacts with lipid-binding site 1 near the VSLD. This interaction inhibits TRPC3 channel opening, regulating DAG's access to lipid-binding site 2.^112^ Furthermore, it has been proposed that PIP_2_ modulates the ionic selectivity of the TRPC3 pore following receptor stimulation, and that in PIP_2_-rich membrane domains, TRPC3 may be recruited to ER-plasma membrane junctions, suggesting an interaction between TRPC3 and STIM1 to regulate calcium influx.^112^

In TRPC6 channels, amino acid substitutions in the PIP_2_-binding site at the pre-S1 helix^43^ reduce receptor- and 1-oleoyl-2-acetyl-sn-glycerol–induced TRPC6 currents, indicating that PIP_2_ binding enhances channel activity. However, substituting lysine with glutamine in the distal TRP box reverses this effect, potentiating TRPC6 currents at low PIP_2_ concentrations.^43^ This highlights the critical role of the C-terminus in PIP_2_-mediated regulation of TRPCs. Despite these findings, the lipid regulation of TRPCs, including the precise roles of PIP_2_ and DAG in channel activation, remains incompletely understood.

Cleavage of PIP_2_ by PLC also produces protons at the cytoplasmic side of the plasma membrane, causing localized acidification.^113^ This acidification may influence TRPC4 channel activity by sensitizing PLC*δ*1 to calcium, leading to its activation and the potentiation of TRPC4 currents.^114^ The signaling pathway for TRPC activation is complex, involving multiple components whose interplay is not yet fully elucidated. Additionally, extracellular protons^115^ and trivalent cations, such as lanthanum and gadolinium,^38^^,^^116^ can potentiate TRPC4 and TRPC5 currents. Interestingly, TRPC4 and TRPC5 channels can be activated not only downstream of G_q/11_ protein but also via G_i/o_ protein-coupled receptor activation. For example, G_i/o_ protein-coupled receptor activation triggers TRPC4 channel opening through PLC*δ*1 activation.^114^ However, TRPC4 activation by G_i/o_ protein-coupled receptors may also occur independently of PLC, relying instead on a direct interaction with G*α*i proteins.^117^ Similarly, TRPC5 channels are activated by G*α*i proteins.^118^

Recent structural analyses^27^ have revealed that G*α*i proteins directly interact with TRPC5 channels via the N-terminal ankyrin-like repeat domains 1 and 2, leading to channel activation in the presence of PIP_2_. Direct interactions between G proteins and ion channels have so far been well established only for G protein-activated inwardly rectifying potassium channels, where G_*βγ*_ subunits directly bind to the channel.^119^ It has also been proposed that TRPC5 channels are activated downstream of G_s_ protein-coupled receptor stimulation through a cAMP-mediated intracellular calcium release.^120^

The free intracellular calcium concentration also plays a critical role in regulating TRPC activity. Increasing free intracellular calcium levels above 300 nM activates TRPC4 and TRPC5 channels.^37^^,^^121^ Consequently, calcium release from the ER following receptor activation can induce TRPC4 and TRPC5 channel opening, further elevating free intracellular calcium levels and enhancing sodium and calcium influx. Simultaneously, depletion of intracellular calcium stores activates Orai channels, contributing to an additional increase in free intracellular calcium. Free intracellular calcium concentrations of approximately 1 *μ*M were shown to potentiate receptor-operated TRPC5 channels.^122^ Even higher concentrations, with an EC_50_ of around 12 *μ*M, are required to activate the short isoform TRPC4*β*.^114^ However, as noted earlier, elevated calcium levels also promote CaM binding,^21^ which restricts the mobility of the TRP helix and locks the channel in its closed state.^21^

TRPC4 and TRPC5 currents can also be potentiated by increasing extracellular calcium concentrations to 10 mM.^122^ TRPC6 channels are similarly sensitive to free intracellular calcium levels, but their activation is primarily mediated through CaM-dependent kinase II phosphorylation.^80^^,^^123^ Elevated free intracellular calcium concentrations can also promote the translocation of TRPCs to the plasma membrane.^124^ Additionally, higher extracellular calcium levels increase TRPC6 currents.^80^

However, extracellular calcium concentrations exceeding physiological levels (≥3 mM) inhibit TRPC6 channel activity, while TRPC7 currents are inhibited by extracellular calcium even at micromolar concentrations.^80^ TRPC3 currents are similarly suppressed by extracellular calcium.^125^ Altogether, calcium exerts both stimulatory and inhibitory effects on different TRPCs, allowing for precise fine-tuning and regulation of channel function.

### C Expression pattern and primary physiological roles of TRPCs

An overview of the expression profile of TRPCs is provided in Table 1. TRPC1 is ubiquitously expressed across various tissues.^126^^,^^127^ It forms heterotetrameric channels with other TRPC protein subunits,51., 52., 53., 54. and even with other TRP proteins, such as TRPP2,^128^ TRPV4,^58^ and TRPV6.^59^ These interactions alter biophysical properties^54^ and reduce calcium permeability.^51^^,^^59^^,^63., 64., 65.

TRPC1 is highly expressed in neurons, where it plays roles in axonal chemotaxis,^129^ mediates the slow excitatory postsynaptic potential induced by metabotropic glutamate receptors in Purkinje cells,^130^ and provides neuroprotection against exogenous neurotoxins.^131^ Additionally, TRPC1 enhances the differentiation of hippocampal neurons^132^ and promotes the proliferation of neuronal progenitor cells in the hippocampus^133^ and cochlear spiral ganglion.^134^ These findings suggest that TRPC1 is involved in neuronal homeostasis and might play a role in neurodegeneration.^135^

In nonneuronal tissues, calcium influx via TRPC1 in mandibular salivary gland cells enhances salivary secretion.^136^^,^^137^ In the cardiovascular system, TRPC1 promotes proliferation,^65^ influences vascular tone, and is upregulated in smooth muscle and cardiac myocytes after stenosis, leading to enhanced proliferation.^138^^,^^139^ This suggests a potential role for TRPC1 in cardiac dysfunction.^140^ TRPC1 also regulates vascular function, inducing vasoconstriction.141., 142., 143., 144. However, endothelial TRPC1 promotes vasodilation.145., 146., 147. TRPC1/4 heteromers regulate endothelial permeability in the lungs,^148^ and TRPC1 contributes to pulmonary hypertension,^149^^,^^150^ immune system regulation,^151^ cancer progression,^152^ and endocrine functions, such as parathyroid hormone secretion.^153^

TRPC2 channels^75^ are functionally expressed in most macrosmatic species, such as fish and mammals.^154^ However, in microsmatic species, such as hominids and Old World monkeys, TRPC2 is nonfunctional and has evolved into a pseudogene.^15^^,^^16^ This loss is associated with the degeneration of the VNO,^155^ where TRPC2 channels are essential for pheromone perception.^72^^,^^156^

In the VNO, TRPC2 is crucial for pheromone-driven behaviors. TRPC2 deficiency results in impaired pheromone perception, sex-typical (sex is defined as the biological classification of individuals as male or female) brain changes,^157^ and altered social behaviors, including reduced aggression and impaired olfactory sex recognition.^158^^,^^159^ TRPC2-deficient males may exhibit male–male mating behavior,^159^ which is accompanied by reduced dopamine levels in dopaminergic neurons.^160^ Similarly, TRPC2-deficient females may display male-typical sexual behavior.^161^

Outside the VNO, TRPC2 channels have diverse functions. In thyroid cells, TRPC2 may increase thyroid-stimulating hormone receptor expression, reduce thyroglobulin maturation, influence free intracellular calcium and iodide homeostasis, and reduce thyroid cell proliferation.^162^ In spermatozoa, TRPC2 channels enhance the acrosome reaction,^163^ facilitating penetration of the oocyte.^163^ In erythrocytes, TRPC2 channels have been implicated in oxidative stress-induced hemolytic anemia.^164^ Expression in the testis was also demonstrated.^165^

TRPC3 channels are highly expressed in the central nervous system (CNS),^166^ with particularly prominent expression in the pituitary gland and Purkinje cells of the cerebellum.167., 168., 169. TRPC3 is also expressed in the cardiovascular system, notably in the heart^170^ and lungs of patients with pulmonary arterial hypertension.^171^^,^^172^

TRPC3 can form heterotetrameric channel complexes with TRPC6 and TRPC7,^60^^,^^70^ as well as with TRPC1^51^^,^^56^ and TRPC4^71^ protein subunits. Mice deficient in the TRPC3 gene exhibit abnormal extrapyramidal coordination deficits, which are attributed to the absence of TRPC3-mediated calcium influx in Purkinje cells.^167^ A point mutation in the TRPC3 gene (T635A), which leads to increased channel activity,^173^ results in an ataxic phenotype. This mutant mouse line is referred to as "moonwalker."^174^ In these mice, increased TRPC3 activity causes impaired differentiation of Purkinje cells during early postnatal development and extensive degeneration during late postnatal development.^174^

In addition to Purkinje cells, TRPC3 is expressed in unipolar brush cells, which are excitatory interneurons in the cerebellum.^175^^,^^176^ These unipolar brush cells undergo significant degeneration within the first 4 weeks of postnatal development in moonwalker mice.^177^

TRPC3 channels are also expressed in the kidney^178^ and blood vessels. They are found in the endothelium of the low-pressure system, such as umbilical veins^179^ and pulmonary arteries,^180^ as well as in the high-pressure system, including afferent arterioles of the kidney^181^ and coronary arteries.^182^ Notably, TRPC3 channels are highly expressed in the endothelium of high-pressure arteries, where they are implicated in hypertension^181^ and arteriosclerosis.^183^

TRPC3 channels are also expressed in smooth muscle cells of resistance arteries^184^^,^^185^ and cardiomyocytes.186., 187., 188., 189., 190. They may promote cardiac hypertrophy^188^^,^^190^ and fibrosis.^191^ Additionally, TRPC3 channels have endocrine functions, enhancing insulin secretion,^192^ and are involved in cancer, such as ovarian and breast cancer.^193^^,^^194^ TRPC3 also influences tumor energy metabolism^195^ by enhancing mitochondrial calcium uptake.^196^^,^^197^ This mitochondrial mechanism demonstrates that TRPC3 plays important roles both in the plasma membrane and intracellularly.^198^

TRPC4 and TRPC5 channels are expressed in multiple tissues and organs, including the brain,^199^ kidney,^200^^,^^201^ and vascular system.^202^ TRPC4 is highly expressed in the endothelium,^203^ where it regulates endothelial calcium homeostasis. In the CNS, TRPC4 and TRPC5 channels are significantly expressed and involved in neuroplasticity. TRPC4 is present in corticolimbic regions^199^ and midbrain dopaminergic neurons in the ventral tegmental area and substantia nigra.^204^

In peripheral sensory and spinal cord neurons, TRPC4 and TRPC5 contribute to pain, inflammation, and itch.205., 206., 207. TRPC1/4/5 heteromers, particularly TRPC4, are implicated in neurodegeneration^208^ and play a role in morphine tolerance and hyperalgesia.^209^

TRPC5 is primarily expressed in the brain,^168^^,^^199^^,^^210^^,^^211^ where it is associated with neurite growth, neurotransmission, and learning.^61^^,^212., 213., 214. In the amygdala, TRPC4 and TRPC5 mediate strong fear responses to aversive stimuli^214^^,^^215^ and are involved in peripheral-induced neuropathic pain syndromes.^216^ Notably, TRPC5 activation reduces mechanically induced neuropathic pain.^217^

Beyond pain, TRPC5 influences metabolism^218^^,^^219^ and contributes to epileptic activity. In hippocampal CA1 neurons, TRPC5 causes constant membrane depolarizations, known as plateau potentials,^220^ which occur during epileptic seizures.^221^^,^^222^ TRPC5-deficient mice exhibit fewer epileptic seizures.^61^ Additionally, TRPC5 inhibits hippocampal neurite length and growth cone morphology.^213^

TRPC5 is expressed in adrenal chromaffin cells and plays a crucial role in adrenaline secretion, which is essential for preventing hypoglycemia.^223^^,^^224^ TRPC5 also contributes to angiogenesis^225^ and acts as a cold sensor^226^ in the heterologous overexpression system,^227^^,^^228^ the peripheral nervous system,^227^ and in odontoblasts.^229^

TRPC5 and TRPC6 channels exhibit opposing effects on the actin cytoskeleton of podocytes and fibroblasts. Receptor-operated TRPC5 activation leads to the loss of actin stress fibers, resulting in a motile and noncontractile phenotype,^230^ which characterizes podocyte damage and contributes to proteinuria and kidney disease.231., 232., 233. In contrast, TRPC6 activation promotes the formation of actin stress fibers, establishing a contractile and nonmotile phenotype.^230^

TRPC5 channels may also play a role in cancer by enhancing angiogenesis.^234^^,^^235^ Furthermore, TRPC5 contributes to chemotherapy-induced multidrug resistance in tumor cells by increasing the expression of ATP-binding cassette subfamily B member 1 transporters, also known as P-glycoprotein 1 or multidrug resistance protein 1.234., 235., 236. TRPC5 additionally functions as a pressure sensor in aortic baroreceptors, stabilizing blood pressure,^237^ and mediates endothelium-dependent contraction of carotid arteries.^238^^,^^239^

TRPC6 channels are highly expressed in the vasculature, lungs, brain, placenta, spleen, ovaries, small intestine, neutrophils, and podocyte foot processes.^240^ In neurons, TRPC6 channels increase endocannabinoid synthesis,^241^ promote dendrite growth,^242^^,^^243^ and support neuronal plasticity.^243^ TRPC6 is also expressed in extrinsic fibers innervating the intrinsic cardiac ganglia,^244^ olfactory epithelium neurons,^245^ retinal ganglion cells,^246^ and various brain regions, including the cortex, hippocampus, substantia nigra, and cerebellum.^247^

In the vascular system, TRPC6 channels mediate vasoconstriction^248^ and promote vascular smooth muscle cell proliferation.^249^^,^^250^ TRPC6 was proposed to be a direct mechanosensor^251^ mediating myogenic vasoconstriction. However, TRPC6 rather acts as a mechanotransducer with indirect mechanosensitivity.^252^^,^^253^ Nevertheless, TRPC6 may contribute to ultrasound neuromodulation in the brain^254^ and is proangiogenic.^255^^,^^256^ Low extracellular pH activates TRPC6, inhibiting platelet aggregation,^257^ while oxidants, like hydrogen peroxide, not only activate TRPC6 but also increase its membrane expression.^258^

In the kidney, TRPC6 is expressed in glomeruli, tubular cells, and podocytes.^259^ Mutations in the TRPC6 gene result in podocyte damage and are associated with focal segmental glomerulosclerosis (FSGS), a chronic kidney disease leading to end-stage renal failure.^260^^,^^261^ Although the pathomechanism remains unclear, TRPC6 channels in podocyte foot processes, which form the slit diaphragm, are crucial for maintaining calcium homeostasis.

Beyond renal functions, TRPC6 has neuronal roles, including involvement in neurodegeneration and Alzheimer’s disease, and is highly expressed in several cancers.^262^ In the lungs, TRPC6 is found in airway smooth muscle cells,^263^^,^^264^ epithelial cells,^264^ and endothelial cells.^265^ TRPC6 contributes to hypoxic pulmonary vasoconstriction,^263^ lung ischemia-reperfusion (IR)-induced edema,^265^ and lung fibrosis.^266^ In the heart, TRPC6-mediated zinc influx enhances myocardial contractility, suggesting its potential as a therapeutic target for heart failure.^267^^,^^268^ TRPC6, along with TRPC1, TRPC3, and TRPC5, also plays roles in the immune system and phagocytosis.^269^

TRPC7 channels are the least studied TRPCs. They are expressed in the CNS, hypophysis, kidneys,^168^ heart, lungs,^270^ endothelium,^271^ vasculature,^108^^,^^272^^,^^273^ eyes, spleen, and testis.^76^ TRPC7 activation has been linked to an increased breathing rate,^274^ and may contribute to enhanced proliferation in autosomal dominant polycystic kidney disease (ADPKD).^275^ Cardiac TRPC7 channels are implicated in arrhythmias^276^ and myocardial apoptosis.^277^ TRPC7 may also play a role in the pupillary light reflex,^278^ although this remains controversial.^279^

TRPC7 and TRPC6 channels are both involved in phototransduction in retinal ganglion cells, where they are activated downstream of the photosensitive G_q_ protein-coupled receptor melanopsin, leading to PLC*β*4-induced TRPC6/7 activation and cAMP formation.^278^ High TRPC7 expression is associated with the progression of hepatocellular carcinoma^280^^,^^281^ and lung adenocarcinoma.^282^

In summary, TRPCs play vital roles in the regulation of calcium homeostasis and are involved in vascular, neuronal, and kidney functions, sensory transduction, as well as cell migration and proliferation.

### D Human diseases associated with TRPCs

Surprisingly, the global knockout (KO) of all TRPCs results in viable mice that are fertile.^102^^,^^283^^,^^284^ However, a multitude of animal models suggests that TRPCs, in particular but not exclusively, may underlie or aggravate different human diseases through their excessive activity.

A gain-of-function (GOF) mutation in the TRPC3 gene (R762H) is associated with a rare case of autosomal dominant adult-onset spinocerebellar ataxia type 41 (OMIM 616410). Overexpression of this variant in murine neuroblastoma cells leads to neuronal cell death, presumably caused by an increased open probability of the channel,^285^ thereby resembling the phenotype of the mutated channel in the so-called “moonwalker” mouse.^174^

TRPC5 is frequently discussed as a potential therapeutic target for treating kidney diseases, anxiety, and depression.^214^^,^^286^ In 2014, Mignon-Ravix et al^287^ described a loss-of-function (LOF) mutation in the TRPC5 gene associated with X-linked intellectual disabilities (OMIM 300982). Subsequently, additional missense variants in TRPC5, resulting in either constitutively open or nonfunctional channels, were linked to cases of intellectual disabilities, anxiety, and autism.^288^^,^^289^ Moreover, TRPC5 variants are associated with severe childhood-onset obesity, suggesting a potential role for TRPC5 in the regulation of food intake.^288^

TRPC6 dysfunction, resulting from gene mutations or upregulation of its expression, is best understood in the context of pulmonary and renal diseases. In the kidney, several GOF mutations in the TRPC6 gene are closely linked to an autosomal-dominant form of FSGS2 (OMIM 603965),^290^^,^^291^ a rare progressive disease that ultimately leads to kidney failure due to progressive scarring of the glomeruli. Notably, LOF mutations in the TRPC6 channel cause a similar phenotype, particularly in juvenile forms of the disease.^292^ Furthermore, an increased TRPC6 expression compared with healthy individuals was observed in podocytes of patients with diabetic kidney disease.^293^ In the lung, studies using mouse models suggest that TRPC6 is essential for the regulation of hypoxia-mediated pulmonary vasoconstriction and pulmonary hypertension.^263^^,^^294^ In humans, a single-nucleotide polymorphism (SNP) in the TRPC6 promoter region, which leads to elevated basal TRPC6 expression, is associated with an increased risk of idiopathic pulmonary hypertension.^171^^,^^295^ Subsequently, Pousada et al^296^ identified 3 more TRPC6 SNPs in the 5'-untranslated region of the TRPC6 gene that were significantly more common in a cohort of patients with idiopathic pulmonary hypertension compared with the control group. Several mouse models of heart disease suggest an important role for TRPC3 and TRPC6 channels in the development of cardiac hypertrophy.^297^^,^^298^ Relating thereto, a study recently demonstrated an association between elevated TRPC6 expression and a higher risk of heart failure after chemotherapy with the cardiotoxic drug doxorubicin.^299^

For TRPC1, TRPC4, and TRPC7 channels, only weak links between human pathologies and channel dysfunction have been reported to date.

### E Pharmacological modulators of TRPCs

In recent years, the availability of pharmacological modulators of TRPCs has substantially advanced from drugs acting on a range of TRPC isoforms to compounds acting more selectively on distinct TRPC isoforms, with some exceptions; most of the published TRPC blockers still do not sufficiently discriminate between TRPC1/4/5 or TRPC3/6/7. However, combining high-resolution cryo-EM with mutagenesis approaches has recently led to a much better understanding of how drugs modulate TRPC activity, which may facilitate the identification of selective and potent TRPC modulators in the future. Table 2^18^^,^^28^^,^^49^^,^^201^^,^^297^^,^^298^^,^300., 301., 302., 303., 304., 305., 306., 307., 308., 309., 310., 311., 312., 313., 314., 315., 316., 317., 318., 319., 320., 321., 322., 323., 324., 325., 326., 327., 328., 329., 330., 331., 332., 333. provides an overview of TRPC modulators.

**Table 2:** Pharmacological modulators of TRPCs.

Name (PubChem CID^a^)	Effect	References
**TRPC1,4,5**		

**Inhibitors**		

ML204 (230710)	Inhibition mTRPC4 IC_50_ = 2.9 *μ*M; mTRPC5 IC_50_ = 10 *μ*M	^300^
AC-1903 (667146)	Inhibition TRPC5 IC_50_ = 14.7 *μ*M	^201^
Clemizole (2782)	Inhibition mTRPC5 IC_50_ = 1–1.3 *μ*M; mTRPC4 IC_50_ = 6 *μ*M	^301^
Duloxetine (60835)	Inhibition hTRPC5 IC_50_ = 0.54 *μ*M	^302^
Pico145 (85473438)	Inhibition hTRPC4 IC_50_ = 0.35 nM; hTRPC5 IC_50_ = 1.3 nM	^303^
HC-070 (85473309)	Inhibition hTRPC4 and hTRPC5 IC_50_ = 0.35–3.4 nM	^304^
GFB-8438 (138471783)	Inhibition hTRPC5 IC_50_ = 0.18 *μ*M; hTRPC4 IC_50_ = 0.29 *μ*M	^305^

**Activators**		

Riluzole (5070)	Activation mTRPC5 IC_50_ = 9.2 *μ*M	^306^
Methylprednisolone (6741)	Activation mTRPC5 EC_50_ = 12 *μ*M	^307^
BTD (46369355)	Activation mTRPC5 EC_50_ = 1.4 *μ*M	^307^
(–)-Englerin A (46242512)	Activation hTRPC5 EC_50_ = 7.6 nM; hTRPC4 IC_50_ = 11.2 nM	^308^
AM237 (90403462)	Activation hTRPC5 EC_50_ = 15–20 nM	^309^
GFB-887 (N/A)	Inhibition TRPC5, in clinical trials	^310^
BI 1358894 (N/A)	Inhibition TRPC4; TRPC5, in clinical trials	^311^

**TRPC3,6,7**		

**Inhibitors**		

Pyr3 (56964346)	Inhibition mTRPC3 IC_50_ = 0.7 *μ*M	^312^
Pyr10 (53475435)	Inhibition TRPC3 IC_50_ = 0.72 *μ*M	^313^
Compound 20 (C20, JW-65) (162659202)	Inhibition hTRPC3 IC_50_ = 0.37 *μ*M	^314^
60a (N/A)	Inhibition hTRPC3 IC_50_ = 90 nM	^315^
GSK2833503A (71818575)	Inhibition rTRPC6 IC_50_ = 3 nM; rTRPC3 IC_50_ = 21 nM	^297^^,^^316^
GSK2332255B (71818573)	Inhibition rTRPC6 IC_50_ = 4 nM; rTRPC3 IC_50_ = 5 nM	^297^^,^^316^
SAR7334 (53378752)	Inhibition hTRPC6 IC_50_ = 9.5 nM; hTRPC3 IC_50_ = 282 nM; TRPC7 IC_50_ = 226 nM	^317^
AM-1473 (167993650)	Inhibition hTRPC6 IC_50_ = 0.2 nM	^28^
Larixyl acetate (11957828)	Inhibition hTRPC6 IC_50_ = 0.58 *μ*M; hTRPC3 IC_50_ = 6.83 *μ*M	^318^
SH045 (134611888)	Inhibition hTRPC6 IC_50_ = 62 nM; hTRPC3 IC_50_ = 0.84 *μ*M	^319^
BI 749327 (138377580)	Inhibition mTRPC6 IC_50_ = 13 nM; orally bioavailable	^298^
BTDM (162423070)	Inhibition hTRPC6 IC_50_ = 10 nM	^18^
DS88790512 (138319685)	Inhibition hTRPC6 IC_50_ = 11 nM; orally bioavailable	^320^
BI 764198 (138674835)	Inhibition hTRPC6, in clinical trials	^321^

**Activators**		

PPZ1 (6462584), PPZ2 (6465626)	Activation mTRPC3/6/7 nonselective	^322^
GSK1702934A (16376051)	activation hTRPC3 EC_50_ = 80 nM; hTRPC6 EC_50_ = 440 nM	^323^
Compound 4n (N/A)	Activation hTRPC3 EC_50_ = 20 nM; mTRPC7 EC_50_ = 90 nM *μ*M; mTRPC6 EC_50_ = 1.39 *μ*M	^324^
Artemisinin (68827)	Activation hTRPC3 EC_50_ = 30–50 *μ*M	^325^
AM-0883 (145997911)	Activation hTRPC6 EC_50_ = 46 nM	^28^
M085 (N/A)	Activation hTRPC6, mTRPC6 EC_50_ = 3.8 *μ*M	^326^
C20 (N/A)	Positive allosteric modulator TRPC6	^327^
PhoDAG-1 (121225613) PhoDAG-3 (121225610)	Photoswitchable activator hTRPC6, mTRPC6; mTRPC2	328., 329., 330.
OptoDArG (131954527)	Photoswitchable activator hTRPC3; mTRPC6	^49^^,^^330^
OptoBI-1 (146018968)	Photoswitchable activator hTRPC3; mTRPC6, hTRPC6, hTRPC7	^330^^,^^331^
BTDAzo (N/A)	Photoswitchable activator mTRPC5	^332^
dfdc-OptoBI-1 (N/A)	Photoswitchable activator mTRPC6	^333^

a PubChem Compound Identification number. N/A – not available.

#### 1 Inhibitors of TRPC1/4/5 channels

The first identified inhibitors of TRPC4 and TRPC5 channels discriminated poorly between the 2 isoforms and were of low potency. ML204 inhibits TRPC4 channels (IC_50_ = 2.9 *μ*M) with a 3-fold preference over TRPC5 (IC_50_ = 10 *μ*M) and a 19-fold selectivity over TRPC6.^300^ In a transgenic rat model of FSGS with podocyte-specific overexpression of the angiotensin II AT_1_ receptor, intraperitoneal application of ML204 suppressed proteinuria and prevented podocyte loss.^201^ AC-1903, which inhibits TRPC5 less potently (IC_50_ = 14.7 *μ*M) but does not inhibit TRPC4 or TRPC6 channels, was also effective in the transgenic rat model mentioned above and in a model of hypertension-induced FSGS (Dahl salt-sensitive rats), reducing proteinuria and protecting podocytes.^201^ However, the pathogenic role of TRPC5 in podocytes was recently called into question.^334^

The antihistamine clemizole displays a 6-fold preference for TRPC5 (IC_50_ = 1–1.3 *μ*M) over TRPC4 (IC_50_ = 6 *μ*M).^301^ Cryo-EM has revealed the binding site of clemizole, which is located within the VSLD of TRPC5.^24^ Duloxetine, an antidepressant that is also effective in the treatment of neuropathic pain, inhibits TRPC5 channels (IC_50_ = 0.54 *μ*M) by fitting into the same binding pocket.^302^

In comparison, the xanthine-based compound Pico145 (HC-068) is considerably more potent, inhibiting TRPC1/4/5 channels with an IC_50_ of 1.3 nM and 0.35 nM for TRPC5 and TRPC4, respectively.^303^ Its close analog, HC-070, blocks homo- and heteromeric TRPC4/5 channels with IC_50_ values between 0.3 and 3.4 nM.^303^^,^^304^ The cryo-EM structure of the human homomeric TRPC5 channels in the presence of Pico145 identified the binding of the drug to lipid binding site 2 between individual TRPC5 subunits, displacing a lipid upon binding of the drug.^25^ This binding site, which is highly conserved within the TRPC family,^25^ was also determined for the Pico145-bound TRPC1/4 heteromer,^30^ and further confirmed by Song et al^24^ for the binding of HC-070 to TRPC5. HC-070 is effective in animal models of neurological diseases, as oral administration in mice allows the compound to cross the blood-brain barrier, exerting antidepressant and anxiolytic effects.^304^ Moreover, intraperitoneal administration of HC-070 reverses cognitive and motor deficits in rat models of Parkinson's disease.^335^^,^^336^

Screening of a 400,000-compound library and subsequent hit optimization led to the discovery of several pyridazinone-based inhibitors, with GFB-8438 being the most promising regarding its physicochemical properties. GFB-8438 inhibits TRPC5 (IC_50_ = 0.18 *μ*M) with a similar potency to TRPC4 channels (IC_50_ = 0.29 *μ*M).^305^ Cryo-EM studies performed on TRPC4 homomers demonstrated the binding of GFB-8438 and closely related compounds, GFB-9289 and GFB-8749, to the VSLD of TRPC4.^21^ In the deoxycorticosterone acetate-salt rat model of hypertension and renal inflammation, GFB-8438 exerts nephroprotective effects, evident by reduced protein and albumin concentrations in the urine.^305^

#### 2 Inhibitors of TRPC3/6/7 channels

TRPC3 is, at least within the TRPC family, selectively inhibited by pyrazole compounds Pyr3 and Pyr10 (IC_50_ = ∼0.7 *μ*M).^312^^,^^313^ In vivo, Pyr3 reduces cardiac hypertrophy and transition to heart failure in mice subjected to pressure overload,^312^ whereas Pyr10-mediated TRPC3 inhibition alleviates systemic inflammatory responses in mice after treatment with lipopolysaccharide.^197^ However, both drugs also inhibit ORAI1 channels, obscuring the attribution of their beneficial effects to individual channel blockage.^313^ Structural optimization of Pyr3 results in the development of compound C20 (JW-65), a derivative with increased metabolic stability and low toxicity, which retains similar potency for TRPC3 inhibition (IC_50_ = 0.37 *μ*M), while exhibiting improved selectivity over ORAI1 channels.^314^ Based on the same lead structure, compound 60a, with a 4-fold improvement in potency, was later synthesized.^315^

The aminothiazole GSK2833503A (GSK503A) potently inhibits TRPC3 and TRPC6 with a higher selectivity for TRPC6 (IC_50_ = 3 nM) over TRPC3 (IC_50_ = 21 nM), whereas GSK2332255B (GSK255B) inhibits both TRPC3 and TRPC6 with a similar potency (IC_50_ = 3–4 nM).^297^^,^^316^ Both drugs reduce hypertrophy and fibrosis in a model of cardiac hypertrophy, possibly by acting on both channels.^297^

SAR7334 was identified through a pharmacophore-guided design of aminoindanol derivatives based on the broad TRP channel blocker SKF96365. SAR7334 predominantly inhibits TRPC6 but also TRPC3 and TRPC7 channels with IC_50_ values of 9.5, 282, and 226 nM, respectively, and suppresses hypoxic pulmonary vasoconstriction in explanted mouse lungs exposed to hypoxic conditions.^317^ Based on SAR7334, the most potent and selective TRPC6 inhibitor to date, AM-1473, was developed (IC_50_ = 0.2 nM),^28^ which binds to a pocket formed by the cytoplasmic portions of S1–S4 and the TRP helix.^28^

Larixyl acetate, a diterpenoid from larch resin, primarily inhibits TRPC6 channels with a 10-fold selectivity for TRPC6 (IC_50_ = 0.6 *μ*M) over TRPC3. It effectively prevents acute hypoxia-induced vasoconstriction in isolated lungs from mice^318^ and offers protection against pressure overload-induced heart failure.^337^ Subsequent structural optimization yielded the methylcarbamate derivative SH045 with an improved potency for TRPC6 (IC_50_ = 62 nM). SH045 reduced edema in an animal model of lung IR^319^ and ameliorated renal fibrosis in obese mice after unilateral ureteral obstruction.^338^

BI 749327 is an orally bioavailable TRPC6 blocker (IC_50_ = 13 nM) with high selectivity.^298^ Due to its favorable physicochemical properties, the compound has been tested in several animal models. The administration of BI 749327 improved heart function and reduced fibrosis in mice subjected to pressure overload, and reduced renal fibrosis in a renal injury model.^298^ Moreover, in a mouse model of severe Duchenne muscular dystrophy, TRPC6 inhibition by BI 749327, starting from day P3, improved skeletal and cardiac muscle function and survival in mice.^339^

Other highly potent TRPC6 inhibitors include the high-affinity TRPC6 inhibitor BTDM (IC_50_ = 10 nM), which binds at the interface between the pore and VSLD,^18^ and orally bioavailable DS88790512 (IC_50_ = 11 nM).^320^ However, neither of these compounds has been tested in vivo so far.

#### 3 Activators of TRPC4/5 channels

Riluzole, which is the only Food and Drug Administration (FDA)-approved drug to treat amyotrophic lateral sclerosis, activates TRPC5 with low potency (EC_50_ = 9.2 *μ*M) but is, at least within the TRPC family, specific for TRPC5.^306^ A screening approach by Beckmann et al^307^ identified methylprednisolone (EC_50_ = 12 *μ*M) and the benzothiadiazine derivative (BTD) (EC_50_ = 1.4 *μ*M) as novel TRPC5 agonists. Notably, BTD alleviated mechanical allodynia in diabetic peripheral neuropathic rats, presumably via the downregulation of TRPC5 expression and anti-inflammatory and antiapoptotic effects of BTD.^217^

(–)-Englerin A, derived from the bark of the *Phyllanthus engleri* tree, displays the highest potency and efficacy and activates both TRPC4 and TRPC5 in low nanomolar concentrations (EC_50_ = 11.2 nM and 7.6 nM for TRPC4 and TRPC5, respectively).^308^ However, (–)-englerin A is lethal in rodents when administered at concentrations near those required to activate TRPC4, likely due to excessive TRPC4 activation leading to pulmonary edema.^340^

Following up on the structure of the TRPC1/4/5 blocker Pico145, Minard et al^309^ recently synthesized the analog AM237, which potently activates homomeric TRPC5 (EC_50_ = 15–20 nM) but not heteromeric TRPC1/5, TRPC4/5, or homomeric TRPC4 channels.

#### 4 Activators of TRPC3/6/7 channels

Small molecules that activate TRPC3 channels include piperazine-derived compounds^322^ PPZ1 and PPZ2, which do not discriminate well between TRPC3/6/7 and GSK1702934A, a potent TRPC3/6 activator (EC_50_ = 80 and 440 nM for TRPC3 and 6, respectively).^323^ Qu et al^324^ developed a series of pyrazolopyrimidine-derived TRPC3/6/7 agonists with a preference for TRPC3, of which compound 4n was the most potent (EC_50_ = 20 nM). The antimalarial drug artemisinin activates TRPC3 with low potency (EC_50_ = 30–50 *μ*M) but with a high preference for TRPC3 over TRPC6 and TRPC7.^325^

The TRPC6 activator AM-0883 is highly potent (EC_50_ = 46 nM) with a binding site between the PH and the S6 helix of the adjacent subunit,^28^ which is similar to that of HC-70 and Pico145 in the TRPC1/4/5 channels. The same binding site is targeted by structurally distinct TRPC6 activators M-085 (EC_50_ = 3.8 *μ*M) and GSK1702934A.^326^ In addition to TRPC6 channel activators, the substance C20 was identified, which acts as a positive allosteric modulator, enabling TRPC6 current increases in the presence of 1-oleoyl-2-acetyl-sn-glycerol.^327^

#### 5 Optical control of TRPCs

Recently, compounds have been developed for the precise optical control of TRPCs. These photoswitches are generated by linking a light-sensitive azobenzene moiety to a known channel modulator, enabling light of distinct wavelengths to switch the compound’s activity on and off. Photoswitchable DAGs, such as PhoDAG^328^ and OptoDArG,^49^ are used to rapidly activate DAG-sensitive TRPCs: photoswitchable DAGs are switched on to their active *cis-*isomer upon exposure to 370 nm UVA light and off through *trans-*isomerization at 460 nM. In the *cis* configuration, PhoDAG1 and the more membrane-permeant PhoDAG3 activate TRPC2 in mouse vomeronasal sensory neurons.^329^^,^^341^ *Cis-*PhoDAG1 also activates heterologously expressed TRPC6 channels.^329^^,^^330^^,^^341^ Another photoswitchable DAG, OptoDArG, features 2 photoswitchable azobenzene moieties and is active in the *cis-*form at 365 nm and inactive at 430 nm. OptoDArG enables optical control of TRPC2, TRPC3, and TRPC6 channels upon photoisomerization.^49^^,^^330^^,^^341^

Based on the TRPC3/6 agonist GSK1702934A, Opto-BI-1 was developed to enable optical control of TRPC3 channels in human vascular endothelial cells and mouse hippocampal neurons,^331^ as well as the precise control of TRPC6 channel function.^330^ More recently, BTD served as a starting point for the generation of the photoswitchable TRPC5 agonist BTDAzo, which can control TRPC5 channels in isolated cells and mouse brain slices (EC_50_ = 1.5 *μ*M).^332^ In the future, it will be fascinating to explore whether photoswitchable TRPC modulators can also be applied in vivo. A crucial step toward the in vivo application of photopharmaceuticals is the development of red-light switchable compounds, such as the recently developed dfdc-OptoBI-1.^333^ Red light is nonphototoxic and offers greater tissue penetration, making it particularly suitable for biomedical applications.

### F Ongoing or completed clinical trials with TRPCs as therapeutic targets

To date, only a few clinical trials have been initiated that use small molecules targeting TRPCs. Considering their prominent role in lung and kidney diseases, TRPC5 and TRPC6 have emerged as the most promising therapeutic targets. The TRPC5 inhibitor GFB-887 is well tolerated in healthy patients (phase 1 study; NCT03970122).^310^ It was further tested in patients with FSGS (NCT04950114) and those suffering from diabetic nephropathy or FSGS (NCT04387448) to evaluate the possible beneficial effect of GFB-887 on kidney function. However, both studies were terminated due to business reasons, and no results have been published to date.

The TRPC4/5 channel inhibitor BI 1358894 has recently been explored as a potential treatment for psychiatric disorders, including depression and anxiety. Its safety, tolerability, and pharmacokinetics were demonstrated in 2 phase 1 studies (NCT03210272 and NCT03754959).^342^ However, phase 2 trials assessing the efficacy of BI 1358894 in patients with major depression (NCT04423757), post-traumatic stress (NCT05103657), and borderline personality disorders (NCT04566601) did not show efficacy of the drug.^343^^,^^344^ Nonetheless, the outcome of another phase 2 trial (NCT04521478) investigating its efficacy in patients with major depression who showed an inadequate response to standard antidepressants is still awaited.

The TRPC6 inhibitor BI 764198 was well tolerated in 4 phase 1 studies (NCT03854552, NCT04102462, NCT04656288, and NCT04176536) and is currently being investigated in individuals with FSGS in a phase 2 trial (NCT05213624).^345^ In another phase 2 trial (NCT04604184), the same drug failed to reduce the risk and/or severity of acute respiratory distress syndrome during the course of the COVID-19 disease.^321^ Additionally, an observational study (NCT05507879) is currently exploring whether TRPC6 variants can predict chemotherapy-related cardiomyopathy and heart failure in breast cancer patients.

## III TRPVs

### A TRPV gene family

**Fig. 3:**
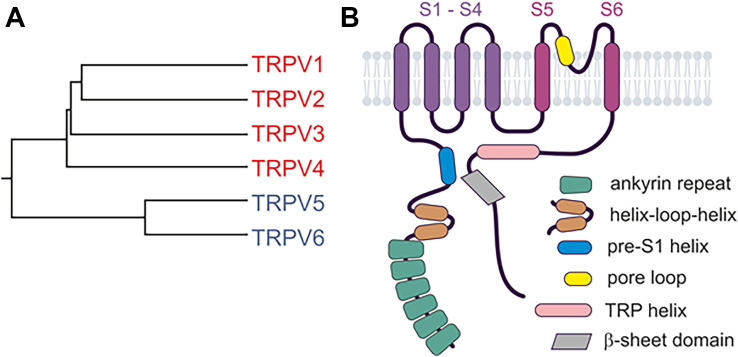
Phylogenetic tree and domain topology of TRPVs. (A) Phylogenetic tree of the human TRPV1–6 proteins. (B) Domain architecture of TRPV monomers.

The TRPV gene family consists of 6 distinct members: TRPV1–6 (Table 1), which can be categorized into 2 main subgroups based on their homology and functional characteristics: the thermosensitive channels TRPV1–4, which are nonselective for monovalent cations, and the Ca^2+^-selective channels TRPV5 and TRPV6 (reviewed in Vennekens et al^346^) (Fig. 3A).

TRPVs, especially TRPV1, have been extensively studied and have emerged as promising drug targets for treating various human diseases. In this chapter, we will provide an overview of the key advancements in understanding TRPV characteristics and their roles in health and disease. We will introduce and discuss modulators of TRPVs and their applicability in animal and human disease models. However, due to their abundance, the focus will be on compounds that have already been well validated and are characterized by relatively high specificity and potency.

### B Domain topology, assembly, and functional characteristics of TRPVs

#### 1 Domain topology of TRPVs

Cryo-EM and crystallographic studies have provided structures for all homotetrameric mammalian TRPV complexes, including numerous structures in their ligand-bound or CaM-bound states. Overall, the available homotetrameric TRPV structures display a rotationally symmetric subunit assembly with protein moieties mostly extending into the cytoplasmic space and only minor extracellular bulges formed by short loops that connect the TM-spanning segments S1–S6 and the pore loop, which is intercalated between S5 and S6 (Fig. 3B).

Several expert reviews have elaborated on common or distinct structural features of specific TRPV isoforms and their contribution to the regulatory and biophysical properties of the respective channels.347., 348., 349., 350., 351., 352., 353., 354., 355., 356., 357. Table 1 summarizes the available PDB entries for TRPVs in their apo or ligand-bound states.

The intracellular N- and C-termini of TRPVs are joined by TMDs that are organized in a similar fashion as in TRPCs. A bundle composed of the first 4 TM-spanning helices forms a VSLD, which connects via the *α*-helical S4–S5 linker to a second fold, consisting of S5, a re-entrant short pore loop, and S6. The N-termini of TRPVs contain an ARD with 6 consecutive ankyrin repeats that, in some but not all TRPV isotypes, engage in contact with neighboring subunits. The ARD is followed by 2 *α*-helices that are referred to as the helix-loop-helix linker domain and a pre-S1 *α*-helix.

Forming a sharp turn, the S6 of the TMD is connected to the cytosolic C-terminus by a TRP domain, which contains an *α*-helix that is oriented parallel to the plasma membrane, and intimately contacts the S4–S5 linker as well as the pre-S1 helix, and is supposed to undergo a rotational movement during channel opening.^358^^,^^359^ Following the TRP helix, a *β*-sheet–containing domain engages in additional interactions with the N-terminus of the same channel subunit.

Structural motifs within the permeation pathway include a cone-shaped vestibule at the outer mouth of the pore, which exposes negatively charged amino acids and may attract cations, engage in salt bridges with neighboring subunits, or become protonated under acidic extracellular conditions, eg, in inflamed tissues.^360^^,^^361^ Notably, the strong electronegativity of the vestibule of TRPV6 has been proposed to resemble that of *Drosophila* Orai, thereby providing a common mechanism for divalent-selective permeation.^362^

The selectivity filter of the TRPV1–4 subgroup shares an I-G-M/L-G-D/E motif, whereas the Ca^2+^-selective TRPV5 and TRPV6 channels display a distinct L-T-V/I-I-D amino acid sequence. The latter is located in the second part of the pore loop, which is centrally positioned and kinks back from a short PH to form an outward-pointing vertical stack of amino acids that narrows down the entry pathway and coordinates influxing cations in 1 or 2 sites before releasing them to another coordination site located within an inner cavity that leads to the lower gate.^363^^,^^364^ In all human TRPVs, the outer vestibule narrows down to the selectivity filter with an aspartic acid (or glutamic acid in TRPV2), possibly expelling anions, and representing a first landing platform-like coordination site for influxing cations in the upper part of the selectivity filter. Since neutralization of this anionic amino acid in TRPV1 or TRPV4 not only reduces the permeability of divalent cations, but also lowers the potency of ruthenium red-mediated channel block, it has been recognized early as part of the binding site of the polycationic open pore blocker,^365^^,^^366^ a concept that has been confirmed by structural analyses.^367^ In the Ca^2+^-selective isoforms TRPV5 and TRPV6, the upper coordination site may bind divalent cations more tightly, thereby causing longer occupancy times and contributing to repulsive forces between stacked divalent cations that would allow a “knock-on” mechanism of Ca^2+^-selective permeation.^364^ The second coordination site, formed in the central and lower parts of the selectivity filter, opens toward an inner cavity that is flanked by residues and backbone carbonyls within S6. Owing to their inverted teepee-like helix bundling and crossing, the S6 segments constrict the pore diameter to form the inner gate. The opening of the inner gate involves reorientation within the S6, characterized by a partial *α*- to *π*-helical rearrangement, which allows rotation of the lower part of S6.^361^^,^^368^ Depending on the channel isoform and the applied activators, TRPVs can adopt several open states, some of which feature a pore radius of more than 2 Å, allowing the permeation of large organic cations, such as NMDG^+^, YoPro-1, MEQ^+^, or QX-314.369., 370., 371., 372. Like in TRPCs, the VSLD of TRPVs can harbor lipids in positions that, in some cases, overlap with ligand binding sites.^352^^,^^368^^,^373., 374., 375., 376.

#### 2 Assembly of TRPV complexes

Structural, biophysical, and functional analyses have demonstrated that all TRPV isoforms are capable of forming homomeric complexes, yielding functionally active cation channels. Heteromeric TRPV assemblies can form between the closely related isoforms TRPV5 and TRPV6, but also between TRPV1 and TRPV2.377., 378., 379. Other studies found a more promiscuous pattern of heteromerization between the heat-sensitive TRPV1–4 subunits,380., 381., 382. or heteromeric complexes across different TRP channel families, such as TRPV1 and TRPA1,^383^ TRPV2 and TRPP2,^384^ TRPV4 and TRPC1^145^^,^^385^ or TRPC1/TRPP2,^386^ TRPV5 and TRPML3,^387^ or TRPV6 and TRPC1.^59^ At present, the relevance of heteromeric TRPV complexes is not yet sufficiently understood, and heteromer-specific pharmacological tools are lacking.

As an exception to the rule that TRP channels assemble as tetramers, a small fraction of purified and reconstituted TRPV3 channel subunits has been shown to transiently engage in a noncanonical pentameric assembly when studied by high-speed atomic force microscopy under specific stimulation conditions, and its properties as a dilated pore conformation have been proposed.^388^ The proof that electrophysiological single channel recordings actually show currents through a pentameric TRPV3 complex is, however, lacking, and the experimental settings have been critically commented.^389^

Finally, assemblies between TRPVs and auxiliary subunits or temporary interaction partners may contribute to the regulation of channel activity, plasma membrane targeting, internalization, or degradation. The plasma membrane stability and lifetime of TRPV1 have been found to be positively modulated by physical interaction with the toll-like receptor 4,^390^ and by the interaction with Kv*β*1, a non–pore-forming subunit of voltage-gated potassium channels.^391^ Functional interactions between TRPV1, TRPV3, and TRPV4 with associated anoctamin 1, a Ca^2+^-activated Cl^–^ channel, have been found to enhance capsaicin-evoked nociception, promote wound healing, secretion from exocrine glands, and release of vasodilatory factors from endothelial cells.^392^ In keratinocytes, TRPV3 has been shown to form a complex with the EGF receptor, which is associated with a mutual augmentation of functional activities.^393^ Another interaction of TRPV3 with TMEM79 was demonstrated to decrease the plasma membrane abundance of TRPV3 by promoting its degradation.^394^ Likewise, trafficking of TRPV2 and TRPV4 has been proposed to be regulated by their respective interactomes, as recently reviewed.^395^ TRPV4 physically interacts with the small GTPase RhoA, which dampens the TRPV4 activity unless disease-related mutations in either TRPV4 or RhoA prevent their assembly.^396^^,^^397^

Physical interactions of TRPV5 channels involve the catalytic processing by the extracellular *β*-glucuronidase klotho,^398^ and an intracellular interaction with the Ca^2+^-buffering protein calbindin-D(28K).^399^ A serine-threonine kinase with-no-lysine 4-dependent forward trafficking from the Golgi apparatus to the plasma membrane has been described as a result of fibroblast growth factor-23 signaling.^400^ The apical plasma membrane trafficking of TRPV5 may be further stabilized by interactions with the multi-PDZ domain protein NHERF2,^401^ and by a second noncatalytical function of the soluble extracellular domain of *α*-klotho to connect TRPV5 with the membrane protein galectin-1.^402^ Both TRPV5 and TRPV6 have been shown to interact with Rab11a, which targets the channels to the plasma membrane,^403^ where they might be concentrated in the apical membrane of polarized cells by interacting with the PDZ domain-bearing scaffolding protein NHERF4.^404^ Finally, a physical interaction of TRPV6 with the protein tyrosine phosphatase PTP1B has been shown to remove a Src-mediated tyrosine phosphorylation, thereby dampening the channel activity when studied in a heterologous expression system.^405^

In the future, more studies of TRPV interactomes, applying quantitatively accurate and unbiased methodologies like those recently presented^62^ for TRPC1, may provide additional hypotheses to unravel functionally relevant macromolecular assemblies involving TRPVs.

#### 3 Functional characteristics of TRPV complexes

Based on sequence homology and functional properties, TRPVs can be subdivided into 2 subgroups. The TRPV1–4 subgroup forms warmth- or heat-activated, Ca^2+^-permeable, but poorly selective (pCa/pNa = 2–10) cation channels that typically share an outwardly rectifying current voltage relationship. By contrast, TRPV5 and TRPV6 form Ca^2+^-selective channels that are at least, to some degree, spontaneously active, but not activated by heat, and give rise to inwardly rectifying ionic currents.

The founding member, TRPV1, is a prototypical temperature sensor that is further sensitized by inflammatory mediators, chemical ligands, or low extracellular pH. The heat- or capsaicin-induced gating mechanism of TRPV1 is characterized by a uniquely large shift of its voltage-dependent activation curve from nonphysiological positive potentials to more negative potentials.^406^ Since large shifts in half-maximally activating membrane potentials are a common feature within thermally activated TRP channels, including TRPV3, TRPM4, and TRPM8, and since activating ligands can cause similar shifts in these channels, an atypical voltage sensor with a small gating charge has been proposed to integrate various inputs by shifting the window of voltage-dependent gating into the range of physiological resting membrane potentials.^407^

The temperature thresholds of heterologously expressed human TRPV1–4 channels observed at membrane potentials of –60 mV to –100 mV in quiescent cells scale between mild warmth of 23–39 °C for TRPV3 and TRPV4,^408^^,^^409^ to moderate heat of above 43 °C for TRPV1.^410^ Other than the rat or mouse TRPV2 orthologs, which are activated at noxious hot temperatures of >52 °C, human TRPV2 has been found to be heat-insensitive.^411^^,^^412^ Notably, TRPV1 orthologs that are isolated from species adapted to lower or higher temperatures can display corresponding changes in temperature-dependent channel gating, with higher temperature thresholds found in camel or ground squirrel TRPV1,^413^ while TRPV1 in amphibians or zebrafish is tuned to lower temperatures.414., 415., 416. These thresholds shall not be considered as absolute values because they are voltage-dependent and modulated by ligands, second messengers, or during repeated activation cycles. Conditions that mimic inflammation, such as stimulation of PLC- and PKC-coupling bradykinin receptors or cAMP-elevating prostaglandin receptors or extracellular acidification, lower the temperature threshold for TRPV1 activation. Similarly, capsaicin^417^ or piperine,^418^ the pungent ingredients of chili pepper and black pepper, respectively, ethanol,^419^ or certain spider^420^^,^^421^ and scorpion^422^^,^^423^ venoms shift the activation threshold to temperatures that are well below physiological values. Finally, anandamide and structurally related endovanilloids act as activators or positive modulators of TRPV1 channel activity.^424^ Hence, TRPV1 is a polymodal sensor that integrates physiological, pathophysiological, and alimentary or toxic stimuli. Similar changes in temperature thresholds have been reported for repeatedly activated TRPV3 with and without additional sensitization by cholesterol supplementation.^408^^,^^425^^,^^426^

Since local temperatures are elevated by inflammation-associated hyperemia, TRPV1 strongly contributes to constant pain sensation and confers a major component of thermal inflammatory hyperalgesia. The underlying mechanisms have been studied in impressive detail. Mutagenesis studies have revealed that protonation of E600 in the loop that links S5 with the pore loop is the most likely candidate to initiate TRPV1 sensitization in tissue acidosis.^360^ In sensory neurons, TRPV1 modulation via stimulation of G protein-coupled receptors (GPCRs) can either enhance or mitigate thermal or capsaicin-induced responses. While G_q_-coupled B_1_ bradykinin and EP_1_ prostaglandin receptors, as well as G_s_-coupled EP_4_ or IP prostaglandin receptors, sensitize TRPV1 to lower temperatures,427., 428., 429. G_i_-coupled *μ* opioid^430^ or GABA_B1_ receptors^431^ dampen the TRPV1 channel activity.

When strongly activated for longer time periods, TRPV1–4 channels tend to change their permeation properties, allowing penetration of organic cations. This behavior has been referred to as pore dilation, as recently reviewed.^432^ It is of pharmacological interest that large pore diameters found in TRPV1 allow the penetration of cationic tool compounds and drugs, such as the organic cation NMDG^+^, the quinolinium-based chloride indicator dyes MEQ^+^ and MQAE^+^, the DNA stain YoPro-1, and even local anaesthetics.^369^^,^^371^^,^^433^ While some observations and conclusions may be restricted to prolonged activation of strongly overexpressed channels in heterologous expression systems, leading to unwanted changes in intracellular cation concentrations,^434^ the development of large TRPV1 pore diameters that allow the permeation of organic cations has been confirmed by structural biology approaches.^361^^,^^435^

In stark contrast to TRPV1–4, TRPV5 and TRPV6 are highly selective for Ca^2+^ with pCa/pNa > 100 for both channels,^436^^,^^437^ indicating their specialized function in cellular Ca^2+^ transport. However, under divalent-free conditions, they become permeant to monovalent cations,^436^^,^^438^ such as Na^+^ and K^+^. The regulation of TRPV5 and TRPV6 activity involves various intracellular signaling pathways and extracellular factors, whereby PIP_2_ and extracellular Ca^2+^ play a decisive role. TRPV5 and TRPV6 are constitutively active in the presence of PIP_2_, which stabilizes the channel in its open configuration.^439^^,^^440^ Both channels are also sensitive to extracellular pH, with protons acting as potent inhibitors,441., 442., 443. and recently, cryo-EM structures revealed that the proton-dependent block of TRPV5 is caused by a disruption of the PIP_2_ binding pocket, thereby preventing PIP_2_ binding to TRPV5.^444^

TRPV5 and TRPV6 undergo rapid inactivation in the presence of high intracellular Ca^2+^ concentrations, which allows both proteins to dynamically adjust the Ca^2+^ content of the cell, thereby preventing excessive Ca^2+^ influx and maintaining cellular calcium homeostasis. This inactivation is mainly caused by the binding of Ca^2+^-CaM to the channel protein.^445^^,^^446^ Cryo-EM studies of TRPV5 and TRPV6, together with Ca^2+^-CaM, have revealed that upon binding of Ca^2+^-CaM to the C-terminal regions of the channel, it sterically inhibits the ion-conducting pore.447., 448., 449. The basal activity of both channels is, therefore, largely determined by the interplay between PIP_2_-dependent activation and Ca^2+^-CaM–dependent inactivation.^353^^,^^450^

In electrophysiological recordings, the current voltage curve of TRPV5 and TRPV6 displays a marked inward rectification, which is a hallmark of Ca^2+^ channels and can be partly attributed to the inhibition by intracellular Mg^2+^ via a mechanism that has yet to be clarified.^357^^,^^439^^,^^451^ In conclusion, the biophysical properties of TRPV5 and TRPV6, including their high Ca^2+^ selectivity, constitutive activity, and regulation by CaM and calciotropic hormones, underscore their importance in maintaining cellular and organismic calcium homeostasis.

### C Expression pattern and primary physiological roles of TRPVs

Numerous studies have investigated the mRNA and protein expression of TRPVs in a variety of species using different methodologies, which have sometimes yielded inconsistent findings. This chapter focuses on TRPV expression in human tissues and includes data from the Human Protein Atlas and single-cell transcriptomic analyses.^452^^,^^453^ An overview of the expression profile of TRPVs is provided in Table 1.

The most prominent expression of TRPV1 is found in nociceptive neurons whose somata are localized in the dorsal root ganglia (DRG) and in the trigeminal ganglion.454., 455., 456. The fine nerve endings of their dendrites reach the entire skin, the oropharyngeal mucosa, and other internal organs, such as the urinary bladder. Compared with strongly myelinated sensory neurons that confer touch sensitivity, the TRPV1-expressing nociceptive neurons typically have a small or medium diameter and feature either poorly myelinated A*δ* fibers or unmyelinated C fibers. In the case of DRG neurons, they terminate in the substantia gelatinosa within the dorsal horn of the spinal cord, where they are connected to the second neuron of the pain pathway and the spinothalamic tract via excitatory glutamatergic synapses. The primary afferent function of TRPV1 channels is to confer heat perception, thermal nociception, and the pungent or “hot” sensation of various alimentary spices. In diseased states that trigger inflammation, TRPV1 can become strongly sensitized and chiefly mediates inflammatory thermal hyperalgesia and constant pain sensations.457., 458., 459.

Importantly, TRPV1-expressing nociceptive neurons also exert a pseudo-efferent function by releasing the strongly vasodilatory calcitonin gene-related peptide and the inflammation-mimicking peptide substance P from free nerve endings, which contribute to thermoregulation by enhancing cutaneous blood flow and passive heat dissipation.^424^^,^^460^ Since TRPV1 is activated by warmth or moderate heat, this feedback mechanism is ideally suited to maintain body temperature within a narrow range while not yet losing significant amounts of water and electrolytes, which would be the consequence of sweating. Notably, most TRPV1 inhibitors also disrupt this thermoregulatory function. Consequently, the adverse effects of analgesic TRPV1-targeting drugs not only include burning or scalding injuries, but also a significant elevation of body temperature.^459^^,^^461^^,^^462^ In the brain, TRPV1 expression is found in neurons, astrocytes, and microglia.^463^

TRPV2 has initially been identified in DRG neurons as well, but the TRPV2-positive neurons are larger in diameter and poorly overlap with the population of TRPV1-expressing neurons.^411^ Later, the expression of TRPV2 has been found to be much more widespread, with the strongest expression in various immune cells, including macrophages, monocytes, neutrophils, T lymphocytes, mast cells, and dendritic cells.464., 465., 466. In the CNS, TRPV2 is also strongly expressed in a wide variety of excitatory or inhibitory neurons and in the microglia. According to single-cell transcriptomic analyses, an abundant expression of TRPV2 is found in tissue-resident immune cells, such as lung macrophages, placental Hofbauer cells, as well as nonimmune cells, such as cutaneous melanocytes, vascular smooth muscle cells, the urothelium, and red blood cells.^453^^,^^467^^,^^468^

Despite its high abundance in various cell types, the primary function of TRPV2 is still poorly understood. No obvious thermal or mechanical nociceptive sensory phenotype has been detected in mice lacking TRPV2 expression.^469^ In agreement with the strong TRPV2 expression in cell types of the innate and adaptive immune system, phenotypes are more prominent upon immunological challenges. In macrophages, TRPV2 activity is critical for efficient cell migration, phagocytosis, and bacterial clearance.^470^^,^^471^ Similarly, TRPV2-deficient mice displayed attenuated B-cell responses and antibody formation upon immunization.^472^

The most prominent site of TRPV3 expression is found in basal and suprabasal cutaneous keratinocytes, as well as in epithelial cells of the hair follicles.^425^^,^^473^ TRPV3 expression has also been detected in sensory DRG and trigeminal ganglion neurons,^408^^,^^474^ but based on KO mouse models, the functional role of TRPV3 as a primary sensor for warmth or heat perception has been controversial.^475^^,^^476^ Since TRPV3, like TRPV4, contributes to warmth-induced ionic currents in keratinocytes, a functional link to sensory neurons may involve the formation or release of paracrine factors such as ATP, prostaglandin E_2_, nitric oxide, or transforming growth factor-*α* to transmit the signals to sensory neurons.^393^^,^477., 478., 479., 480., 481. TRPV3 expression has also been demonstrated in epithelial tissues of the oral cavity, in glandular cells of the small intestine, and in enterocytes of the small and large intestine.^453^^,^^482^^,^^483^ In the CNS, a moderate TRPV3 expression is found dispersed over neuronal and glial cells.

The primary function of TRPV3 is best established in the development and maintenance of intact skin architecture. As GOF mutations in TRPV3 cause hair loss and mutilating keratoderma (see below), and since TRPV3-deficient mice display wavy hairs, curly whiskers, and a partially defective skin barrier,^393^^,^^473^ undisturbed TRPV3 activity appears indispensable for the proper development of the skin and skin appendages. Consistently, TRPV3 activity has been shown to promote keratinocyte proliferation and migration in vitro, and may therefore support wound healing.^482^^,^^484^^,^^485^

Among the heat-sensitive TRPV1–4 channels, the expression of TRPV4 is most widespread. Initially, its expression has been detected in the kidney, lung, trachea, liver, spleen, brain, prostate, and placenta.486., 487., 488., 489. At the cellular level, TRPV4 is strongly expressed in many human epithelial, glandular, and endothelial cell types, such as in exocrine epithelial cells of the salivary and pancreatic glands, in tracheal, bronchial, and fallopian tube ciliated epithelial cells, in epithelial cells of the choroid plexus, in tubular epithelia of the kidney, in female breasts, in tracheal and tongue glandular cells, in placental trophoblast and decidual cells, in vascular endothelial cells, and in skin keratinocytes and melanocytes, only to name a few.^452^^,^^453^ High levels of TRPV4 expression have also been found in tissue-resident macrophages, including hepatic Kupffer cells.^453^

In line with the widely distributed expression of TRPV4, manifold primary functions of TRPV4 have been identified. A common motif of some of them is based on the indirect activation of TRPV4 by hypotonic stress, causing the conversion of arachidonic acid to epoxyeicosatrienoic acids that, in turn, activate TRPV4.^490^^,^^491^ In glandular and exocrine cells, the activation of Ca^2+^ influx through TRPV4 seems to initiate a secondary opening of anoctamin 1, a Ca^2+^-regulated chloride channel, to initiate fluid secretion,^492^^,^^493^ while acute pharmacological activation of TRPV4 in vascular endothelial cells mediates the formation of nitric oxide and triggers microvascular leakage, causing circulatory collapse.^494^ Under more physiological conditions, shear stress can activate endothelial TRPV4 channels, thereby triggering vasodilation and outgrowth of collateral vessels.^24^^,^^495^^,^^496^ Finally, TRPV4 plays an important role in development, as pathogenic GOF mutations in human TRPV4 are linked to congenital skeletal and neuromuscular disorders.^497^

TRPV5 is mainly expressed in the apical membrane compartment of epithelial cells of the kidney, distal convoluted tubules (DCTs), and collecting ducts.^441^ In human tissues, TRPV5 transcripts have also been detected in the pancreas, duodenum, jejunum, colon, placenta, prostate gland, testis, brain, and bone osteoclasts.^498^^,^^499^ Vitamin D-response elements have been identified in the TRPV5 promoter, and TRPV5 protein expression was found to correlate with the expression of other vitamin D receptor target genes in rat kidneys.^500^ Other studies found that TRPV5 expression in mice appeared to be regulated^501^ by Ca^2+^ rather than by 1,25-dihydroxyvitamin D_3_ or that TRPV5 expression is also regulated^502^ by estrogens. In DCTs obtained from the kidneys of transgenic reporter mice that express enhanced GFP under the control of a TRPV6 promoter, 1,25-dihydroxyvitamin D_3_- and parathyroid hormone-dependent transcriptional regulation of TRPV5 was detected.^503^ In the same study, TRPV5 deficiency was shown to strongly impede transepithelial Ca^2+^ transport, which also represents the primary function of TRPV5. TRPV5 deficiency is associated with severe renal Ca^2+^ wasting, highlighting the seminal role of TRPV5 in renal Ca^2+^ reabsorption and bone mineralization.^504^^,^^505^

Compared with TRPV5, the expression of TRPV6 in mice is more widespread and mostly found in extrarenal tissues and organs. It includes the Ca^2+^-absorbing epithelia in the small intestine, exocrine and endocrine epithelia of the salivary gland, pancreas, and prostate gland, as well as subsets of epithelial cells in the thyroid, stomach, duodenum, caecum, epididymis, endometrium, placenta, and mucus-secreting epithelia in the main olfactory epithelium and the bronchiae.506., 507., 508. In human tissues, a similar TRPV6 expression pattern has been found.^453^^,^509., 510., 511. Like TRPV5, TRPV6 expression is regulated in a 1,25-dihydroxyvitamin D_3_-dependent fashion.^512^^,^^513^ In addition, TRPV6 expression has been shown^501^^,^^514^^,^^515^ to be upregulated by estrogens and dietary Ca^2+^.

Notably, TRPV6 expression in polarized epithelia strongly overlaps with that of the vitamin D receptor and other 1,25-dihydroxyvitamin D_3_-regulated proteins that are involved in transepithelial Ca^2+^ transport, such as the Ca^2+^-buffering calbindins D(9k) and D(28k), as well as the plasma membrane calcium ATPase.^510^^,^^516^ Accordingly, the primary function of TRPV6 is to transport Ca^2+^ across epithelial barriers. Important transport routes include the 1,25-dihydroxyvitamin D_3_-dependent regulation of Ca^2+^ resorption in the small intestine,^517^ fetal Ca^2+^ supply via placental Ca^2+^ transport,^518^ and maintenance of fertility by lowering the Ca^2+^ concentration in the seminal fluid.^519^

### D Human diseases associated with TRPVs

Although variants in the TRPV1 gene have been identified, they are rare and not commonly associated with human diseases. Katz et al^520^ reported the phenotypes of 2 individuals carrying a homozygous missense mutation in the ARD of the channel. This mutation, which leads to a complete loss of TRPV1 activity, causes an elevated heat-pain tolerance and a higher cold-pain threshold. Another study linked 2 independently identified TRPV1 missense variants in individuals to a high risk of malignant hyperthermia.^521^ Other SNPs in TRPV1 were associated with nocturnal, usual, and chronic cough.^522^

An altered TRPV2 expression is mainly associated with the development and progression of several solid tumors and hematological malignancies, as reviewed recently.^466^^,^^523^ For instance, in triple-negative breast cancer (TNBC), TRPV2 expression correlates with recurrence-free survival of TNBC patients, opening up the possibility that TRPV2 activation, for example, by cannabidiol, might be beneficial as an adjuvant therapy in TNBC.^524^ A similar observation was made in patients suffering from glioblastoma, where TRPV2 expression decreased with disease progression.^525^ In contrast, a higher TRPV2 expression was associated with worse outcomes in multiple myeloma,^526^ prostate cancer,^527^ and gastric carcinoma.^528^

Pathologies arising from TRPV3 dysfunction mainly affect the skin and are strongly associated with itch. The clearest link exists between Olmsted syndrome (OLMS1, OMIM 614594), a rare congenital disorder, and GOF mutations in TRPV3, as demonstrated in a series of clinical reports.^473^ Olmsted syndrome is characterized by palmoplantar keratoderma and periorificial hyperkeratosis, accompanied by severe pruritus and, in extreme cases, spontaneous amputation of fingers or toes. An elevated expression of TRPV3 is also linked to atopic dermatitis^529^^,^^530^ and psoriasis,^531^ inflammatory skin conditions, in which TRPV3 activation might contribute to chronic pruritus. Furthermore, patients with itching scars from burn injuries display increased TRPV3 expression in the epidermis of the affected areas. The role of TRPV3 in pruritus is further highlighted by the fact that topical application of the TRPV3 activator carvacrol causes itching in burn scars.^532^^,^^533^

Autosomal dominant TRPV4 disorders are primarily associated with skeletal dysplasias or motor function disorders, though phenotypic overlap occurs. In skeletal dysplasia, affected individuals mainly present with brachydactyly, short stature, and progressive scoliosis,^534^ but individual manifestations and severity vary among individuals. More than 50 different TRPV4 mutations have been identified so far,^535^ distributed widely across the gene with a clustering of mutations in the region between TM5 and TM6. Most mutations lead to overactive TRPV4 channels, as seen in autosomal dominant brachyolmia type 3 (OMIM 113500),^536^ metatropic dysplasia (OMIM 156530),^537^ and spondylometaphyseal Kozlowski type dysplasia (OMIM 1842522).^538^ However, some reported mutations also result in a reduced availability of TRPV4 at the plasma membrane, for example, in familiar digital arthropathy-brachydactyly (OMIM 606835).^539^ A TRPV4 mutation with a trafficking defect has also been observed in hereditary motor and sensory neuropathy type IIC (OMIM 606071), also known as Charcot-Marie-Tooth disease type 2C, a neuromuscular disorder mainly characterized by progressive peripheral neuropathy, as well as in congenital distal spinal muscular atrophy (OMIM 600175) and scapuloperoneal spinal muscular atrophy (OMIM 606071).^540^^,^^541^

Recently, a pathogenic homozygous missense mutation in TRPV5 (V598M) was identified that causes a LOF phenotype associated with a novel form of autosomal recessive hypercalciuria and calcium wasting. The mutation, which affects the TRP helix region, results in protein misfolding and a complete loss of TRPV5-mediated calcium uptake upon overexpression in human embryonic kidney (HEK) 293 cells.^505^

Dysregulation of TRPV6 activity by mutations or abnormal expression levels is linked to several human diseases. Homozygous or compound heterozygous mutations in TRPV6 have been identified in individuals suffering from transient neonatal hyperparathyroidism (OMIM 618188), a condition associated with fetal skeletal abnormalities. Some of the mutations cause TRPV6 trafficking deficits or partial loss of function, which is believed to reduce calcium transport across the placenta, followed by an impaired fetal bone mineralization.^542^^,^^543^ Functionally deficient TRPV6 variants are also associated with hereditary and familial pancreatitis.^544^^,^^545^ Moreover, in recent years, several studies have attributed TRPV6 as an oncochannel in cancers of epithelial origin.^546^ In most malignancies, an elevated TRPV6 expression correlates with a more aggressive form of the disease and a higher risk for metastasis, possibly contributing to a poorer prognosis in prostate cancer,547., 548., 549. breast cancer,^550^^,^^551^ ovarian cancer,^552^ and pancreatic cancer.^553^ However, additional research is needed to fully understand the mechanisms by which the putative oncochannels TRPV6 and TRPV2 may influence cancer progression and to explore the potential of pharmacological modulation – whether activation or inhibition – as a therapeutic strategy for controlling tumor growth and metastasis in specific cancer types.

### E Pharmacological modulators of TRPVs

Apart from TRPV1 and, to a lesser extent, TRPV4, the availability of specific and potent TRPV modulators remains limited. While currently available modulators provide valuable tools, their limitations regarding specificity, potency, and toxicity when applied in vivo underscore the need for the development of novel compounds. Table 3^453^^,^^454^^,^^471^^,^^484^^,^554., 555., 556., 557., 558., 559., 560., 561., 562., 563., 564., 565., 566., 567., 568., 569., 570., 571., 572., 573., 574., 575., 576., 577., 578., 579., 580., 581., 582., 583., 584., 585., 586., 587., 588., 589., 590., 591., 592., 593., 594., 595., 596., 597., 598., 599., 600., 601., 602. provides an overview of TRPV modulators.

**Table 3:** Pharmacological modulators of TRPVs.

Name (PubChem CID^a^)	Effect	References
**TRPV1**		

**Selected TRPV1 inhibitors**		

SB-705498 (9910486)	Inhibition hTRPV1 IC_50_ = 3–6 nM; in clinical trials	^554^
AMG 517 (16007367)	Inhibition hTRPV1 IC_50_ = 0.9 nM; in clinical trials	^555^
A-1165442 (46191567)	Inhibition hTRPV1 IC_50_ = 9 nM for capsaicin activation, partial block of H^+^ activation	^556^
A-1165901 (171378652)	Inhibition hTRPV1 IC_50_ = 19 nM for capsaicin activation, potentiation of H^+^ activation	^557^
AMG8562 (56603667)	Inhibition hTRPV1 IC_50_ = 1.8 nM for capsaicin activation; IC_50_ > 10 *μ*M for heat activation, potentiates H^+^ activation	^558^
NEO6860 (N/A)	Inhibition hTRPV1 IC_50_ = 41.5 nM for capsaicin activation; IC_50_ > 4 *μ*M for heat activation; in clinical trials	^559^
PAC-14028 (asivatrep) (56649347)	Inhibition rTRPV1 IC_50_ = 55 nM; topical application; in clinical trials	^560^

**Selected TRPV1 activators**		

Capsaicin (1548943)	Activation rTRPV1 EC_50_ = 0.7 *μ*M; hTRPV1 EC_50_ = 31.6 nM; in clinical trials	^454^^,^^561^
Resiniferatoxin (5702546)	Activation rTRPV1 EC_50_ = 39.1 nM; hTRPV1 EC_50_ = 4 nM; in clinical trials	^454^^,^^561^
Anandamide (5281969)	Activation hTRPV1 EC_50_ = 1.3 *μ*M	^562^
CA-008 (vocacapsaicin) (121349852)	Prodrug of trans-capsaicin; in clinical trials	^563^

**TRPV2**		

**Inhibitors**		

Tranilast (5282230)	Inhibition hTRPV2, mTRPV2 IC_50_ approx. 10 *μ*M; in clinical trials	^564^
Lumin (23305342)	Inhibition mTRPV2 IC_50_ = 5 *μ*M	^564^
Valdecoxib (119607)	Inhibition rTRPV2 IC_50_ = 10 *μ*M	^565^
Monanchomycalin B (102489008)	Inhibition mTRPV2 IC_50_ = 2.8 *μ*M; hTRPV3 IC_50_ = 3.2 *μ*M	^566^
B304-1 (N/A)	Partial inhibition mTRPV2 IC_50_ = 22.2 *μ*M	^567^
B304-2 (N/A)	Partial Inhibition mTRPV2 IC_50_ = 3.7 *μ*M	^567^
Piperlongumine (637858)	Inhibition hTRPV2 IC_50_ = 4.6 *μ*M	^568^
SET2 (155541857)	Inhibition mTRPV2 IC_50_ = 0.5 *μ*M	^569^
IV2-1 (N/A)	Inhibition rTRPV2 IC_50_ = 6.3 *μ*M	^471^

**Activators**		

Probenecid (4911)	Activation; in clinical trials	^570^
Cannabidiol (644019)	Activation	^571^

**TRPV3**		

**Inhibitors**		

Citrusinine-II (10016895)	Inhibition mTRPV3 IC_50_ = 12.4 *μ*M	^572^
Isochlorogenic acid A (6474310)	Inhibition hTRPV3 IC_50_ = 2.7 *μ*M	^573^
Isochlorogenic acid B (5281780)	Inhibition hTRPV3 IC_50_ = 0.9 *μ*M	^573^
Osthole (10228)	Inhibition hTRPV3 IC_50_ = 37 *μ*M	^574^
Forsythoside B (23928102)	Inhibition hTRPV3 IC_50_ = 6.7 *μ*M	^575^
Verbascoside (5281800)	Inhibition hTRPV3 IC_50_ = 14 *μ*M	^576^
Alpha-mangostin (5281650)	Inhibition hTRPV2 IC_50_ = 77 nM; hTRPV2 GOF mutant IC_50_ approx. 2 *μ*M	^577^
Compound 74a (155184122)	Inhibition hTRPV3 IC_50_ = 0.38 *μ*M	^578^
Trpvicin (122589101)	Inhibition hTRPV3 IC_50_ = 0.38 *μ*M; blocks G573S GOF mutant IC_50_ = 0.66 *μ*M	^579^
Local anesthetics (bupivacaine, mepivacaine, lidocaine, ropivacaine)	Inhibition hTRPV3 low potency (0.17–2 mM)	^580^
Dyclonine (3180)	Inhibition mTRPV3 IC_50_ = 3.2 *μ*M	^581^
Flopropione (3362)	Inhibition hTRPV3 IC_50_ = 18 *μ*M	^582^
GRC15300 (N/A)	Inhibition of TRPV3; in clinical trials	Reviewed in^583^

**Activators**		

Naturally occurring monoterpenes (thymol, carvacrol, camphor)	Activation of low potency	^584^
Incensole acetate (73755086)	Activation mTRPV3 EC_50_ = 16 *μ*M	^585^
Tetrahydrocannabivarin (93147)	Activation rTRPV3 EC_50_ = 6.1 *μ*M	^586^
KS0365 (N/A)	Activation mTRPV3 EC_50_ = 5.1 *μ*M (cholesterol-enriched cells)	^484^

**TRPV4**		

**Inhibitors**		

GSK2193874 (53464483)	Inhibition rTRPV4 IC_50_ = 2 nM; hTRPV4 IC_50_ = 40 nM	^587^
GSK2798745 (71227359)	Inhibition hTRPV4 IC_50_ = 1.8 nM; in clinical trials	^588^
HC-067047 (2742550)	Inhibition hTRPV4 IC_50_ = 48 nM; rTRPV4 IC_50_ = 133 nM; mTRPV4 IC_50_ = 17 nM	^589^
RN-1734 (3601086)	Inhibition hTRPV4 IC_50_ = 2.3 *μ*M	^590^
RN-9893 (121513880)	Inhibition hTRPV4 IC_50_ = 0.42 *μ*M; rTRPV4 IC_50_ = 0.66 *μ*M; mTRPV4 IC_50_ =0.32 *μ*M	^591^

**Activators**		

GSK1016790A (23630211)	Activation hTRPV4 EC_50_ = 2 nM; mTRPV4 EC_50_=2.1 nM	^592^
36-HCl (N/A)	Activation hTRPV4 EC_50_ = 60 nM	^593^
RN-1747 (5068295)	Activation hTRPV4 EC_50_ = 0.77 *μ*M; m/rTRPV4 EC_50_ = 4 μM	^590^
Curcumin (969516)	Activation is low potency, poor selectivity	^594^
Puerarin (5281807)	Activation is low potency	^595^

**TRPV5,6**		

**Inhibitors**		

Miconazole (4189)	Inhibition, TRPV6 > TRPV5; active >100 *μ*M	^596^
Econazole (3198)	Inhibition, TRPV6 > TRPV5; active >100 *μ*M	^596^
ZINC17988990 (27791261)	Inhibition rbTRPV5 IC_50_ = 0.11 *μ*M; hTRPV5 IC_50_ = 0.18 *μ*M	^597^
Compound 3 (N/A)	Inhibition TRPV6 IC_50_ = 90 *μ*M; TRPV5 IC_50_ = 503 *μ*M	^596^
*cis-*22a (169553405)	Inhibition hTRPV6 IC_50_ = 0.32 *μ*M	^598^
3OG (N/A)	Inhibition hTRPV6 IC_50_ = 83 nM; hTRPV5 IC_50_ = 531 nM	^599^
SOR-C13 (121596688)	Inhibition hTRPV6 IC_50_ = 14 nM; in clinical trials	^600^
SOR-C27 (N/A)	Inhibition TRPV6 IC_50_ = 64 nM	^600^
Tetrahydrocannabivarin (93147)	Inhibition rTRPV5 IC_50_= 4.8 *μ*M; mTRPV6 IC_50_ = 9.4 *μ*M	^601^
Compound 9e (N/A)	Photoswitchable inhibitor TRPV6	^602^

a PubChem Compound Identification number. N/A – not available.

#### 1 TRPV1

Over the past 20 years, numerous TRPV1-modulating compounds, inhibitors, and activators have been introduced, primarily with the intention of treating diverse pain conditions. As a highly druggable target, TRPV1 has attracted considerable research interest, resulting in an abundance of selective and potent modulators. They will be only briefly summarized here, and we refer to current reviews for more detailed information.^603^

First-generation TRPV1 inhibitors, such as SB-705498 or AMG 517,^554^^,^^555^ are polymodal antagonists of TRPV1 that block activation by capsaicin, protons, and heat, as reviewed by Garami et al.^462^ However, many of these compounds cause hyperthermia in vivo and reduce the perception of noxious heat, resulting in burn injuries (reviewed by Romanovsky et al^461^). To address these issues, second-generation TRPV1 inhibitors were developed, which target TRPV1 depending on the mode of channel activation. For instance, A-1165442 blocks capsaicin and heat-evoked TRPV1 responses with minimal effects on H^+^-activated TRPV1 and does not significantly change core body temperature in rats.^556^ Other drugs, such as A-1165901 and AMG8562,^557^^,^^558^ block TRPV1 activation by capsaicin while potentiating H^+^ activation. Interestingly, they cause hypothermia in mice. NEO6860 is specific only for vanilloid activation of TRPV1 and leaves H^+^ activation unaffected.^559^ It also does not alter body temperature.^604^

The prototypical TRPV1 activator is capsaicin.^453^ Other naturally occurring TRPV1 activators include the superagonist resiniferatoxin,^561^ arachidonic acid metabolites such as anandamide,^562^ and several venom peptides, as reviewed by Hwang et al.^605^ Capsaicin is therapeutically relevant in the treatment of neuropathic pain conditions due to its ability to desensitize and ultimately cause ablation and defunctionalization of TRPV1-expressing pain-conducting fibers after prolonged application, such as through topical capsaicin patches, as recently reviewed by Alalami et al.^606^

#### 2 TRPV2

Compared with TRPV1, the development of TRPV2-modulating compounds has been much less the focus of research. While specific and potent activators of TRPV2 are still lacking, some progress has been made regarding TRPV2 inhibitors. Iwata et al^564^ identified several TRPV2 inhibitors, including the antiallergic drug tranilast (IC_50_ = 10 *μ*M) and the cyanine dye lumin (IC_50_ = 5 *μ*M). Lumin acts as a general immunostimulant and exerts cardioprotective effects in a hamster model of dilated cardiomyopathy (*δ*-sarcoglycan-deficient hamster).^564^ The administration of tranilast prevents cardiac dysfunction in a mouse cardiomyopathy model (dystrophin-utrophin double KO)^607^ and suppresses fibrosis progression in a mouse model of nonalcoholic steatohepatitis.^608^ However, apart from its action on TRPV2, tranilast also exerts pleiotropic effects on other targets in immune cells, fibroblasts, the cardiovascular system, and tumor cells, as reviewed by Darakhshan and Pour,^609^ and it needs to be further confirmed to what extent the beneficial effects of tranilast and lumin in cardiac disease models depend on TRPV2 inhibition. Valdecoxib, a cyclooxygenase-2 inhibitor withdrawn from the market due to its unfavorable cardiovascular side effects, blocks rat TRPV2 channels with moderate potency (IC_50_ = 10 *μ*M) but not TRPV1, TRPV3, and TRPV4 channels.^565^ Monanchomycalin B, an alkaloid isolated from the marine sponge *Monanchora pulchra,* only poorly discriminates between TRPV1, TRPV2, and TRPV3 channels (IC_50_ = 6.0, 2.8, and 3.2 *μ*M, respectively).^566^ Other natural compounds that inhibit TRPV2 include coumarin derivative enantiomers from the roots of the orange jasmine *Murraya exotica*, B304-1 and B304-2, which partially inhibit^567^ TRPV2 channels (IC_50_ = 22.2 and 3.7 *μ*M, respectively) but not TRPV1, TRPV3, or TRPV4 channels, as well as piperlongumine, an alkaloid from the long pepper *Piper longum*. Piperlongumine selectively inhibits human TRPV2 (IC_50_ = 4.6 *μ*M) and reduces tumor sizes when applied to a murine glioblastoma model.^568^ However, due to low solubility, the compound has to be encapsulated in *β*-cyclodextrin and applied to an implantable dextran-dendrimer hydrogel scaffold.

Synthetic TRPV2 inhibitors include SET2 (IC_50_ = 0.5 *μ*M)^569^ and IV2-1 (IC_50_ = 6.3 *μ*M),^471^ which do not affect TRPV1, TRPV3, and TRPV4 channels. However, neither compound has been tested in TRPV2-relevant disease models yet.

Regarding TRPV2 activation, particularly human TRPV2, has proven difficult to activate without inducing cytotoxic effects at the concentrations of the drugs required for robust activation. Currently, probenecid and cannabinoids, or a combination of both, are primarily used for in vitro studies.^412^^,^^570^^,^^571^^,^^610^

#### 3 TRPV3

Various natural compounds isolated from plants inhibit TRPV3 channels, though most of them are only moderately potent. Nonetheless, some of them have demonstrated efficacy in vivo, particularly in mouse models of acute and chronic itch. Citrusinine II, derived from the small evergreen tree *Atalantia monophylla,* inhibits TRPV3, albeit with a relatively low potency (IC_50_ = 12.4 *μ*M). It suppresses itch in mouse models of both acute and chronic pruritus when administered subcutaneously.^572^ Naturally occurring isochlorogenic acid A (IC_50_ = 2.7 *μ*M) and B (IC_50_ = 0.9 *μ*M), active ingredients of the herb *Achillea alpina*, inhibit TRPV3 and reduce ear swelling and chronic pruritus in mouse models of topical carvacrol treatment.^573^^,^^611^ The coumarin osthole, isolated from *Cnidium monnieri* (IC_50_ = 37 *μ*M for hTRPV3)—a plant used in traditional Chinese medicine—attenuates dry skin itch and histamine-dependent itch.^574^ Subsequent studies by the same group have demonstrated the efficacy of the TRPV3 inhibitors forsythoside B, which is found in a number of plants of the mint order (IC_50_ = 6.7 *μ*M),^575^ and plant-derived verbascoside (IC_50_ = 14 *μ*M) in similar disease models.^576^ More recently, *α*-mangostin from the mangosteen plant was identified as a highly potent inhibitor of WT TRPV3 (IC_50_ = 77 nM) and TRPV3 GOF mutants (G573S and G573C) (IC_50_ ∼2 *μ*M).^577^

In addition to naturally occurring substances, several chemically synthesized TRPV3 inhibitors were developed. Optimization of primary hits regarding absorption, distribution, metabolism, and excretion properties has led to the discovery of compound 74a (IC_50_ = 0.38 *μ*M) with favorable drug-like properties and efficacy in mouse models of neuropathic and central pain.^578^ Another compound, Trpvicin (IC_50_ = 0.38 *μ*M), stabilizes both WT TRPV3 and a GOF mutant (G573S) in their closed conformations, effectively inhibiting hair loss in a mouse model carrying the G568V mutation, relieving symptoms of chronic and acute itch.^579^ Some local anesthetics, which are sometimes used to treat pruritus and pain, have also shown efficacy in inhibiting TRPV3 channels, although with low potency. Bupivacaine, mepivacaine, lidocaine, and ropivacaine inhibit TRPV3 with IC_50_ values ranging from 170 *μ*M to 2.5 mM.^580^ Dyclonine, a clinically used anesthetic, acts at least 2 orders of magnitude more potently on TRPV3 channels (IC_50_ = 3.2 *μ*M) than on TRPV1, TRPV2, TRPM8, and TRPA1 and relieves carvacrol-induced scratching in mice.^581^ Flopropione, an antispasmic agent, also blocks TRPV3 channels (IC_50_ = 18 *μ*M) and alleviates symptoms in mouse models of skin inflammation induced by skin sensitizers.^582^

Naturally occurring monoterpenes, such as thymol, carvacrol, or camphor, activate TRPV3 but with poor potencies.^584^ A screening of Boswellia extracts for bioactive components identified the diterpene incensole acetate (EC_50_ = 16 *μ*M) as a novel TRPV3 activator. It exerts antidepressant and anxiolytic effects in WT but not in TRPV3-deficient mice, suggesting that these effects are indeed mediated via TRPV3 activation.^585^ Tetrahydrocannabivarin, a nonpsychoactive analogue of tetrahydrocannabinol, stimulates TRPV3 channels (EC_50_ = 6.1 *μ*M) by binding to the vanilloid site but also activates several other TRP channels.^376^^,^^586^ More recently, the synthetic compound KS0365 was identified, showing 3-fold greater potency than 2-aminoethoxydiphenyl borate (EC_50_ = 5.1 *μ*M, calculated in cholesterol-enriched cells) in activating TRPV3 without affecting TRPV2 channels.^484^

#### 4 TRPV4

Significant progress has been made to improve the pharmacology of TRPV4 channels, as several pharmaceutical companies have set out to develop novel TRPV4 modulators. Subsequently, their efficacies have been demonstrated in different mouse models of diseases.

In terms of antagonists, highly potent and selective drugs are now available. Thorneloe et al^587^ reported that the orally available TRPV4 antagonist GSK2193874 (IC_50_ = 2 nM for rTRPV4 and 40 nM for hTRPV4) was beneficial in mouse models of pulmonary edema. GSK2193874 is highly specific for TRPV4, demonstrating selectivity across more than 200 tested targets. The compound GSK2798745 (IC_50_ = 1.8 nM) resulted from a lead optimization process and demonstrated efficacy in a rat model of pulmonary edema.^588^ In rats, cyclophosphamide-induced cystitis was inhibited by Hydra's HC-067047 (IC_50_ values were 48 nM for hTRPV4, 133 nM for rTRPV4, and 17 nM for mTRPV4).^589^ Renovis Pharma also identified several TRPV4-targeting modulators, including both activators and inhibitors. RN-1734 inhibited TRPV4 (IC_50_ = 2.3 *μ*M) with moderate potency.^590^ Later, the same group^591^ introduced orally bioavailable RN-9893 with an improved potency (IC_50_ values of 0.42 *μ*M, 0.66 *μ*M, and 0.32 *μ*M for human, rat, and mouse TRPV4 receptors, respectively) and high specificity for TRPV4.

Several selective activators of TRPV4 are available. GlaxoSmithKline’s GSK1016790A is highly potent (EC_50_ = 2 nM for hTRPV4) and selective for TRPV4. Systemic administration of GSK1016790A in animals causes a severe drop in blood pressure up to circulatory collapse and death, highlighting the role of TRPV4 in the regulation of vascular tone and vasodilation.^494^^,^^592^ Recently, a novel TRPV4 agonist was discovered (EC_50_ = 60 nM), which is suitable for in vivo application. The quinazolin-4(3H)-one derivative 36-HCl suppressed the progression of osteoarthritis in a rat model of surgically induced osteoarthritis (meniscal tear model) through intra-articular application.^593^ Renovis Pharma introduced the piperazine RN-1747 with EC_50_ values of 0.77 and 4 *μ*M for hTRPV4 and mTRPV4/rTRPV4, respectively.^590^

Naturally occurring TRPV4 activators include curcumin and puerarin, although both compounds only show low potencies and are, in the case of curcumin, only poorly selective for TRPV4.^594^^,^^595^

#### 5 TRPV5 and TRPV6 channels

Several compounds block TRPV5 and TRPV6 channels. Initially, their potency was low, and most of them did not discriminate well between the 2 isoforms. Miconazole and econazole demonstrate approximately 2-fold higher activity for TRPV6 than for TRPV5 but require high concentrations (>100 *μ*M) for effective channel blockade.^596^ Cryo-EM studies of TRPV6 in complex with econazole revealed binding to the periphery of the channel, where econazole replaced a lipid.^612^^,^^613^

Structure-based virtual screening has further advanced the identification of TRPV5-selective compounds. By virtually screening the econazole binding pocket using a database of 12 million compounds, 3 novel TRPV5 inhibitors were identified, including ZINC17988990, which selectively inhibits rabbit human TRPV5 but not human TRPV6 (IC_50_ = 0.11 and 0.18 *μ*M, respectively).^597^

Based on the lead compound TH-1177,^614^ Landowski et al^596^ introduced the weakly potent compound 3 with a 5-fold selectivity for TRPV6 (IC_50_ = 90 *μ*M) over TRPV5 (IC_50_ = 503 *μ*M). Subsequent efforts have led to the development of *cis-*22a (IC_50_ = 0.32 *μ*M) through ligand-based virtual screening, which exerts a 7-fold selectivity for TRPV5 compared with TRPV6.^598^ However, *cis-*22a is not suitable for in vivo studies due to its low stability against microsomal degradation. Chemical modification of *cis-*22a resulted in the discovery of 3OG with a higher potency for TRPV6 inhibition (IC_50_ = 83 nM) and improved microsomal stability.^599^ Cryo-EM, X-ray crystallography, and mutagenesis studies identified 2 types of binding sites for *cis-*22a in the TM region: one overlaps with lipid binding site 2 and the other is located at the intracellular pore entry site, which also serves as a binding region for Ca^2+^-CaM.^615^^,^^616^

Several naturally occurring compounds inhibit TRPV6 channels. SOR-C13 and SOR-C27, 2 short peptides derived from sorcidin, a paralytic venom of the shrew *Blarina brevicauda*, block TRPV6 with IC_50_ values of 14 and 64 nM, respectively. In mice, these peptides were used to detect TRPV6-overexpressing tumors^600^ and reduced tumor growth in a xenograft model.^552^ Tetrahydrocannabivarin blocks both TRPV5 and TRPV6 channels (IC_50_ = 4.8 *μ*M and 9.4 *μ*M, respectively) by binding to a site at the interface between the channel’s pore and the surrounding membrane.^601^^,^^617^

#### 6 Photoswitchable inhibitors of TRPVs

Recently, Cunha et al^602^ developed a photoswitchable TRPV6 inhibitor based on the chemical structure of a previously reported TRPV6 inhibitor by introducing a phenyldiazo group to the molecule. Compound 9e rapidly switches by illumination with UVA light from the almost ineffective E-isomer to the inhibitory Z-isomer (IC_50_ = 1.7 *μ*M).^598^^,^^602^

### F Ongoing or completed clinical trials with TRPVs as therapeutic targets

With respect to clinical trials, TRPV1 is by far the most intensely studied member of the TRPV family. According to the ClinicalTrials.gov database, nearly 100 studies have targeted TRPV1 for various conditions, with a focus on asthma and cough, inflammatory skin diseases, and, in particular, various pain conditions. However, due to hyperthermia and an increased likelihood of burn injuries associated with TRPV1 inhibition, many first-generation TRPV1 antagonists were withdrawn from clinical trials or did not progress further.^603^ Mode-specific second-generation TRPV1 inhibitors, such as NEO6860 (NCT02337543), do not affect heat and pH activation of TRPV1 and provide a better safety profile.^559^ However, NEO6860 did not demonstrate superior efficacy compared with placebo in a phase 2 trial to treat knee osteoarthritis (NCT02712957).^604^ Topical TRPV1 antagonists are well tolerated and are under investigation for the treatment of inflammatory skin diseases, such as atopic dermatitis (PAC-14028, asivatrep; NCT02583022, NCT02757729, and NCT02965118), where they show promising effects.^618^ Another approach involves the desensitization of TRPV1 channels, which is used in therapeutic approaches such as the use of capsaicin-containing creams for the treatment of moderate pain or the intravesical instillation of capsaicin or resiniferatoxin for an overactive bladder. This strategy is also being explored in trials investigating capsaicin formulations or the TRPV1 agonist CA-008 (vocacapsaicin) for the management of chronic pain conditions, as reviewed by Iftinca et al.^619^

Compared with TRPV1, far fewer studies have evaluated the efficacy of compounds targeting other members of the TRPV family. Two drug repurposing studies have evaluated the use of the nonspecific TRPV2 activator probenecid, an FDA-approved drug to treat gout and hyperuricemia. In a small phase 4 study (NCT03965351) involving patients with functionally univentricular (Fontan) circulation, probenecid improved cardiac function compared with placebo.^620^ Another phase 2 study, involving 20 patients, investigated probenecid as a positive inotropic agent for the treatment of heart failure (NCT01814319) and demonstrated a better cardiac function.^621^ However, given the nonspecific action of probenecid, further confirmation is required to determine whether TRPV2 activation underpins its potential cardiac benefits. The TRPV2 inhibitor tranilast is currently being studied in a phase 1/2 study of patients suffering from advanced esophageal cancer for its efficacy when combined with traditional chemotherapy (jRCTs051190076).^622^

Although preclinical studies suggest a role for TRPV3 in skin diseases, to date, only the TRPV3 inhibitor, GRC15300 (SAR292833), by Glenmark Pharmaceuticals has progressed to clinical trials for targeting osteoarthritis and neuropathic pain. However, the drug failed to meet its primary endpoint in a phase 2 trial in 2013 (NCT01463397).

Alongside TRPV1, TRPV4-selective modulators have achieved notable clinical progress within the TRPV family. GSK2798745, a highly potent TRPV4 inhibitor developed by GlaxoSmithKline,^588^ has entered several early-phase clinical trials. No safety issues or serious side effects were observed in a phase 1 study (NCT02119260).^623^ However, in 2017, a phase 2a study of heart failure patients (NCT02497937) failed to demonstrate significant effects of TRPV4 inhibition on pulmonary gas diffusion as an indicator of lung congestion.^624^ In 2019, a combined phase 1/2 study (NCT03372603) assessed the effect of the same molecule on chronic cough, but the study was terminated due to a lack of efficacy. GSK2798745 was also unable to reduce alveolar barrier disruption in a model of lipopolysaccharide-induced acute lung injury in another phase 1 trial (NCT03511105),^625^ and the study was terminated due to a low probability of achieving a positive outcome of the primary endpoint. A recently completed phase 1 study (NCT04292912), evaluating GSK2798745 in patients with diabetic macular edema, has yet to publish results. Additionally, in 2023, an observational study started, monitoring the natural history of neuropathic pain in patients with confirmed genetic mutations in the TRPV4 gene (NCT05600764).

TRPV6, due to its overexpression in many solid tumors, is considered to comprise a novel target for anticancer therapy.^600^ In 2015, the safety and tolerability of SOR-C13 from Sorcimed Biopharma were demonstrated in a phase 1 study (NCT01578564) involving patients with advanced solid tumors, with some experiencing antitumor effects of the drug.^626^ These findings were followed up in another recently completed phase 1 trial (NCT03784677), but no results have been published yet. The FDA has granted SOR-C13 an orphan drug designation for advanced ovarian and pancreatic cancer. CBP-1008, by Coherent Biopharma, is a bispecific ligand-drug conjugate targeting folate receptor *α* and TRPV6 linked to the cytostatic monomethyl auristatin E. Treatment with CBP-1008 is currently evaluated in an ongoing phase 1 trial (NCT04740398) for advanced solid tumors.

## IV TRPMs

### A TRPM gene family

**Fig. 4:**
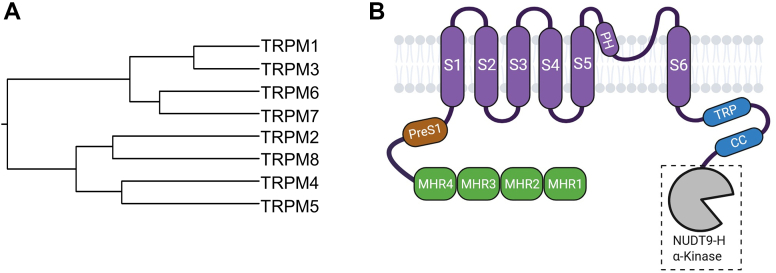
The relatedness and domain topology of TRPMs. (A) Phylogenetic tree of the human TRPM1–8 proteins. (B) TRPMs contain the following domains: MHR1–4, pre-S1, S1–S6, amphiphilic helices; PH, PL, pore-forming loop; TRP, highly conserved TRP helix; CC domain, NUDT9-H in TRPM2; *α*-Kinase in TRPM6 and TRPM7.

The founding member of the TRPM gene subfamily was identified as transcripts enriched in melanomas and, therefore, named melastatin (now *TRPM1*; Table 1).^5^^,^^7^ The human TRPM gene family consists of 8 members. Based on amino acid similarity, TRPM proteins form 2 phylogenetic groups, TRPM1/3/6/7 and TRPM2/8/4/5, which can be further subdivided into 4 pairs of homologous channels: TRPM1/3, TRPM2/8, TRPM4/5, and TRPM6/7 (Fig. 4A).^5^^,^^7^^,^^627^^,^^628^ The structural organization and key biophysical characteristics were found to be conserved within pairs; however, with some exceptions.

### B Domain topology, assembly, and functional characteristics of individual TRPMs

#### 1 Domain topology and channel assembly

The domain organization of TRPMs is illustrated in Fig. 4B. The large N-terminus of TRPMs, comprising ∼70% of the total protein sequence, is unique among ion channels and subdivided into 4 melastatin homology regions (MHR1–4). The membrane-spanning segment of TRPMs, similar to other TRP channels, contains a small amphiphilic domain (pre-S1 helix) and 6 TM helices (S1–S6). The region between S5 and S6 forms a short PH and a pore-forming loop. The S6 helix is linked to a highly conserved TRP helix and a CC domain. TRPM proteins function as tetrameric channel complexes, with 4 S5–S6 segments forming a common membrane-spanning channel pore (Fig. 4B).

TRPMs function as homotetramers—4 subunits assemble to form a channel pore. In addition, the closely related TRPM1 and TRPM3 proteins, as well as TRPM6 and TRPM7, can form TRPM1/3 and TRPM6/7 heterotetrameric channels.629., 630., 631., 632., 633. Cryo-EM was successfully used to address high-resolution structures of TRPM2,634., 635., 636., 637., 638., 639., 640. TRPM3,641., 642., 643. TRPM4,644., 645., 646., 647., 648. TRPM5,^649^^,^^650^ TRPM7,651., 652., 653. and TRPM8.^40^^,^^41^^,^654., 655., 656. These findings provided new mechanistic insights into structure-function relationships of TRPMs.^657^^,^^658^

Three TRPMs contain additional C-terminal segments. TRPM2 contains the nudix hydrolase 9 homology (NUDT9-H) domain (Fig. 4B).^659^^,^^660^ NUDT9 proteins cleave ADP-ribose (ADPR) into AMP and ribose-5-phosphate.^661^ NUDT9-H of human TRPM2 binds ADPR but does not exhibit enzymatic activity, while invertebrate TRPM2 proteins retain the capability to cleave ADPR.^640^^,^^662^

The C-terminal domains of TRPM6 and TRPM7 encode *α*-type kinase domains (Fig. 4B).663., 664., 665. *α*-Kinases are a group of atypical serine/threonine protein kinases with low primary sequence similarity to conventional protein kinases.^666^^,^^667^ C-terminal regions in other TRPM proteins do not contain enzymatic domains (Fig. 4B).

Alternative mRNA splicing creates additional diversity among TRPMs. *TRPM1* is expressed as a “long” active variant and as a “short” transcript that lacks the sequence encoding the TM channel segment.^668^ Alternative mRNA processing of *TRPM2* results in truncated channel versions with different functional characteristics.669., 670., 671. Alternative splicing of the pore-coding sequence in *TRPM3* produces channels with distinct cation selectivity.^672^ *TRPM4* is expressed as TRPM4a and TRPM4b variants with low and high channel activity, respectively.^673^^,^^674^ Alternative splicing of *TRPM6* and *TRPM7* creates isoforms encoding the N-terminal segments directly fused to the *α*-kinase domains.^629^^,^^675^

#### 2 Functional characteristics

TRPMs have been extensively investigated, and despite the overall structural similarity, they differ significantly in functional characteristics and cellular roles.^657^^,^^658^ The phylogenetic group of TRPM1, 3, 6, and 7 (Fig. 4A) represents channels that are highly permeable to divalent cations, including Zn^2+^, Mg^2+^, and Ca^2+^, and are regulated by PIP_2_ and intracellular Mg^2+^.

TRPM1, the founding member of the TRPM subfamily, forms a constitutively active channel highly expressed in melanocytes and the retina.^633^^,^^676^ The constitutive activity of TRPM1 can be further stimulated by the neurosteroid pregnenolone sulfate (PS) and inhibited by extracellular Zn^2+^ or intracellular Mg^2+^ ions.^633^ As mentioned above, *TRPM3* produces several alternatively spliced variants,^677^^,^^678^ including TRPM3*α*1 and TRPM3*α*2. TRPM3*α*1 contains a longer pore-forming segment between S5 and S6 and is highly permeable to Na^+^ ions.^672^ TRPM3*α*2 contains a shorter pore-forming sequence and is characterized by a high permeability to divalent cations.^672^^,^^679^ In addition, the S1–S4 regions of TRPM3*α*2 contains a noncanonical ion permeation mechanism called “omega” Na^+^ currents.^680^,^681^ TRPM3*α*2 is the most studied channel variant referred to herein as TRPM3. TRPM3 is negatively regulated by intracellular Mg^2+^ and PIP_2_ depletion and stimulated by PS and other steroids.^672^^,^682., 683.^–^^684^ TRPM3 is modulated by osmolality and D*-*erythro-sphingosine.^685^^,^^686^ Cryo-EM structures have demonstrated that PS activates TRPM3 through a site at the outer region of the channel pore formed by the PH and S1.^642^ The TRPM3 channel is activated by heat, underlying the temperature responses of the dorsal root and trigeminal ganglia neurons.^687^^,^^688^ Stimulation of receptors coupled to G_i_ and G_o_ causes inhibition of the channel through the direct assembly of the G_*βγ*_ subunits with TRPM3.^641^^,^^689^^,^^690^

TRPM6 and TRPM7 are homologous bifunctional proteins containing TM channel segments fused to cytosolic kinase domains and, therefore, are frequently named channel kinases.^5^^,^^7^^,^^627^ Thus, one TRPM6 or TRPM7 tetramer will form a typical TRP channel unit linked to 4 cytosolic kinase domains.^5^^,^^7^^,^^627^ Furthermore, recent proteomic studies demonstrated that ARL15, PPT4A1–3, and CNNM1–4 proteins coassemble to such channel-kinase complexes.^691^^,^^692^ Channel complexes formed by TRPM6 and TRPM7 are highly permeable to divalent cations and are negatively regulated by cytoplasmic Mg^2+^ and Mg·ATP as well as membrane levels of PIP_2_.^664^^,^^665^^,^^675^^,^693., 694., 695., 696., 697., 698., 699., 700., 701. The cryo-EM structures of the truncated TRPM7 have been resolved in the closed and open states.651., 652., 653. The solved structures are consistent with the idea that the lower channel gate contains the regulatory Mg^2+^ binding site.^702^

The *α*-kinase domains of TRPM6 and TRPM7 display low amino acid sequence homology to conventional serine/threonine kinases.^667^ However, the crystal structure of the TRPM7 kinase reveals considerable structural similarity to other protein kinases.^666^ The mass spectrometry approach identified multiple autophosphorylation sites mainly located in a serine/threonine-rich region situated upstream of the kinase domain of TRPM7.^691^^,^^703^^,^^704^ Similar to TRPM7 kinase, TRPM6 kinase can phosphorylate its own serine/threonine residues.^703^ In immune cells, the TRPM7 kinase domain can be cleaved from the channel domain by caspases upon Fas-receptor stimulation.^705^^,^^706^ Other studies have reported that cleaved TRPM6 and TRPM7 kinases are detected in the cell nucleus, where they can phosphorylate histones.^706^^,^^707^ Currently, the known phosphorylation substrates of TRPM6 and TRPM7 kinases comprise ∼20 proteins with varied subcellular locations and functional roles.708., 709., 710., 711., 712., 713., 714., 715., 716. Overall, such functional diversity makes it challenging to develop a unified model of the cellular roles of the TRPM6 and TRPM7 kinase domains.

The TRPM2, 4, 5, and 8 phylogenetic group (Fig. 4A) comprises channels with diverse functional characteristics. The TRPM2 and TRPM8 channels are permeable to divalent and monovalent cations and are often called nonselective cation channels or Ca^2+^-permeable cation channels. By contrast, TRPM4 and TRPM5 are impermeable to Ca^2+^ and are thus frequently called monovalent cation-selective channels.

TRPM2 was recognized as an unusual TRP channel due to the presence of the C-terminal NUDT9-H domain. TRPM2 is directly activated by intracellular ADPR.^659^^,^717., 718., 719., 720., 721., 722., 723. Recently, cryo-EM analysis of TRPM2 has identified 2 ADPR-binding pockets located in the MHR1/2 and NUDT9-H domains.634., 635., 636., 637., 638., 639. Both sites play a role in the opening of the TRPM2 channel. However, the interaction of ADPR with MHR1/2 underpins the prime regulatory mechanism in the human TRPM2 channel.634., 635., 636., 637., 638., 639. In addition, intracellular Ca^2+^ and membrane PIP_2_ were identified as crucial physiological ligands of TRPM2 required for channel opening by ADPR.^662^^,^724., 725., 726., 727., 728. The Ca^2+^ binding site is formed by acidic side chains of residues located in the S2 and S3 helices and the TRP domain of TRPM2.634., 635., 636., 637., 638., 639. PIP_2_ is found in a cavity often called a “vanilloid binding pocket.”^634^ TRPM2 is positively regulated by warm temperatures (>35 °C).729., 730., 731., 732. The structural basis for the temperature sensitivity of TRPM2 remains puzzling. The TRPM2 channel was also suggested as a cellular redox sensor because it is activated by peroxides, like H_2_O_2_, or other agents that produce reactive oxygen species (ROS).^733^ However, ROS act indirectly on TRPM2, likely due to the elevation of intracellular ADPR.^721^^,^^722^^,^^726^^,^^734^^,^^735^

TRPM8 shares significant structural homology with TRPM2. However, the C-terminal segment of TRPM8 lacks NUDT9-H. TRPM8 is activated by cold (<23–28 °C) and chemical agents evoking a sensation of coolness, including menthol and icilin.^14^^,^^405^^,^^736^^,^^737^ TRPM8 is a voltage-dependent channel.^406^^,^^738^ Analogously to TRPM2, the channel activity of TRPM8 is critically dependent^42^^,^^739^ on PIP_2_ and intracellular Ca^2+^. The depletion of PIP_2_ prevents channel opening by pharmacological compounds and cold,^42^^,^739., 740., 741. whereas Ca^2+^ is required for TRPM8 activation by icilin.^40^^,^^656^ Cryo-EM studies of TRPM8 have identified the binding sites of Ca^2+^, PIP_2_, and cooling agonists.^40^^,^^41^^,^654., 655., 656.^,^^742^ The location of Ca^2+^ and PIP_2_ binding sites in TRPM8 parallels the corresponding sites in TRPM2.^40^^,^^41^^,^654., 655., 656.^,^^742^ Cooling agents acoltremon (WS-12) and icilin interact with a binding pocket formed by the S1–S4 helices and the TRP domain of TRPM8, frequently called the VSLD.^40^^,^^41^^,^654., 655., 656.^,^^742^^,^^743^ However, the structural basis of the effects evoked by voltage and temperature on TRPM8 has not yet been established.^742^^,^^744^^,^^745^

TRPM4 and TRPM5 display functional characteristics that are unique among TRP channels—both channels are selective for monovalent cations and are activated upon increases in cytosolic Ca^2+^ levels.^673^^,^^674^^,^746., 747., 748. Two Ca^2+^-binding pockets are found in TRPM4 and TRPM5.644., 645., 646., 647., 648., 649., 650. One site is evolutionarily conserved, and, like in TRPM2 and TRPM8, it is formed by negatively charged residues in the TM domains of TRPM4 and TRPM5.648., 649., 650. This site is primarily responsible for the Ca^2+^-dependent opening of the TRPM4 and TRPM5 channels.648., 649., 650. Another Ca^2+^-binding site is unique to TRPM proteins and is located at the interface of the MHR1/2 and MHR3/4 cytosolic domains.648., 649., 650. In TRPM4, the interaction of Ca^2+^ with the cytosolic domain regulates a complex conformational temperature transition of the channel.^648^ In TRPM5, the binding of Ca^2+^ to this site affects the structural dynamics of the N-terminal domain, which subsequently regulates the voltage- and Ca^2+^-dependent opening of the channel.^649^^,^^650^ In addition, TRPM4 and TRPM5 are regulated by PIP_2_.^673^^,^^674^^,^746., 747., 748., 749. ATP is a negative modulator of TRPM4.^749^^,^^750^ ATP binds to TRPM4 through a site located at the interface of the MHR1/2 domains, and this interaction is temperature-dependent.^644^ Importantly, ATP does not act on TRPM5.^649^^,^^749^^,^^750^ TRPM5 was found to be a heat-sensitive channel that contributes to the temperature-dependent reception of chemical stimuli by taste receptor cells.^751^^,^^752^

### C Expression pattern and primary physiological roles

#### 1 TRPM1 and TRPM3

Initially, TRPM1 was identified as a transcript enriched in human melanomas and was suggested to be a potential tumor suppressor.^753^^,^^754^ However, the particular function of TRPM1 in melanomas and skin melanocytes remains unclear. The pathophysiological role of TRPM1 has been extensively investigated in the context of ON-bipolar neurons, which form synapses with rod, cone, and horizontal cells in the retina,^676^^,^755., 756., 757. because LOF mutations in the human *TRPM1* gene cause congenital stationary night blindness.757., 758., 759., 760., 761. In the dark, rod cells secrete glutamate, which activates metabotropic glutamate receptor 6 (mGluR6) in postsynaptic ON-bipolar neurons. mGluR6 is a G_o_ protein-coupled receptor, and its activation leads to the release of G_*α*(o)_ and G_*βγ*_. Both subunits directly interact and deactivate the TRPM1 channel.^676^^,^762., 763., 764. Exposure to light blocks the release of glutamate and inactivates mGluR6, leading to the opening of the TRPM1 channel and depolarization of ON-bipolar neurons.^676^^,^762., 763., 764. Importantly, stationary night blindness was developed by *Trpm1* KO mice, confirming the monogenic basis of the disease.^756^

TRPM3 is highly expressed in nociceptive neurons, pancreatic *β* cells, the brain, and several other tissues.^677^ In pancreatic islets, TRPM3 functions as an ionotropic steroid receptor responsible for PS-induced Ca^2+^ influx in *β* cells, leading to enhanced insulin secretion.^682^ In addition, TRPM3 can underlie PS-stimulated Zn^2+^ uptake in *β* cells, the crucial factor for insulin-containing dense core vesicles.^679^ PIP_2_ is directly associated with TRPM3.^641^^,^^683^^,^^765^ In insulinoma cells, stimulation of G_q_ protein-coupled receptors leads to the stimulation of PLC, depletion of its substrate PIP_2_, and inhibition of TRPM3.^683^ As mentioned above, activation of GPCRs leads to the inhibition of TRPM3 through the direct association of G_*βγ*_ with the channel.^641^^,^^689^^,^^690^ This regulatory mechanism contributes to the antinociceptive effects of *μ* opioid receptor agonists in DRG neurons.^689^ The TRPM3 channel is required for the temperature sensitivity of DRG and trigeminal ganglia neurons.^687^^,^^688^^,^766., 767., 768. *Trpm3* KO mice exhibited diminished sensitivity to noxious heat and reduced inflammatory heat hyperalgesia.^687^^,^^688^ Consequently, TRPM3 was suggested as a new analgesic drug target.^769^

#### 2 TRPM6 and TRPM7

TRPM7 is a ubiquitously expressed channel. Endogenous Mg^2+^-regulated TRPM7 currents have been found in virtually all primary isolated cells and stable cell lines examined, supporting the notion that TRPM7 is a versatile channel that plays a housekeeping cellular role.^675^^,^^694^^,^^702^^,^770., 771., 772., 773., 774., 775. To this end, independent evidence^693^^,^^694^^,^776., 777., 778. supports the concept that the TRPM7 channel represents the principal route for the cellular uptake of divalent cations, especially Mg^2+^. In line with this assumption, the genetic disruption or pharmacological inhibition of TRPM7 causes cell cycle arrest.^693^^,^^694^^,^^776^ Besides the homeostatic control of cellular Mg^2+^ and Zn^2+^ contents, TRPM7 is recognized as a Ca^2+^ channel shaping Ca^2+^-dependent cellular pathways779., 780., 781., 782. and a vesicular Zn^2+^ release channel.^695^ In this context, tissue-specific ablation of *Trpm7* in mice was used to elucidate the role of TRPM7 in the systemic balance of divalent cations. Unexpectedly, kidney-restricted deletion of *Trpm7* in mice did not cause apparent changes in the development, physical appearance, and biochemical characteristics of biological fluids.^693^ In another mouse strain, *Trpm7* was inactivated in enterocytes throughout the whole intestine, including the colon.^693^ Newborn mutants displayed growth failure and 100% mortality before weaning. Moreover, *Trpm7*-deficient pups displayed low Zn^2+^, Mg^2+^, and Ca^2+^ levels in serum, urine, and bones. Nutritional Zn^2+^ and Mg^2+^ supplementation of breastfeeding females extended the lifespan of mutant pups. These findings^693^ support the concept that intestinal TRPM7 operates as a master regulator of the body’s balance of Zn^2+^, Mg^2+^, and Ca^2+^.

Transgenic mouse models have been extensively used to investigate the role of TRPM7 in prenatal development (reviewed by Chubanov et al^665^^,^^783^). Among other exciting findings, it was demonstrated that TRPM7 is indispensable for early embryo development.^784^^,^^785^ Also, TRPM7 is abundantly expressed in gametes.784., 785., 786., 787. KO of *Trpm7* reduced Mg^2+^ and Zn^2+^ levels in oocytes and 4-cell embryos, leading to arrested embryonic development at the blastocyst stage.^786^ Interestingly, Mg^2+^ but not Zn^2+^ supplementation rescues the arrest of *Trpm7*-deficient zygotes.^786^ These findings correlate well with the in vitro examination of mouse embryonic stem cells and embryonic trophoblast stem cells, demonstrating that the loss of TRPM7 function leads to Mg^2+^-dependent proliferation arrest.^776^^,^^788^ In other studies, conditional mutagenesis of *Trpm7* at different embryonic stages has demonstrated that TRPM7 is indispensable for organogenesis of the kidney, heart, CNS, and immune organs.789., 790., 791.

Unlike the ubiquitously present TRPM7 channel, the expression of TRPM6 is limited to transporting epithelial cells of the placenta, kidneys, and intestine.^629^^,^^788^^,^^792^^,^^793^ The necessity for epithelial cells to express both TRPM6 and TRPM7 remains a topic of debate.^663^^,^^794^ Some studies suggest that the TRPM6 channel represents the close functional homolog of TRPM7 and that both proteins operate independently.^632^^,^^795^^,^^796^ An alternative view is that TRPM6 assembles with TRPM7 in heteromeric channels, which are less susceptible to metabolic negative control by cytosolic Mg·ATP.629., 630., 631.^,^^788^^,^^797^

TRPM6 was found to be highly expressed in the DCT segment of the kidney and in enterocytes of the gastrointestinal tract.^792^^,^^793^ Consequently, a comparative examination of mice with global versus kidney- or intestine-specific deletions of *Trpm6* was conducted.^788^ Mice lacking *Trpm6* in the whole body or specifically in the intestine exhibited severe hypomagnesemia and depletion of Mg^2+^ in bones due to impaired intestinal uptake of Mg^2+^ ions.^788^^,^^798^ Dietary Mg^2+^ supplementation fully normalized the biochemical and physiological characteristics of *Trpm6*-deficient mice.^788^ In contrast, 2 independent mouse strains with a kidney-specific KO of *Trpm6* displayed little or no impact on serum Mg^2+^ levels of mutant mice.^788^^,^^799^ These findings aligned with experiments^693^ involving kidney- versus intestine-specific deletions of *Trpm7*. Hence, the traditional kidney-centric view on the organismal balance of divalent cations needs some adjustment.

#### 3 TRPM2 and TRPM8

TRPM2 is a ubiquitously expressed channel implicated in many physiological processes, including insulin secretion by pancreatic *β* cells, Ca^2+^ signaling in immune cells, and body temperature sensation by somatosensory and hypothalamic neurons.^721^^,^^722^^,^729., 730., 731.^,^800., 801., 802., 803., 804., 805., 806., 807., 808., 809., 810., 811., 812., 813., 814., 815., 816., 817., 818., 819. TRPM2 is implicated in pathophysiological conditions linked to excessive ROS production, for instance, inflammation, neurodegenerative disorders, and IR injury.^808^^,^^809^^,^^818^^,^820., 821., 822., 823., 824., 825., 826., 827., 828., 829., 830., 831. IR injury is characterized by increased tissue levels of ROS leading to Ca^2+^ overload, cell death, and inflammatory processes.^829^ In this context, pharmacological inhibition of TRPM2 was suggested as a new strategy for treating IR injury.^828^

The physiological role of TRPM8 was investigated in genetic mouse models.^832^^,^^833^ *Trpm8* KO mice showed behavioral deficiency after exposure to cold temperatures.834., 835., 836., 837. TRPM8 is defined as the principal mediator of acute and inflammatory pain and irritation-induced reflexes.834., 835., 836., 837., 838., 839., 840., 841., 842., 843., 844., 845., 846. TRPM8 is abundantly expressed in the nerve endings of DRG neurons innervating the urinary bladder and contributes to symptoms of urinary urgency and other bladder reflexes.^847^^,^^848^ Consequently, TRPM8 has been proposed as a new target for the treatment of pain, cancer, and other disorders.

#### 4 TRPM4 and TRPM5

As TRPM4 and TRPM5 are impermeable to divalent cations, including Ca^2+^, their activation leads to Na^+^ influx and depolarization of the plasma membrane.^674^ In electrically nonexcitable cells, the opening of TRPM4 and TRPM5 reduces the driving force for Ca^2+^ entry through Ca^2+^-permeable channels.^752^^,^849., 850., 851., 852. In excitable cells, like cardiomyocytes and neurons, depolarization of the cell membrane opens voltage-activated Ca^2+^ channels.^752^^,^849., 850., 851., 852. TRPM4 is a ubiquitously expressed channel, and its role in shaping cellular responses to external stimuli is well documented in diverse immune and endocrine cells, cardiomyocytes, and neurons.^752^^,^849., 850., 851., 852.

TRPM5 is highly expressed in type II taste receptor cells, mediating responses to sweet, amino acids, and bitter compounds.^853^^,^^854^ In taste cells, the activation of GPCRs leads to PLC*β*2-evoked release of Ca^2+^ from intracellular stores and the opening of the TRPM5 channel.852., 853., 854., 855. The activation of TRPM5 causes membrane depolarization, the opening of voltage-gated Ca^2+^ channels, and consequently, the Ca^2+^-dependent release of the transmitter ATP.852., 853., 854., 855. Accordingly, deletion of *Trpm5* in mice impaired taste reception.^751^^,^^854^ TRPM5 was found to be a heat-sensitive channel, and this characteristic contributes to the temperature-dependent reception of chemical stimuli by taste receptor cells in the tongue.^751^^,^^752^

In addition, TRPM5 was identified as a prime transduction channel in chemosensory tuft cells, also known as brush cells.856., 857., 858., 859., 860., 861., 862., 863., 864., 865., 866., 867. Tuft cells are solitary epithelial cells containing apical “brush-like” microvilli that are present in many internal organs, including the respiratory system, thymus, gall bladder, urethra, and gastrointestinal tract.^868^^,^^869^ Tuft cells are crucial players in type 2 immune responses because they can detect pathogenic helminths, bacteria, and viruses.^858^^,^867., 868., 869., 870., 871., 872., 873., 874., 875., 876., 877. Upon activation, tuft cells release leukotrienes, acetylcholine, interleukin-25, and ATP, mobilizing tissue-resident immune cells and other protective responses.^858^^,^867., 868., 869., 870., 871., 872., 873., 874., 875., 876., 877., 878.

### D Human diseases associated with TRPMs

Gene association studies revealed the causal role of TRPMs in several human disorders. Thus, LOF mutations in the human *TRPM1* gene cause congenital stationary night blindness (type 1C), leading to impaired mGluR6/G_o_/TRPM1 signaling in ON-bipolar neurons in the retina.757., 758., 759., 760., 761.

De novo heterozygous point mutations in *TRPM3* have been identified in patients with developmental and epileptic encephalopathy (DEE).^879^^,^^880^ DEE is a group of chronic encephalopathies characterized by epilepsy and intellectual disability.^879^ Electrophysiological analysis of TRPM3 revealed that DEE-associated mutations represent GOF mutations.881., 882., 883. Pharmacological inhibition of TRPM3 by primidone has been demonstrated as a potential treatment for DEE patients.880., 881., 882., 883., 884.

LOF mutations in the human *TRPM6* gene give rise to a disorder known as primary hypomagnesemia type 1, intestinal (HOMG1).^648^^,^885., 886., 887. HOMG1 patients are typically infants presenting with generalized convulsions, muscle spasms, and very low blood levels of Mg^2+^ and Ca^2+^. Supplementation with high doses of Mg^2+^ in patients relieves hypomagnesemia and all other symptoms, including hypocalcemia.^792^^,^^793^ Therefore, this disorder is frequently called primary hypomagnesemia with secondary hypocalcemia.^792^^,^^793^^,^^888^ Clinical assessment of the first HOMG1 patients revealed that hypomagnesemia developed due to defective intestinal Mg^2+^ uptake.885., 886., 887. In follow-up studies, renal leak of Mg^2+^ was also detected in Mg^2+^-supplemented HOMG1 individuals.^792^^,^^793^^,^^888^

Missense mutations in *TRPM7* have been linked to stillbirth.^889^ Stillbirth is defined as the loss of a fetus after 22 weeks of gestation during pregnancy.^890^ Worldwide, the stillbirth rate is ∼14 cases per 1000 births, and the etiology of this disease remains poorly understood.^890^ Recently,^889^ sequencing of tissue samples from affected fetuses revealed heterozygous nonsynonymous variants in *TRPM7*. Upon heterologous expression, introducing 2 mutations in *TRPM7* caused a reduction in channel activity, whereas 2 other substitutions led to proteasomal degradation of TRPM7.^890^ However, the exact physiological process impaired by these mutations in *TRPM7* has not been established yet.

A new form of macrothrombocytopenia has been linked^891^ to missense substitutions in *TRPM7*. Macrothrombocytopenia is a group of disorders characterized by abnormally large platelets due to their impaired formation in megakaryocytes.^891^ The affected patients were heterozygous for LOF point mutations in *TRPM7* and displayed reduced Mg^2+^ levels in platelets.^891^ Notably, a mouse strain with conditional megakaryocyte-restricted *Trpm7* KO also developed macrothrombocytopenia.^891^

Trigeminal neuralgia is a human disease defined by severe facial pain.^892^ Whole-exome sequencing identified 1 patient heterozygous for the A931T mutation affecting the S3 helix of TRPM7.^892^ Electrophysiological analysis of the A931T TRPM7 channel variant revealed atypical “omega” Na^+^ currents.^892^ Hence, it was proposed that these “omega” currents depolarize trigeminal ganglion neurons, causing pain in trigeminal neuralgia patients.^892^

Recently, mutations in *TRPM7* have been linked to an autosomal dominant variant of hypomagnesemia (low serum concentrations of Mg^2+^).893., 894., 895. The affected patients were heterozygous for LOF point mutations in *TRPM7*. Apart from hypomagnesemia, the patients displayed other less prominent symptoms, including episodes of hypocalcemia (low serum concentrations of Ca^2+^), seizures, and muscle cramps. In addition, some individuals suffered from migraine, autism, and developmental delays, mainly affecting speech and motor skills. Notably, supplementation with high doses of Mg^2+^ in patients could only partially normalize serum concentrations of Mg^2+^ and incompletely ameliorate other symptoms.^893^^,^^894^

GOF and LOF point mutations in the human *TRPM4* gene have been linked to different forms of cardiac conduction defects, including progressive familial heart block type I,^896^^,^^897^ Brugada syndrome,898., 899., 900., 901. right-bundle branch block, atrioventricular block, and complete heart block.902., 903., 904. However, it remains puzzling why either reduced or increased TRPM4 activity leads to different forms of cardiac conduction defects.

### E Pharmacological modulators of TRPMs

As outlined above, TRPMs critically contribute to diverse physiological processes and are considered prospective drug targets for the treatment of human diseases.^657^^,^^658^ Consequently, numerous studies have been conducted to identify small organic compounds suitable for the pharmacological regulation of TRPMs in cultured cells and animal disease models.^657^^,^^658^ Herein, we summarize the key developments in these research areas and discuss the identified pharmacological modulators of TRPMs. However, the present chapter will not cover the effects of nonspecific channel inhibitors (eg, ruthenium red and 2-aminoethoxydiphenyl borate) or compounds incompletely characterized in terms of their potency and efficacy.

#### 1 TRPM1 and TRPM3

The pharmacological toolkit for TRPM1 has not yet been developed. TRPV1 agonists, capsaicin and anandamide, were used to activate endogenous TRPM1 currents in ON-bipolar cells.^757^ The response of ON-bipolar cells to capsaicin was blocked by the TRPV1 inhibitor capsazepine.^757^ Similarly, an antibiotic agent, voriconazole, was suggested to inhibit capsaicin-evoked TRPM1 currents in ON-bipolar cells.^905^ However, evidence of the direct action of these compounds on the TRPM1 channel and the pharmacological characteristics of such interactions (eg, IC_50_) remains to be seen.

Several synthetic compounds positively regulate TRPM3 channel activity, including CIM0216, clotrimazole, and nifedipine (Table 4).^681^^,^682., 906., 907., 908., 909. CIM0216 was determined to be the most potent activator of the TRPM3 channel.^906^ The antifungal agent clotrimazole causes potentiation of the TRPM3 channel, as this compound does not affect basal or heat-activated TRPM3 currents but robustly stimulates TRPM3 upon coapplication with PS.^681^ In addition, several potent inhibitors of TRPM3 have been identified. Thus, the FDA-approved drugs diclofenac, maprotiline, and primidone were found to be potent inhibitors of PS-induced TRPM3 activity (Table 4).^907^ Notably, primidone could attenuate thermal nociception in animals.^907^ Recently,^642^ cryo-EM structures of TRPM3 were addressed in complex with PS, primidone, and CIM0216. While PS interacts with TRPM3 through a site at the outer region of the channel pore, primidone, nifedipine, and CIM0216 bind to TRPM3 within the cavity between the S1–S4 segments and the TRP domain.^642^^,^^643^ Another study demonstrated that 2 fruit flavanones, naringenin and hesperetin, and the spiny restharrow derivative, ononetin, are potent inhibitors of TRPM3 (Table 4).^908^ Follow-up hit optimization experiments uncovered fruit flavanones isosakuranetin and liquiritigenin, displaying improved potency in the block of TRPM3 currents (Table 4).^909^ Moreover, isosakuranetin and hesperetin were capable of reducing the sensitivity of mice to noxious heat and PS-induced pain.^909^

**Table 4:** Pharmacological modulators of TRPM3

Name (PubChem CID^a^)	Effect	References
CIM0216 (42887770)	Activation, EC_50_ = 0.77 *μ*M	^906^
Clotrimazole (2812)	Potentiation, EC_50_ = 20 nM	^681^
Nifedipine (4485)	Activation, EC_50_ = 30–32 *μ*M	^682^
Diclofenac (3033)	Inhibition, IC_50_ = 6.2 *μ*M	^907^
Maprotiline (4011)	Inhibition, IC_50_ = 1.3 *μ*M	^907^
Primidone (4909)	Inhibition, IC_50_ = 0.6 *μ*M	^907^
Naringenin (439246)	Inhibition, IC_50_ = 0.5 *μ*M	^908^
Hesperetin (72281)	Inhibition, IC_50_ = 2.0 *μ*M	^908^
Ononetin (259632)	Inhibition, IC_50_ = 0.3 *μ*M	^908^
Isosakuranetin (160481)	Inhibition, IC_50_ = 50 nM	^909^
Liquiritigenin (114829)	Inhibition, IC_50_ = 0.5 *μ*M	^909^

a PubChem Compound Identification number.

#### 2 TRPM6 and TRPM7

Several small molecules have been defined as negative regulators of the TRPM7 channel.^664^^,^910., 911., 912. A significant fraction of these agents represent polyspecific channel blockers, incompletely characterized compounds, or low-potency antagonists of TRPM7.^664^^,^^910^^,^^911^ However, NS8593, waixenicin A, FTY720, VER155008, CCT128930, cannabigerolic acid, and cannabidivarin were found to be potent inhibitors of TRPM7 currents with IC_50_ values in the low micromolar to nanomolar range (Table 5).913., 914., 915., 916., 917., 918., 919., 920., 921., 922. Noteworthy, waixenicin A, VER155008, CCT128930, and cannabigerolic acid selectively suppressed the TRPM7 channel and displayed no effects on the homologous TRPM6 channel.^652^^,^^653^^,^^914^^,^^915^^,^^919^ In contrast, NS8593 and FTY720 inhibited both channels, TRPM6 and TRPM7.^652^^,^^653^^,^^915^^,^^917^ NS8593, waixenicin A, and FTY720 were the most extensively used to map the cellular roles of the TRPM7 channel in different physiological and pathophysiological settings, including animal models of human diseases, such as tissue fibrosis, metabolic, cardiovascular, and immune disorders, and treatment of tumors, inflammation, and aortic aneurysm.^715^^,^^782^^,^923., 924., 925., 926., 927., 928., 929., 930., 931., 932., 933., 934., 935.

**Table 5:** Pharmacological modulators of TRPM6 and TRPM7

Name (PubChem CID^a^)	Effect	References
TRPM7		

NS8593 (71311765)	Channel inhibition, IC_50_ = 1.6 *μ*M^b^ (3.9 *μ*M^c^)	^913^
Waixenicin A (73755210)	Channel inhibition, IC_50_ = 7.0 *μ*M^b^ (16 nM^c^)	^914^
FTY720 (107969)	Channel inhibition, IC_50_ = 0.72 *μ*M	^917^
VER155008 (25195348)	Channel inhibition, IC_50_ = 0.11 *μ*M	^915^
CCT128930 (17751819)	Channel inhibition, IC_50_ = 0.86 *μ*M^b^ (0.63 *μ*M^c^)	^916^
Cannabigerolic acid (CBDA) (6449999)	Channel inhibition, IC_50_ = 1.8 *μ*M	^919^
Cannabidivarin (CBDV) (11601669)	Channel inhibition, IC_50_ = 3.4 *μ*M	^919^
Naltriben (5486827)	Channel activation, EC_50_ = 21 *μ*M	^920^
Mibefradil (60663)	Channel activation, EC_50_ = 53 *μ*M	^921^
TG100-115 (10427712)	Kinase inhibition, IC_50_ = 1.07 *μ*M	^922^

TRPM6		

Iloperidone (71360)	Channel inhibition, IC_50_ = 0.73 *μ*M	^915^
Ifenprodil (3689)	Channel inhibition, IC_50_ = 3.33 *μ*M	^915^

a PubChem Compound Identification number.

b IC_50_ was determined in Mg^2+^-free intracellular saline.

c IC_50_ was determined in the presence of physiological Mg^2+^ concentration.

Recently,^652^^,^^653^ cryo-EM structures of TRPM7 were solved in complex with NS8593, VER155008, and CCT128930. All 3 inhibitors bind to the same site in TRPM7, located on the cytoplasmic side of the membrane at the interface of the S3, S4, and S5 helices and the TRP domain. This ligand-binding pocket in TRPM7 is called a vanilloid-like site because the homologous cavity in the TRPV1 channel has been previously defined as a vanilloid regulatory site.^652^^,^^653^ However, whether waixenicin A and FTY720 bind to the vanilloid-like site of TRPM7 or act through an alternative mechanism remains to be examined.

A set of small molecules serving as TRPM7 channel agonists has been identified.^920^^,^^921^ Among them, naltriben and mibefradil have been characterized in detail (Table 5). Both agents can potently activate TRPM7 currents without depletion of intracellular Mg^2+^, indicating that both compounds act as true agonists of the TRPM7 channel.^920^^,^^921^ Consequently, many studies employed naltriben and mibefradil, frequently in combination with TRPM7 inhibitors, to examine the role of this channel in different cellular processes.^664^^,^910., 911., 912. More recently, the cryo-EM structure of TRPM7 was solved in the open state in complex with naltriben.^653^ A comparison of the closed and open naltriben-bound structures of TRPM7 uncovered particular conformational rearrangements associated with agonist-induced activation of the TRPM7 channel. Naltriben-binding pockets (4 sites per tetramer) were found at the intersubunit interface, formed by the MHR4/pre-S1 helix of one subunit and the MHR4 domain of the neighboring subunit. Intriguingly, this ligand-binding site has not been identified in TRPMs before.^653^

The selective pharmacological modulators of TRPM7 kinase remain to be identified. Currently, only 1 compound, TG100-115 (Table 5), is known as an inhibitor of TRPM7 kinase activity, but this molecule also inactivates TRPM6 kinase.^630^^,^^691^^,^^922^

In contrast to TRPM7, the pharmacological profile of TRPM6 is less established. Recently, 2 structurally unrelated compounds, iloperidone and ifenprodil, were defined as potent inhibitors of the TRPM6 channel (Table 5).^915^ Notably, both reagents showed no impact on the TRPM7 channel.^915^ As mentioned above, NS8593 and FTY720 can suppress TRPM6 currents.^652^^,^^653^^,^^915^^,^^917^ Hence, the available pharmacological toolkit enables selective or combined targeting of TRPM6 and TRPM7 in physiological conditions or preclinical experimental models, for instance, in patient-derived primary cells.

#### 3 TRPM2 and TRPM8

H_2_O_2_- and ADPR-evoked TRPM2 currents can be blocked by several synthetic and natural compounds, including *N*-(*p*-amylcinnamoyl)anthranilic acid, tyrphostin AG 490 (AG490), clotrimazole, JNJ-28583113, scalaradial, and 2,3-dihydroquinazolin-4(1*H*)-one derivative D9 (Table 6).936., 937., 938., 939., 940., 941., 942., 943., 944., 945., 946., 947., 948., 949., 950., 951., 952., 953., 954., 955., 956. The generation of synthetic analogs of ADPR represents another strategy to target TRPM2. Thus, 8-phenyl-2′-*deoxy*-ADPR was found to be a potent inhibitor of TRPM2 currents.^942^ Two other synthesized ADPR analogs with substitutions in the pyrophosphate segment of the nucleotide (compounds 7i and 8a) displayed considerable potency and selectivity in the suppression of TRPM2 (Table 6).^943^ TatM2NX is a cell-permeable peptide designed to interact with ADRP binding in TRPM2.^944^ In electrophysiological experiments, TatM2NX was found to be a potent inhibitor of the TRPM2 channel (Table 6).^944^ Despite outstanding progress in structural assessment of TRPM2 channels from different species,634., 635., 636., 637., 638., 639. the molecular basis underpinning the inhibitory effect of the ligands mentioned above remains unknown. Also, it is worth noting that pharmacological compounds acting as agonists of the TRPM2 channel have not yet been identified.

**Table 6:** Selected examples of pharmacological modulators of TRPM2 and TRPM8.

Name (PubChem CID^a^)	Effect	References
TRPM2		

*N*-(p-Amylcinnamoyl)anthranilic acid (ACA) (5353376)	Inhibition, IC_50_ = 1.7 *μ*M	^936^
AG490 (5328779)	Inhibition, IC_50_ = 0.4 *μ*M	^937^
Clotrimazole (2812)	Inhibition, IC_50_ = ∼1 *μ*M	^938^
JNJ-28583113 (164628567)	Inhibition, IC_50_ = 0.13 *μ*M	^939^
Scalaradial (21637538)	Inhibition, IC_50_ = 0.21 *μ*M	^940^
2,3-dihydroquinazolin-4(1*H*)-one derivative D9^c^ (N/A)	Inhibition, IC_50_ = 3.7 *μ*M	^941^
8-phenyl-2′-*deoxy*-ADPR (compound 86^c^) (N/A)	Inhibition, IC_50_ = 3 *μ*M	^942^
ADPR analogues 7i^c^ and 8a^c^ (N/A)	Inhibition, IC_50_ = ∼5 *μ*M	^943^
TatM2NX (154699439)	Inhibition, IC_50_ = 0.40 *μ*M	^944^

TRPM8		

(–)-Menthol (16666)	Activation, EC_50_ = 48 *μ*M	^945^
Icilin (161930)	Activation, EC_50_ = 0.36 *μ*M	^946^
Acoltremon (WS-12) (11266244)	Activation, EC_50_ = 0.19 *μ*M	^947^
Azo-menthol (N/A)	Activation, EC_50_ = 4.4 *μ*M	^948^
(+)-Sesamin (72307)	Inhibition, IC_50_ = 9.8 *μ*M	^949^
Hispidulin (5281628)	Inhibition, IC_50_ = 1.7 *μ*M	^950^
Oroxylin A (5320315)	Inhibition, IC_50_ = 9.7 *μ*M	^950^
AMTB (16095383)	Inhibition, IC_50_ = ∼1 *μ*M	^951^
M8-B (69316632)	Inhibition, IC_50_ = 7.8 nM	^952^
TC-I 2000^b^ (compound 87^c^) (57326210)	Inhibition, IC_50_ = 36 nM	^953^
AMG 333 (71144018)	Inhibition, IC_50_ = 13 nM	^954^
RQ-00203078 (49783953)	Inhibition, IC_50_ = 8.3 nM	^955^
TC-I 2014^b^ (compound 5^c^) (135883253)	Inhibition, IC_50_ = 3 nM	^956^

a PubChem Compound Identification number. N/A – not available.

b Commercially available product.

c Referred as in reference.

TRPM8 has been proposed as a new target for the treatment of pain, and consequently, a very comprehensive collection of TRPM8 modulators has been developed. TRPM8 agonists like menthol, icilin, and WS-12 are broadly used to explore the pharmacological potential of this channel (Table 6).945., 946., 947. In addition to menthol, other natural products with menthol-like cooling effects are defined as activators of TRPM8, including camphor, rotundifolone, eucalyptol, and borneol.^957^ However, these compounds affect TRPM8 at a high micromolar range of concentrations and elicit multiple effects on other proteins.^957^^,^^958^ The structures of such cooling agents serve as blueprints for designing dozens of synthetic agents with EC_50_ values in the nanomolar range.^957^^,^^958^ These synthetic substances have been predominantly documented in patents from pharmaceutical companies and await further validation.^957^^,^^958^ Recently, the first photoswitchable TRPM8 activator, azo-menthol, has been developed, which enables optical regulation of TRPM8 currents with UV and blue light (Table 6).^948^

Screening natural products led to the discovery of TRPM8 inhibitors, such as sesamin, hispidulin, and oroxylin A (Table 6).^949^^,^^950^ In addition, a series of synthetic TRPM8 antagonists have been identified, for instance, AMTB, M8-B, TC-I 2000, AMG 333, RQ-00203078, and TC-I 2014 (Table 6).951., 952., 953., 954., 955., 956.^,^^959^ AMTB and TC-I 2014 were used in the cryo-EM analysis of TRPM8, and the resolved structures revealed that, analogously to agonists WS-12 and icilin, both inhibitors interact with a ligand-binding site formed by residues of the S1–S4 segments of TRPM8.^40^^,^^654^ Finally, it is worth noting that pharmaceutical companies have synthesized several potent inhibitors of TRPM8, but similar to the situation with TRPM8 activators, the functional impacts of these entities on TRPM8 are only briefly reported in patents.^957^^,^^958^

#### 4 TRPM4 and TRPM5

In initial studies, several polyspecific channel blockers were used to inhibit TRPM4 currents, for instance, 9-phenanthrol and MPB-104 (Table 7).^649^^,^960., 961., 962., 963., 964., 965., 966., 967., 968., 969., 970. However, these agents displayed a low potency toward TRPM4. Subsequently, more potent TRPM4 inhibitors were identified, such as 4-chloro-2-{[2-(2-chlorophenoxy)acetyl]amino} benzoic acid, 4-chloro-2-[2-(naphthalen-1-yloxy)acetamido] benzoic acid, and meclofenamate (Table 7).^962^^,^^963^^,^^971^ In mice, meclofenamate inhibited the Ca^2+^ overload-induced background current in ventricular cardiomyocytes and suppressed catecholaminergic polymorphic ventricular tachycardia-associated arrhythmias in a TRPM4-dependent manner.^963^ U73122 was found to be a potent activator of TRPM4, which can stimulate TRPM4 currents in the absence of intracellular Ca^2+^ (Table 7).^964^ Another compound, a 3,5-bis(trifluoromethyl)pyrazole derivative (YM-58483), is defined as a potentiator (or enhancer) of the TRPM4 channel because the degree of TRPM4 activation is dependent on the presence of intracellular Ca^2+^ (Table 7).^965^

**Table 7:** Pharmacological modulators of TRPM4 and TRPM5

Name (PubChem CID^a^)	Effect	References
TRPM4		

9-Phenanthrol (10229)	Inhibition, IC_50_ = 17–23 *μ*M	^960^
MPB-104 (11738767)	Inhibition, IC_50_ = 11–24 *μ*M	^960^
4-Chloro-2-{[2-(2-chlorophenoxy)acetyl]amino} benzoic acid (CBA^b^, compound 5^c^) (2264067)	Inhibition, IC_50_ = 1.8 *μ*M	^962^
4-Chloro-2-[2-(naphthalen-1-yloxy)acetamido] benzoic acid (NBA^b^, compound 6^c^) (1295523)	Inhibition, IC_50_ = 0.2 *μ*M	^962^
Meclofenamate (4038)	Inhibition, IC_50_ = 3.4 *μ*M	^963^
U73122 (104794)	Activation, EC_50_ = 0.44 *μ*M	^964^
3,5-Bis(trifluoromethyl)pyrazole derivative BTP2 (YM-58483) (2455)	Potentiation, EC_50_ = 8–500 nM	^965^
Necrocide 1 (NC1) (49783440)	Activation, EC_50_ = 0.31 *μ*M	^966^

TRPM5		

Triphenylphosphine oxide (TPPO^b^) (13097)	Inhibition, IC_50_ = 12 *μ*M	^967^
NDNA^b^ (674882)	Inhibition, IC_50_ = 2.4 nM	^649^
Stevioside (442089)	Potentiation, EC_50_ = 690 nM	^968^
Benzo[*d*]isothiazole derivatives 61^c^, 64^c^ (164611814, 164619590)	Activation, EC_50_ = 8–44 nM	^969^
Tetrahydroisoquinoline derivative 39^c^ (167993652)	Activation, EC_50_ = 80 nM	^970^

a PubChem Compound Identification number.

b Abbreviation of chemical name.

c Referred to as in the reference.

Recently, a small molecule, necrocide 1 (NC1), was identified as a potent activator of human TRPM4 but not mouse TRPM4 (Table 7).^966^ Interestingly, upon activation of TRPM4, NC1 induces necrotic cell death because of Na^+^ overload.^966^ Despite significant progress in cryo-EM analysis of TRPM4,644., 645., 646., 647., 648. the structural basis for the inhibitory and stimulatory effects of the ligands mentioned above remains unknown.

Several pharmacological agents, such as flufenamic acid, clotrimazole, and quinine, were found suitable for inhibiting the TRPM5 channel.^750^^,^^972^ However, these compounds were active in the high micromolar range and capable of suppressing TRPM4.^750^^,^^972^ Subsequently, triphenylphosphine oxide demonstrated improved selectivity and potency toward TRPM5, whereas *N*′-(3,4-dimethoxybenzylidene)-2-(naphthalen-1-yl)acetohydrazide (NDNA) represents the most potent inhibitor of TRPM5 currents (Table 7).^649^^,^^967^ Cryo-EM analysis demonstrated that NDNA binds to a cleft between the S1–S4 segment and the S5–S6 helices, known as the vanilloid binding site in TRPVs, stabilizing the channel in a closed conformation.^649^ Recently, NC1 was identified as a compound that induces necrotic cell death through direct activation of the TRPM4 channel through the NDNA-binding site (Table 7).^966^

Several natural compounds are applicable for the positive regulation of TRPM5 (Table 7). Steviol glycosides, such as stevioside, potentiate the Ca^2+^-dependent activity of the TRPM5 channel and are thus defined as potentiators of TRPM5.^968^ In other studies, high-throughput screening and lead optimization strategies suggested several synthetic compounds to act as potent TRPM5 agonists.^969^^,^^970^ Among several benzo[*d*]isothiazole derivatives, 2 molecules (referred to as compounds 61 and 64) activated the TRPM5 channel with EC_50_ values in the nanomolar range.^969^ A series of tetrahydroisoquinoline-based molecules (ie, compound 39) stimulated TRPM5 with an EC_50_ of 0.1–10 *μ*M (Table 7).^970^ However, a more comprehensive biophysical assessment is needed to conclude that these ligands open the TRPM5 channel in a Ca^2+^- and voltage-independent fashion.

### F Ongoing or completed clinical trials with TRPMs

According to the ClinicalTrials.gov database,^973^ TRPM3 is the subject of a clinical trial (NCT05275751) aimed at examining whether the redundant functions of TRPV1, TRPA1, and TRPM3 observed in mice regarding heat perception are also applicable to humans. Another trial (NCT03252834) is examining whether the genetic variants of *TRPM2* represent biomarkers for chemotherapy-induced abnormal thermal sensation in cancer patients. Early treatment of cerebral edema and intracranial pressure is crucial for improving outcomes. The study NCT06017635 investigates whether *TRPM4* expression levels can serve as a diagnostic marker for cerebral edema in children. It is well documented that early mobilization of patients in the surgical intensive care unit improves outcomes. The trial NCT01363102 examines whether genetic polymorphisms in *TRPM6* and other genes are linked to sleep quality and muscle strength, and whether these associations relate to early mobilization in surgical patients. The project NCT04229992 examines the association between SNPs in *TRPM7*, dietary intake of calcium and magnesium, and the risk of developing colorectal cancer.

Several ongoing clinical trials assess TRPM8 as a therapeutic target for various pathophysiological conditions. The trial NCT01408446 investigates the impact of the TRPM8 agonist menthol (Table 6) on the prevention of prehypertension and mild hypertension. This trial aims to assess the effects of dietary menthol on blood pressure and metabolic parameters. The study NCT05935280 aims to determine whether TRPM8 contributes to cold pain perception in humans. Cold pain will be experimentally induced by injecting a cooling solution (3 °C) into the skin, along with TRPM8 inhibitors to assess their effects. Experiments with animals indicated that activating TRPM8, which is expressed in the dermal tissue of the limbs, using menthol is beneficial for stroke recovery. The investigation NCT05877079 aims to examine the impact of such treatment on patients with acute ischemic stroke. The trials NCT04711044, NCT04554888, NCT04515056, and NCT03943407 evaluate the effects of menthol on itch induced by histamine, cowhage, and papain. The project NCT03610386 examines the effect of menthoxypropanediol, a derivative of menthol, on pruritus in atopic dermatitis (eczema) using biopsies from patients with atopic dermatitis. Applying menthol topically increases resting energy expenditure, likely by activating brown adipose tissue. The aim of the study NCT07030725 is to determine whether applying menthol to the front of the thorax will boost thermogenesis through brown adipose tissue activation and enhanced blood flow in skeletal muscles. The project NCT01565070 examines whether menthol can alleviate symptoms associated with knee osteoarthritis, thereby reducing immobility and isolation in older adults.

Recently, the FDA approved WS-12, a TRPM8 agonist (Table 6), for the treatment of symptoms associated with dry eye disease.^974^ WS-12 stimulates corneal nerves to promote natural tear production and has been found beneficial in 40% of patients over 90 days of treatment.^974^

On this background, we anticipate that the recently developed modulators of TRPMs will enable the design of new clinical trials in the near future.

## V TRPAs

### A TRPA gene family

In humans, TRPA1 is the only member of the ankyrin-repeat TRP channel subfamily (Table 1). It is a polymodal irritant sensor that is expressed in nociceptive neurons and some nonneuronal cell types. Its marked promiscuity to be activated by a plethora of natural products, drugs, and drug-like compounds sets it apart from most other TRP channels and often results in covalent modification or indirect mechanisms, which include the formation of ROS or oxidized membrane lipids.

### B Domain topology, assembly, and functional characteristics of TRPA1

#### 1 Domain topology and channel assembly

A prominent and name-giving property of TRPA1 is its extended N-terminal ARD, featuring approximately 16 (14–18, depending on the species) consecutive ankyrin-like folds that consist of about 33 amino acids each. This N-terminal ARD makes up most of the intracellular volume of the channel protein. The 3D structure of mammalian TRPA1 has been elucidated by cryo-EM, first at a rather low resolution of about 16 Å,^975^ and more recently, at a resolution of 4 Å^976^ or ∼3 Å,^977^ which allowed for a more reliable reconstruction. While the first 11 ankyrin repeats appear as a concave crescent-like density that extends away from the central symmetry axis, the ankyrin repeats 12–16 closely surround a central bundle of the 4 C-terminal located *α*-helical structures, which engage in CC helices with their respective neighbors. A prominent regulatory site is located in the linker region between the ARD and the first TM segment S1 region. This linker, also referred to as the coupling domain, contains cysteine (C621 and C641) and lysine (K710) residues that surround a binding pocket and can be covalently modified by electrophilic TRPA1-activating drugs.^977^

Like other TRP channels, the TMD can be subdivided into a VSLD comprising the first 4 TM-spanning segments S1–S4, a helical S4–S5 linker, a pore-forming fold that consists of TM helix S5, the re-entrant pore loop, and a tilted S6 helix, which strongly constricts the pore in its closed conformation. The pore loop features 2 short helical segments that position a string of 3 glutamate residues (E920, E924, and E930) to generate a negatively charged surface in and around the extracellular pore mouth. The most centrally positioned E920, together with D915, is a key feature of the selectivity filter that constricts the pore and divides the permeation pathway into an outer vestibule and an inner cavity.^978^ At present, structural data on the open channel state of TRPA1 are still lacking. Hypothetical models postulate a rotation of S6 that repositions the strongly constricting hydrophobic amino acids I957 and V961 away from the central axis, which then may be flanked by E966 and open to form a hydrophilic cation-conducting pore.^979^ The TMD is followed by a TRP-like domain and the aforementioned C-terminal CC-forming domain.

**Fig. 5:**
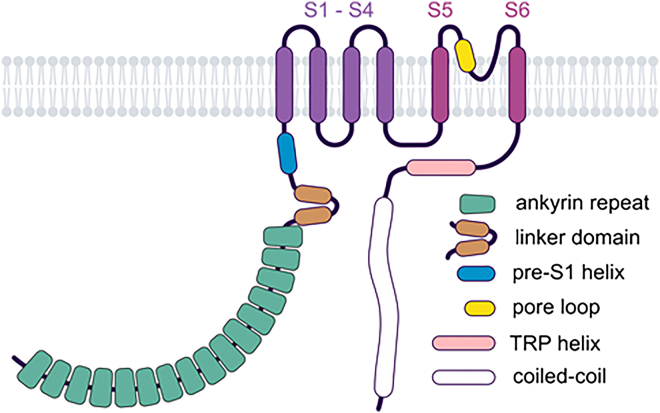
The domain topology of TRPA1. The TRPA1 channel contains the following domains: an ARD consisting of 16 consecutive ankyrin repeats, a helix-loop-helix-shaped linker domain that connects to the bundle of the first 4 TM-spanning helices via an *α*-helical pre-S1 segment. The selectivity filter and gate of the permeation pathway are formed by a recurrent pore loop and TM segments 5 and 6. The cytosolic C terminus features the highly conserved TRP helix and a CC domain, which engages in contacts with neighboring subunits.

Finally, the TMD features grooves and clefts that allow the noncovalent binding of allosteric TRPA1-modulating drugs. An intersubunit cleft between S4 and the S4–S5 linker of one subunit, and S5 and S6 of the neighboring subunit, has been demonstrated to adopt GNE551, a noncovalent TRPA1 activator.^980^ The TRPA1 antagonist A-967079 most likely binds to a pocket formed in the upper part of the TMD flanked by S5, S6, and the first PH.^976^ Figure 5 provides a graphical illustration of the domain topology of TRPA1.

All structural biology data on recombinantly expressed TRPA1 channel complexes confirm the expected homotetrameric conformation with rotational symmetry (see PDB entries provided in Table 1). Evidence of heteromeric channel assemblies composed of TRPA1 and TRPV1 subunits was obtained through coimmunoprecipitation analyses, Förster resonance energy transfer, single-channel current properties, and atomic force microscopy of heterologously expressed concatemers.^383^^,^^981^ The extent and physiological, pathophysiological, and pharmacological relevance of a possible heteromeric assembly have not yet been clearly defined. Moreover, the complexes may either result from a heterotetrameric assembly, as suggested by Fischer et al,^383^ or from functional interaction of side-by-side assemblies of homotetrameric complexes that may interact directly via a bridging protein,^982^ such as TMEM100, or by an A kinase-anchoring protein (AKAP79/150) as a scaffold.^983^

#### 2 Functional characteristics of TRPA1 channel complexes

Due to its responsiveness to numerous compounds and conditions and its strong expression in nociceptive or chemosensory afferent neurons, there is a broad consensus that TRPA1 acts as a polymodal irritant sensor. A plethora of input queues converge toward TRPA1 activation, including the covalent binding of cysteine-reactive or other electrophilic activators,^984^^,^^985^ the indirect effects of compounds that induce lipid peroxidation, such as the formation of 4-hydroxynonenal,^986^ ultraviolet light, or phototoxic substances that can generate ROS upon illumination,^987^^,^^988^ ROS themselves,^989^ as well as nonreactive TRPA1 activators, some of which are summarized below. In addition, TRPA1 activity can be further potentiated by intra- and extracellular Ca^2+^ ions,990., 991., 992. or via GPCRs that sense inflammatory mediators.^993^^,^^994^

Initial findings suggesting that TRPA1 may sense noxious cold temperatures^995^ were soon challenged,^996^ and the results strongly relied on the investigated species.^997^^,^^998^ Genetic as well as pharmacological evidence has even attributed TRPA1 to contribute to a heat-sensing pathway.^766^ Human TRPA1 seems to be both cold- and heat-sensitive, and structural determinants for thermal activation have been identified.^999^

With respect to its biophysical properties, TRPA1 mediates poorly selective cation currents that show a marked outward rectification when recorded in the presence of physiological concentrations of divalent cations.^978^^,^^991^ Depending on the applied activator, TRPA1 inward currents exhibit a slight and variable 2- to 8-fold preference for divalent versus monovalent cations.^1000^^,^^1001^ Likewise, unitary current amplitudes strongly depend on the composition of bath and pipette solutions and the applied agonists. In isotonic solutions, they range between 48 pS for inward currents measured in the presence^1000^ of 5 mM intracellular Mg^2+^ and 251 pS in symmetrical Cs^+^ solutions^978^ containing only 0.1 mM Mg^2+^. The smaller, nonflickering unitary currents in the presence of divalent cations that can permeate through the TRPA1 pore are consistent with the assumption that divalent cations act as permeating blockers.^1002^ Applying charge carriers of different sizes, a pore diameter of at least 8.2 Å has been determined,^978^ and the pore may further dilate up to 13.8 Å when activated by mustard oil.^1000^ Accordingly,1003., 1004., 1005. TRPA1 prolonged activation by a variety of reactive or nonreactive agonists allows permeation of the organic cations Yo-Pro-1 or NMDG^+^, a phenomenon that has been attributed to pore dilation. Alternatively, pore dilation might be mimicked by changed intracellular ion concentrations.^434^

### C Expression pattern and primary physiological roles of TRPA1

Owing to its primary role as an irritant sensor, a strong expression of TRPA1 is found in sensory afferent neurons whose cell bodies are located in the dorsal root and trigeminal ganglia.^995^^,^^1006^ In these sensory neurons, TRPA1 expression strongly overlaps with that of TRPV1, qualifying them as nociceptors. Likewise, TRPA1 expression in enterochromaffin cells confers chemosensation and is coupled to the release of serotonin to regulate intestinal motility.^1007^ A moderate or low expression of TRPA1 is found in many other tissues and cell types, including various types of cancer, as recently reviewed,^1008^ and summarized in Table 1.

In general, chemosensation is governed by unpleasant smell, bitter taste, and pain, causing avoidance behaviors and controlling protective reflexes that limit exposure to potentially harmful substances. Being expressed in nociceptive neurons and cells that can confer cough or vomiting reflexes, TRPA1 is strategically positioned to recognize irritants and other xenobiotics, preventing further ingestion. Owing to species-dependent thermal TRPA1 activation, nocifensive responses may include the avoidance of potentially noxious hot or cold temperatures.

### D Human diseases associated with the TRPA1 channel

A rare disease-causing mutation in human TRPA1 gives rise to familial episodic pain syndrome (OMIM 615040). A single family with 21 affected members in 4 generations has been identified to carry an N885S missense mutation in S4, leading to a GOF variant of TRPA1 with 5-fold increased inward currents at normal resting potential, loss of outward rectification, and a shift in cinnamaldehyde (100 *μ*M)-induced, voltage-dependent activation of about 56 mV, causing higher channel activity at the physiological range of membrane potentials.^1009^ Epigenome-wide analyses of DNA methylation in twins with discordant sensitivity to heat-induced pain revealed that an increased pain sensitivity correlated with the demethylation of the TRPA1 promoter, possibly resulting in an enhanced channel expression in nociceptive neurons.^1010^

Besides the direct contributions of TRPA1 in pain perception and neuralgia, numerous physiological and pathophysiological responses have been described to involve TRPA1-mediated processes. A prominent theme is the role of TRPA1 in pulmonary and intestinal vagal afferents to sense irritants, thereby contributing to protective reflexes like cough and emesis or vomiting, but also in exaggerated responses, such as bronchial or visceral hypersensitivity, allergic responses, and chronic obstructive pulmonary disease (COPD), or its contribution to the development of various skin diseases, IR damage, migraine, and other forms of headache, as recently reviewed.^1011^^,^^1012^ TRPA1 activation in pulmonary fibroblasts has been shown to prevent the transition into myofibroblasts and may thus protect from the development of lung fibrosis.^1013^^,^^1014^ In addition, TRPA1 expression in the vascular endothelium may be exploited to treat cardiovascular diseases,^1015^ and aberrant TRPA1 expression in various malignancies has been proposed to enable cancer cells to sense and cope with conditions of oxidative stress.^1016^

### E Pharmacological modulators of the TRPA1 channel

#### 1 TRPA1-activating compounds

Besides the role of TRPA1 in direct or indirect responses to noxious cold or hot temperatures, TRPA1 may be regarded as a broadly specific irritant sensor. Owing to its unique sensitivity toward cysteine-modifying compounds and ROS, TRPA1 is activated by a plethora of chemical stimuli. They may be grouped into (1) pungent tastants and spices, (2) oxidants and phototoxic compounds, (3) approved drugs, and (4) specifically developed TRPA1-selective activators. Table 8^980^^,^^984^^,^^986^^,^1017., 1018., 1019., 1020., 1021., 1022., 1023., 1024., 1025., 1026., 1027., 1028., 1029., 1030., 1031., 1032., 1033., 1034., 1035., 1036., 1037., 1038., 1039., 1040. summarizes some of the most prominent TRPA1-activating compounds, as well as TRPA1 inhibitors or blockers.

**Table 8:** Pharmacological modulators of TRPA1.

Name (PubChem CID^a^)	Effect	References
JT010 (18524489)	Channel activation, EC_50_ = 0.65 nM	^1017^
PF-4840154 (53380803)	Channel activation, EC_50_ = 23 nM	^1018^
GNE551 (2135890)	Channel activation, EC_50_ = 254 nM	^980^
Dibenzoxazepine (9213)	Channel activation, EC_50_ = 63 nM	^1019^
Morphanthridine (10878016)	Channel activation, EC_50_ = 83 nM	^1019^
Acrolein (7847)	Channel activation, EC_50_ = 5 *μ*M	^1017^
Allyl isothiocyanate (5971)	Channel activation, EC_50_ = 11-64.5 *μ*M	^984^^,^^1020^
Allicin (65036)	Channel activation, EC_50_ = 7.5 *μ*M)	^1021^
Thymol (6989)	Channel activation, EC_50_ = 127 *μ*M	^1022^
Menthol (16666)	Channel activation, EC_50_ = 95 *μ*M	^1023^
Isoflurane (3763)	Channel activation, EC_50_ = 180 *μ*M	^1024^
Apomorphine (6005)	Channel activation, EC_50_ = 7.1 *μ*M	^1025^
Auranofin (16667669)	Channel activation, EC_50_ = 1 *μ*M	^1026^
Isovelleral (37839)	Channel activation, EC_50_ = 0.5 *μ*M	^1027^
Flufenamic acid (3371)	Channel activation, EC_50_ = 147 *μ*M	^1028^
Clopidogrel (60606)	Channel activation, EC_50_ = 5.4 *μ*M	^1029^
Ticlopidine (5472)	Channel activation, EC_50_ = 7.2 *μ*M	^1029^
Nicotine (89594)	Channel activation, EC_50_ = 17 *μ*M	^1020^
2-Iodoacetamide (3727)	Channel activation, EC_50_ = 357 *μ*M	^1030^
2-Methylsulfonothioyloxyethanamine (MTSEA) (53443082)	Channel activation, EC_50_ = 1.58 mM	^1030^
4-Hydroxynonenal (5283344)	Channel activation, EC_50_ = 13-27 *μ*M	^986^^,^^1031^
HC-030031 (1150897)	Channel inhibition, IC_50_ = 0.7-6.2 *μ*M	^1032^
A-967079 (42641861)	Channel inhibition, IC_50_ = 51 nM	^1033^
AP18 (9584673)	Channel inhibition, IC_50_ = 3.1 *μ*M	^1034^
LY3526318 (118961431)	Channel inhibition, IC_50_ = 13.5 nM	^1035^
AM-0902 (73297271)	Channel inhibition, IC_50_ = 131 nM	^1036^
BAY-390 (155539293)	Channel inhibition, IC_50_ = 16 nM	^1037^
GDC-0334 (122490062)	Channel inhibition, IC_50_ = 1.7 nM	^1038^
Ruthenium red (656819)	Channel block, IC_50_ < 10 *μ*M	^1032^^,^^1039^^,^^1040^

N/A, not available.

a PubChem Compound Identification number.

#### 2 Pungent tastants, spices, and natural products

Soon after the initial characterization of TRPA1, its activation by mustard oil, cinnamon oil, ginger, and others was recognized.^1006^^,^^1041^ Allicin, the spicy and unstable ingredient of garlic, strongly activates TRPA1, whereas its heat-derived conversion products, diallyl mono-, di-, and trisulfide, less strongly and/or less potently act on the channel.^1042^ Other natural compounds that cause TRPA1 opening include menthol, thymol, and nicotine.^1020^^,^^1022^^,^^1023^^,^^1043^ One should note that most compounds require concentrations of 10–300 *μ*M to elicit strong effects on TRPA1, and some of them (menthol, cinnamaldehyde, nicotine, and camphor) exert bimodal effects with a current inhibition when applied at even higher concentrations.^1023^^,^^1044^

#### 3 ROS, peroxidation products, and cysteine-modifying compounds

In chemosensory neurons, TRPA1 is a prominent molecular substrate that decodes ROS or chemical oxidants either directly or via the formation of peroxidation products of membrane lipids. Effective oxidants include hydrogen peroxide, hypochlorite,^1045^ and cysteine-modifying compounds, such as 2-methylsulfonothioyloxyethanamine or iodoacetamide.^1030^ While several cysteine-modifying compounds can covalently bind to TRPA1, ROS, ultraviolet light, or visible light in the presence of photosensitizing compounds^987^ are likely to act in an indirect fashion, eg, by peroxidation products of membrane lipids, such as 4-hydroxy-2-nonenal, 4-oxo-nonenal, and 4-hydroxyhexenal or oxidized phospholipids.^986^^,^^1031^^,^^1046^^,^^1047^

#### 4 Approved drugs

Since several FDA-approved drugs or drug metabolites are capable of activating TRPA1, stimulation of chemosensory neurons and vagal afferents may contribute to adverse responses to the respective drugs. Acrolein, an irritating and highly electrophilic metabolite of cyclophosphamide, activates the TRPA1 channel.^996^ The pungent smell of the TRPA1-activating volatile anesthetics isoflurane and desflurane limits their application during the induction of general anesthesia and may be linked to TRPA1 activation.^1024^ Stinging pain during photodynamic therapy may be caused by ROS that indirectly activate TRPA1.^1048^ Other TRPA1-activating drugs include several fenamates, apomorphine, auranofin, ticlopidine, and clopidogrel.^1025^^,^^1026^^,^^1028^^,^^1029^ In most cases, therapeutic plasma concentrations are much lower than the concentrations required to activate TRPA1. Nonetheless, high local concentrations of TRPA1-activating drugs may contribute to adverse gastrointestinal effects during oral application.

#### 5 Selective, highly potent, and photoswitchable TRPA1 activators

To gain a deeper insight into TRPA1-mediated functions and to further validate TRPA1 as a potential drug target, highly potent and selective TRPA1 activators are eagerly sought. Any kind of electrophilic activator would be highly prone to exerting off-target effects. Therefore, electrophilic TRPA1 activators such as JT010 may be regarded as second-generation tool compounds despite their increased potency.^1017^ The nonelectrophilic peptide TRPA1 activators, PF-4840154 or GNE551, are more drug-like and may provide some advantages as activating compounds in screening approaches.^980^^,^^1018^ Interestingly, GNE551, as well as a pain-inducing plasma membrane-permeable scorpion toxin WaTx, induce TRPA1 activation modes that differ from that induced by allyl isothiocyanate, with a lack of current inactivation and longer single-channel opening events, respectively.^1049^ By identifying TRPA1-activating compounds with reversibly photoswitchable azobenzene or azopyrazole moieties, optical control of zebrafish or human TRPA1 activity has been achieved.^1050^^,^^1051^

#### 6 TRPA1 inhibitors

Like in many other TRP channels, inward currents through TRPA1 are blocked by ruthenium red, a polycationic compound, which presumably plugs into the outer entrance of the pore and obliterates the permeation pathway in a voltage-dependent fashion.^1039^ The first TRPA1-selective inhibitor developed by Hydra Biosciences, HC-030031, acts in a voltage-independent fashion and has been demonstrated to counteract formalin-induced pain upon intraperitoneal application in rats.^1032^ Two inhibitory compounds, AP18 and A967079, share a styrene pharmacophore and are structurally related to the activating compound cinnamaldehyde. While the inhibitory potency of AP18 lies in the micromolar range, A967079 reaches a nanomolar potency, especially with regard to inhibition of human TRPA1.^1034^^,^^1052^

In a preclinical study, using a guinea pig model of chronic cough, intraperitoneally applied GRC 17536 (60–100 mg/kg) was more effective in suppressing citric acid-induced cough responses than dextrometorphan (50 mg/kg) as a comparator drug.^1053^ In the patent literature, more drug-like TRPA1-targeting compounds with low to mid-nanomolar inhibitory potency have been proposed by Bayer (WO2021233752A1), MSD (WO2011043954A1), Hofmann La-Roche (WO2019182925A1 and WO2018029288A1), Genentech (WO2018162607A1), Orion Pharma (WO2014053694), and Eli Lilly (WO2019152465A1), some of which share striking similarities with GRC 17536 or LY3526318. Bayer has decided to make one of these compounds, BAY-390, publicly available as an orally bioavailable and CNS-penetrant drug that inhibits rat and human TRPA1 with comparable potency and may facilitate further validation of TRPA1 as a potential pharmacological target in a variety of preclinical disease models.^1037^

### F Ongoing or completed clinical trials with the TRPA1 channel as a therapeutic target

Owing to its irritant-sensing properties, TRPA1 has gained considerable interest as a pharmacological target in diseased conditions to control symptoms such as pain, itch, cough, or neurogenic inflammation, including migraine.

Among the first completed phase 1 and 2 trials, Glenmark Pharmaceuticals Ltd has focused on the compound GRC 17536, a thienopyrimidinedione derivative, as either a systemically applied or inhaled TRPA1 antagonist. A combined phase 1/2 trial tested the safety, tolerability, and efficacy of inhaled GRC 17536 in patients with mild allergic asthma. While inhaled GRC 17536 met the safety endpoints in single and repeated application regimes, the drug failed to reach the primary efficacy endpoint to reduce the drop of the 1-second forced expiratory volume (FEV1) after an allergen challenge (Eudra CT: 2012-002567-99). In a cohort of elderly patients suffering from chronic cough, inhaled GRC 17536 again failed to prove effective (Eudra CT: 2013-002728-17). In a third study, GRC 17536 (250 mg) or placebo was administered twice daily orally for 28 days to diabetic patients suffering from peripheral neuropathic pain. After 4 weeks of treatment, a significant decline in the average pain intensity was not achieved as the primary endpoint for all participants in the subgroup with moderate to severe pain (Eudra-CT: 2012-002320-33). No other studies with GRC 17536 have since been reported, implying that further development of this candidate may have been stopped.

More recently, Eli Lilly has embarked on another series of clinical trials, aiming at validating the purin-based compound LY3526318 as a potential analgesic drug in osteoarthritis (NCT05080660), low back pain (NCT05086289), or diabetic neuropathy (NCT05177094). According to information provided via clinicaltrials.gov, all 3 studies have been completed in 2022. As of the time of writing, results remain undisclosed, and a possible progression into phase 3 trials has not been announced yet. In oropharyngeal dysphagia, various TRP channel activators have been probed for possible relief and reconstitution of safe swallowing. Notably, activators of TRPV1 (capsaicin and piperine) or TRPA1 (cinnamaldehyde and citral) provided the best results, highlighting a possible use of TRPA1-targeting sensory stimulants in deglutition disorders.^1054^

## VI TRPMLs

### A Introduction

The TRP channels TRPML1, TRPML2, and TRPML3 (also called MCOLN1–3 or mucolipin1–3) are Ca^2+^-permeable, nonselective cation channels expressed in early endosomes, late endosomes, recycling endosomes, and lysosomes. TRPMLs are like the other TRP channels, 6 TMD proteins. They form tetramers, with the channel pore between TMD5 and 6. Since 2016, numerous structures have been determined for all 3 TRPML members using either X-ray crystallography or cryo-EM (see “Domain topology, assembly, and functional characteristics of TRPMLs”).1055., 1056., 1057., 1058., 1059., 1060., 1061.

In contrast to TRPML1, which is ubiquitously expressed, TRPML2 is predominantly found in immune cells, while TRPML3 is found in immune cells, other specialized cells, such as melanocytes or hair cells of the inner ear, and endocrine glands and secretory cells, as recently shown by a whole-body analysis of TRPML3 expression in a GFP-reporter mouse model.^1062^ TRPMLs mediate cation flux from endosomes and lysosomes, sense endolysosomal pH, regulate membrane potential across endolysosomal membranes, regulate trafficking, exocytosis, endocytosis/phagocytosis, and autophagy in the endolysosomal system, and participate in lysosomal biogenesis, cell membrane repair, cell migration, and nutrient sensing (see “Expression pattern and primary physiological roles of TRPMLs”).

LOF mutations in the human TRPML1 gene (>50 deletions, point mutations, in-frame deletions, early stop mutations, and other types of mutations) cause the neurodegenerative lysosomal storage disorder mucolipidosis type IV (MLIV) in humans. MLIV is characterized by psychomotor abnormalities, corneal clouding, retinal degeneration, and achlorhydria, which results in an increase in blood gastrin levels, iron deficiency due to an absence of acid secretion in the stomach, and endolysosomal accumulation of macromolecules, lipids, and heavy metals like zinc and iron in endolysosomes throughout the body.1063., 1064., 1065., 1066., 1067., 1068. In human TRPML2, an SNP, which is common in certain African populations, results in the TRPML2 variant TRPML2^K370Q^. TRPML2^K370Q^ reportedly disrupts the ability of the channel protein to enhance viral infections, raising the possibility of altered susceptibility to certain viral infections in homozygous carriers of this and possibly other TRPML2 polymorphisms.^1069^ Mutations in TRPML3, TRPML3,^A419P^ and TRPML3^I362T/A419P^ are GOF mutations causing deafness and circling behavior in mice (Varitint-waddler mutants).1070., 1071., 1072., 1073., 1074. Equivalent mutations in the human isoform likewise result in strong GOF.^1070^ The discovery of humans homozygous for early stop codon variants of TRPML2 and TRPML3 (TRPML2^K329∗^ and TRPML3^R390∗^) argues that LOF of either TRPML2 or TRPML3 is not lethal.^1075^ Nothing is, however, known about the pathophysiological features or disease susceptibility of individuals carrying these mutations. In addition to the aforementioned pathologies, roles of TRPMLs in cancer, lung disease, cardiovascular and kidney disease, Alzheimer’s and Parkinson’s disease (PD), inflammation and immunity, osteoclast function and bone remodeling, muscular dystrophy, and intestinal pathology have been suggested (see “Human diseases associated with TRPMLs and mouse models”).

Endogenous activators of TRPMLs are the phosphoinositides phosphatidylinositol 3,5-bisphosphate (PI[3,5]P_2_) and phosphatidylinositol 3-phosphate (PI3P), the latter being a demonstrated^1076^ agonist of TRPML3, while PI(3,5)P_2_ activates all 3 isoforms. PIKfyve, a FYVE finger-containing phosphoinositide kinase, catalyzes the conversion from PI3P to PI(3,5)P_2_, the latter being predominantly found on late endosomal/lysosomal membranes, while PI3P is found on early endosomal and autophagosomal membranes.^1076^^,^^1077^ Besides the discovery of endogenous ligands, in over 10 years, a plethora of small-molecule agonists and a number of antagonists have been identified, mostly through high-throughput screenings, and were subsequently functionally characterized (see “Pharmacology of TRPMLs”).

TRPML1 as a drug target, specifically TRPML1 activation to treat, eg, lysosomal storage disorders and other neurodegenerative diseases, has gained much attention in recent years. Thus, according to publicly available information, Calporta Therapeutics, acquired by Merck in 2019, has developed preclinical stage TRPML1 agonists for potential treatment of Niemann-Pick C disease (NPC) and other lysosomal storage diseases, as well as amyotrophic lateral sclerosis, Alzheimer’s disease, and PD. Caraway Therapeutics, bought by Merck in 2023, has developed, with support from the Michael J. Fox Foundation, TRPML1 agonists for GBA-PD treatment. And Casma Therapeutics has likewise developed TRPML1 agonists according to the Alzheimer’s Drug Discovery Foundation (see “TRPMLs as therapeutic targets”).

In sum, we will discuss here the current knowledge of TRPMLs from structural aspects to function and physiology, including pathophysiology, and potential therapeutic applications, including currently available pharmacological tools to modulate TRPML channel activity.

### B Domain topology, assembly, and functional characteristics of TRPMLs

**Fig. 6:**
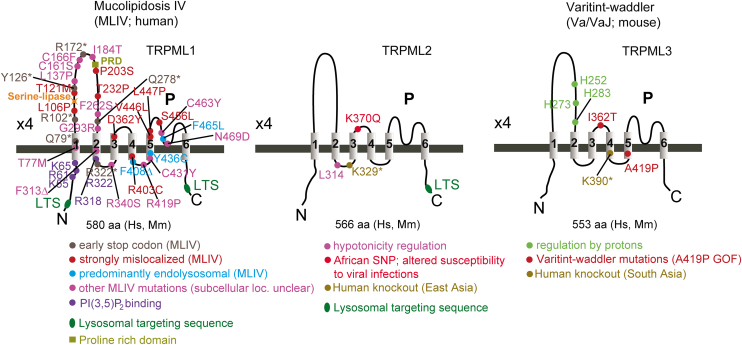
Shown as schematic are the monomeric structures of the 3 TRPMLs. In humans, TRPML1 > 50 MLIV, causing homozygous mutations or heterozygous combinations of mutations have been identified in Jewish Ashkenazi (AJ) as well as non-Jewish (NJ) populations in the USA, Canada, France, Germany, the Middle East, India, Japan, and other countries. The most common mutation is MCOLN1^IVS3-2A>G^ (AJ major; not shown in the cartoon). Others comprise single point mutations (in red, pink, and blue, respectively), in-frame deletions (F408Δ), and early stop mutations (Q79X, R102X, Y126X, R172X, Q278X, and R322X). In addition, small deletions or insertions leading to frame shifts, splicing mutations, and complex rearrangements can also occur (not shown in the cartoon; see Krogsaeter et al.^1080^). PRD = proline-rich domain, LTS = lysosomal targeting sequence. Hs = Homo sapiens; Mm = Mus musculus. Point mutations shown in blue are predominantly expressed in lysosomes, while the ones depicted in red are heavily mislocalized.^1067^ The subcellular localization of the mutations shown in pink has not been systematically analysed yet. TRPML2 is the only osmo-/mechanosensitive TRPML channel.^1081^ Amino acid L314 within the PI(3,5)P_2_ binding pocket abrogates osmo-/mechanosensation while activation by the TRPML2 selective agonist ML2-SA1 is unaffected.^1082^ TRPML2^K370Q^ disrupts the ability of the channel protein to enhance viral infections, raising the possibility of altered susceptibility to certain viral infections in homozygous carriers of this and possibly other TRPML2 polymorphisms.^1069^ In mouse TRPML3, the mutations A419P (Va) and A419/P/I362T (VaJ) are GOF mutations causing the Varitint-waddler phenotype characterized by circling behavior, deafness, and coat color dilution.1070., 1071., 1072., 1073., 1074.^,^^1083^^,^^1084^ TRPML3^A419P^ and TRPML3^A419P/1362T^ are likewise GOF variants in human.^1070^ Amino acids H252, H272, and H283 are involved in pH sensing and pH-dependent channel activity regulation (increasing pH increases activity). In both human TRPML2 and TRPML3 viable putative KO or LOF variants in humans have been identified (TRPML2^K329∗^ and TRPML3^K390∗^).^1075^

TRPMLs have, like other TRP channels, long been postulated to comprise 6 TMDs with a pore (P) loop between TMD5 and 6, and the functional pore being formed by tetrameric assembly.^1078^ Structural evidence available since 2016 eventually confirmed these predictions.1055., 1056., 1057., 1058., 1059., 1060., 1061. The TRPMLs together with the TRPPs differ from the rest of the TRP channels due to the presence of a large extracellular/luminal loop between TMD1 and 2. Structural analysis revealed that the 4 luminal linker domains form a square-shaped canopy with a central opening above the channel pore.^1055^ The canopy in TRPMLs forms a cap-like structure and acts as a highly negative electrostatic trap or sink, which facilitates ion selection by favourably attracting divalent Ca^2+^ ions, limiting the access of monovalent cations to the filter, thereby reducing the permeation of monovalent ions.^1060^ All TRPMLs are activated by PI(3,5)P_2_, and several amino acids have been identified either by functional assays (eg, endolysosomal patch-clamp) or in structural studies to affect PI(3,5)P_2_ binding, eg, K55, R61, K65, R318, and R322 in TRPML1 (Fig. 6).^1055^^,^^1057^^,^^1067^^,^1069., 1070., 1071., 1072., 1073., 1074., 1075.^,^1079., 1080., 1081., 1082., 1083., 1084. Two additional amino acids in TRPML1, Y355 and R403 were postulated to be involved in PI(3,5)P_2_ activation. Thus, the phosphate group of PI(3,5)P_2_ induces Y355 to form a *π*-cation interaction with R403, moving the TMD4–5 linker, resulting in an allosteric activation of the channel.^1079^

**Fig. 7:**
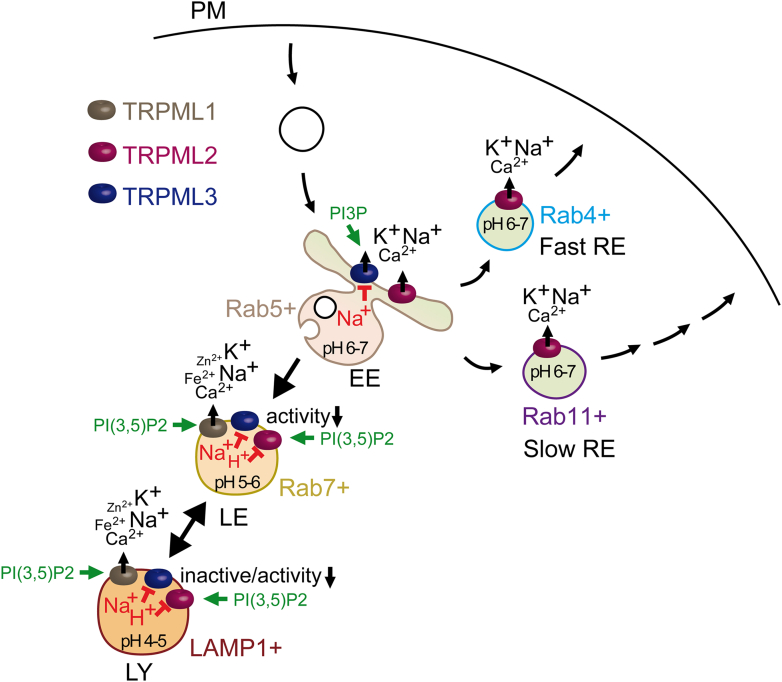
Schematic showing intracellular distribution/expression of TRPMLs and putative endogenous activation and inhibition mechanisms. All 3 TRPMLs are activated by PI(3,5)P_2_ (LE/LY); TRPML3 was also shown to be activated by PI3P (EE). TRPML3 is blocked by high luminal Na^+^ and H^+^, while TRPML2 is blocked by H^+^. EE, early endosomes; LE, late endosomes; LY, lysosomes; RE, recycling endosomes.

Notably, R403K is an MLIV-causing mutation in human TRPML1 (Fig. 6) that cannot be significantly activated by PI(3,5)P_2_ anymore. By contrast, the small-molecule agonists ML-SA1 and MK6-83 (Table 9)^1062^^,^^1065^^,^^1067^^,^^1069^^,^^1070^^,^1080., 1081., 1082.^,^1085., 1086., 1087., 1088., 1089., 1090., 1091., 1092., 1093., 1094., 1095., 1096., 1097., 1098., 1099., 1100., 1101., 1102., 1103. can still activate this mutant, albeit to a much lesser extent than WT.^1067^^,^^1079^ In humans, >50 MLIV caused by homozygous TRPML1 mutations or heterozygous combinations of mutations has been identified. The most common mutation is MCOLN1^IVS3-2A>G^, a splicing mutation (AJ major),^1104^^,^^1105^ followed by AJ minor (MCOLN1^511del6434^ or MCOLN1^Ex1-7del^), both resulting in the loss of a functional gene product. Other mutations comprise single point mutations (either correctly localized in lysosomes (blue), substantially mislocalized (red), or of unknown subcellular localization; marked in pink), in-frame deletions (F313Δ, F408Δ), and early stop mutations (Q79∗, R102∗, Y126∗, R172∗, Q278∗, and R322∗). In addition, small deletions or insertions leading to frame shifts, splicing mutations and complex rearrangements can also occur (see Krogsaeter et al^1080^; “Expression pattern and primary physiological roles of TRPMLs”; Fig. 6). While most of the point mutations are severely mislocalized, some retain their lysosomal localization and at least residual activity,^1067^ eg, F408Δ and F465L. Y436C, however, despite being correctly expressed in lysosomes, was shown^1067^ to be neither activated by PI(3,5)P_2_ nor the synthetic agonist MK6-83. This is in accordance with data provided by Schmiege et al,^1059^ demonstrating that Y436A is not activatable by the synthetic ligand ML-SA1, as this amino acid, Y436, together with I468, F428, C429, C432, Y436, F465, F505, F513, Y499, and Y507 forms a hydrophobic cavity accommodating the agonist ML-SA1. Like Y436A, F465A reportedly cannot be activated by ML-SA1 in whole-cell patch clamp experiments using a plasma membrane redirected TRPML1 channel.^1059^ By contrast, Chen et al^1067^ reported that F465L can still be activated with the synthetic small-molecule agonist of TRPML1, MK6-83, in endolysosomal patch-clamp experiments. Furthermore, Chen et al^1067^ found that mutation F465L has lost its pH sensitivity, ie, pH 4.6, in the lysosomal lumen and cannot further increase channel activity compared with pH 7.2, as seen typically in WT TRPML1.^1067^^,^^1096^ In contrast to TRPML1, which shows increasing activity with decreasing (ie, more acidic) luminal pH, TRPML3 activity increases with increasing pH, ie, from luminal 4.6 to 6.8 or 7.2 (Fig. 7).^1084^ Since a pH of 6.8 is common in early endosomes, TRPML3 would naturally be more active in early rather than late endosomes or lysosomes. Indeed, functionally TRPML3 seems largely silent in endogenous acidic lysosomes and only becomes active if the luminal pH of the lysosomes rises, eg, under pathogenic conditions (see Miao et al^1106^ and “Expression pattern and primary physiological roles of TRPMLs”).

**Table 9:** Summary of TRPML channel characteristics

Name	TRPML1	TRPML2	TRPML3
Synonyms	MCOLN1, Mucolipin1	MCOLN2, Mucolipin2	MCOLN3, Mucolipin3
Length (aa) Hs	580	566	553
Length (aa) Mm	580	566 (538; isoform 2)	553
Seq motifs	Serine lipase; Lysosomal targeting seq. (N- and C-terminal); Proline rich domain (PRD)	Lysosomal targeting seq. (LTS; N-terminal in Mm)	N.D.
Localization	Late endosomes (LE)/lysosomes (LY); LRO?	Rab4+ and Rab11+ recycling endosomes (RE); early endosomes (EE)^1081^^,^^1082^; LE/LY^1082^; LRO?; PM (in-vitro/OE)	EE; LE/LY; not in RE; melanosomes? PM (in-vitro/OE); phagophore during autophagy
Tissue distribution	Ubiquitous	Thymus, spleen, kidney, trachea, liver, lung, colon, testis, thyroid, B- and T-cells, macrophages, dendritic cells	Hair cells of the inner ear, organ of corti, utricle, stria vascularis, lung (alveolar macrophages), (skin) melanocytes, (neonatal) enterocytes, kidney, lung, olfactory bulb (sensory neurons), nasal cavity, thymus, colon, trachea, several glands (parathyroid, salivary, adrenal, pituitary), testes, ovaries^1062^^,^^1070^^,^1085., 1086., 1087.
Activators	PI(3,5)P_2_ (endogenous) ML1-SA1 (EVP-169) = isoform selective^1088^ Others: ML-SA1 (not isoform selective)^1089^ SF-22, SF-51 (not isoform selective)^1089^ MK6-83 (EC_50_, 0.11 *μ*M (patch-clamp))^1067^ ML-SA3 (isoform selectivity unclear), ML-SA5 (isoform selectivity unclear)^1090^ Rapamycin^1091^ NAADP?	PI(3,5)P_2_ (endogenous) ML2-SA1 (EVP-22) = isoform selective (EC_50_, 1.2 *μ*M (Ca^2+^ imaging))^1082^ Others: ML-SA1 (not isoform selective) SF-21; SF-41; SF-81 (not isoform selective)^1065^ Rapamycin^1091^ (+)-trans-ML-SI3 = TRPML2 agonist (see section on inhibitors)	PI3P, PI(3,5)P_2_ (endogenous) ML3-SA1 (EVP-77; mouse isoform selective; EC_50_, 9 *μ*M (Ca^2+^ imaging)^1088^ EVP-21 (human isoform selective; EC_50_, 4.3 μM (Ca^2+^ imaging)^1092^ Others: ML-SA1 (not isoform selective) SF-11; SN-1; SF-21; SF-22; SF-31; SF-23; SF-41; SF-51; SF-32; SF-24; SF-33; SN-2; SF61; SF-71; SF-81^1085^
Inhibitors	PIP_2_ (endogenous) ML-SI1 (not isoform selective; stereochemistry of the active isomer not elucidated; dependent on activator) ML-SI2 (structure not published) (–/+)-trans-ML-SI3 (not isoform selective)^1093^; racemic trans-isomer commercially available; both enantiomers available by enantioselective synthesis^1094^ EDME (isoform-selective; IC_50_, 0.6 *μ*M (Ca^2+^ imaging) and 0.2 *μ*M (patch-clamp))^1095^ PRU-10, PRU-12 (EDME derivatives; isoform selective; IC_50_, 0.4 and 0.3 *μ*M (Ca^2+^ imaging))^1095^ High luminal pH Sphingomyelins (SMs)	PIP_2_ (endogenous) ML-SI1, (–)-trans-ML-SI3 (not isoform selective; (+)-trans-ML-SI3 = TRPML2 agonist!)^1093^ Low luminal pH	P)P_2_ (endogenous) (–)-trans-ML-SI3 (effect weaker than for TRPML1 and TRPML2)^1093^ No other synthetic small molecule blockers currently available Low luminal pH and high luminal Na^+^
Regulators	Acidic luminal pH increases activity^1067^^,^^1096^	Acidic luminal pH reduces activity^1082^	Low luminal Na^+^ potentiates activation Acidic luminal pH reduces activity^1097^
Disease mutations or polymorphisms associated with a phenotype	MLIV is associated with mutations in HsTRPML1; symptoms include severe psychomotor retardation, retinal degeneration, corneal clouding, achlorhydria, elevated serum gastrin levels, iron deficiency, (lipid) storage bodies in almost every cell type (>50 MLIV causing homozygous mutations or heterozygous combinations of mutations identified)	TRPML2^K370Q^ disrupts the ability of the channel protein to enhance viral infections, raising the possibility of altered susceptibility to certain viral infections in homozygous carriers of this and possibly other TRPML2 polymorphisms^1069^	Deafness, circling behavior, head bobbing and coat color dilution is associated with mutations in MmTRPML3 (Varitint-waddler mutations TRPML3^A419P^ (Va) and TRPML3^A419P/I362T^ (VaJ))
(Disease-associated) GOF mutants	V432P (Hs, Mm)	A425P (Hs); A396P (Mm, isoform 2)	Va (A419P) and VaJ (A419P/I362T) (Hs, Mm)
(Disease-associated) LOF mutants	IVS3-2A>G (AJ major), Ex1-7del (AJ minor), T77M, Q79∗, R102∗, L106P, T121M, Y126∗, L137P, C161S, C166F, R172∗, I184T, P203S, T232P, F262S, Q278∗, G293R, F313Δ, R322∗, R340S, D362Y, R403C, F408Δ, R419P, C431Y, Y436C, V446L, L447P, S456L, C463Y, F465L, N469D, small deletions or insertions leading to frame shifts, splicing mutations and complex rearrangements (see also Krogsaeter et al.^1080^ and Fig. 6)	K329∗ (homozygous; Hs)	K390∗ (homozygous; Hs)
KO mouse models	KO mice display enlarged vacuoles, psychomotor defects, retinal degeneration, impairments in basal and histamine-stimulated gastric acid secretion,^1098^ impaired myelination and reduced brain ferric iron,^1099^ early-onset muscular dystrophy^1100^ TRPML1/3 co-deficiency causes accelerated endolysosomal vacuolation of enterocytes and failure-to-thrive from birth to weaning^1101^	KO mice display defects in inflammatory mediator release, in particular CCL2 (MCP-1)^1082^^,^^1102^	KO mice display no auditory or vestibular phenotype and no coat color dilution^1086^; Two different KO mouse models (Mcoln3^tm1.2Hels^ and Mcoln3^tm1.1Jga^) show an increased susceptibility to develop emphysema/COPD and increased MMP12 levels in broncho-alveolar fluid and in the supernatant of cultured alveolar macrophages^1088^ TRPML1/3 co-deficiency causes accelerated endolysosomal vacuolation of enterocytes and failure-to-thrive from birth to weaning^1101^
Functions	Lysosomal exocytosis; regulates autophagy (TFEB, calcineurin, CaMKK*β*/VPS34); role in sorting/transport in late endocytic pathway; regulation of lysosomal lipid and cholesterol trafficking; ROS sensor in lysosomes; endolysosomal cation/heavy metal (iron, zinc) homeostasis; role in gastric acid secretion; regulation of lysosomal motility; plasma membrane repair; phagocytosis; endolysosomal pH regulation?; vesicle fusion, fission?; NAADP receptor?	Osmo-/mechanosensation in RE; EE/RE trafficking; endolysosomal cation homeostasis; vesicle fusion, fission?; endolysosomal pH regulation?	Endocytosis, macropinocytosis (MMPs); regulates autophagy; EE trafficking; endo-lysosomal cation homeostasis; senses lysosome neutralization by pathogens to trigger their expulsion; vesicle fusion, fission? endolysosomal pH regulation?
Interacting proteins	ALG2^1103^; TRPML2, TRPML3, TPC1?, TPC2?; LAPTMs; Hsp40; Hsc70	TRPML1, TRPML3, Hsc70?	TRPML1, TRPML2, GATE16, TPC1?, TPC2?, Hsc70?

A further feature of TRPML3 is its sensitivity to high luminal Na^+^ levels (Fig. 7). The lower the Na^+^ concentration in the lumen of TRPML3-expressing endolysosomes, the higher its activity. This effect seems to be dependent on E361, as mutation E361A has an increased activity in high Na^+^-containing luminal solution.^1065^ While E361 is located in the luminal loop between TMD3 and 4, amino acids H252, H272, and H283, which are involved in pH sensing and pH-dependent TRPML3 activity regulation, are located in the large luminal loop between TMD1 and 2. The effect of luminal pH on TRPML2 is similar to that of TRPML3. TRPML2 activity, stimulated with either PI(3,5)P_2_ or the TRPML2 isoform-selective agonist ML2-SA1, increases with increasing (ie, less acidic) luminal pH.^1082^ This is in accordance with TRPML2 being also expressed in less acidic vesicles, in particular Rab11+ and Rab4+ recycling endosomes (Table 9),^1082^ while the expression of TRPML1 is strictly limited to late endosomes/lysosomes. A special feature of TRPML2, in contrast to TRPML1 and TRPML3, is its sensitivity to osmo-/mechano-stimulation.^1081^ The point mutation L314R within the TRPML2 PI(3,5)P_2_ binding pocket abolishes osmo-/mechanosensitivity and slows down the fast recycling pathway, while activation with ML2-SA1 is preserved. Introducing leucine residues at positions corresponding to L314 in TRPML1 (ie, R322L) or TRPML3 (ie, R309L) was not sufficient to induce osmo-/mechanosensitivity in TRPML1 or TRPML3.^1081^ A role of TRPML2 in fast recycling processes and the secretion of inflammatory mediators, such as CCL2 (MCP-1), has been postulated.^1082^^,^^1102^

In contrast to TRPML2, for both TRPML1 and TRPML3, roles in autophagy have been demonstrated.^1076^^,^^1107^^,^^1108^ Medina et al^1107^ showed that TRPML1-mediated Ca^2+^ signaling regulates autophagy through transcription factor EB (TFEB) and calcineurin. TFEB is a well established master gene for lysosomal biogenesis, driving the expression of autophagy and lysosomal genes.^1109^ TRPML1 controls both TFEB activity and TFEB downstream transcriptional targets.^1107^^,^^1110^ In addition, Scotto Rosato et al^1110^ showed that during starvation, TRPML1 links lysosomal Ca^2+^ release to autophagosome biogenesis through the activation of the CaMKK*β*/VPS34 pathway, a mechanism which is independent of the TFEB transcriptional program and involves activation of the CaMKK*β* and the AMP-activated protein kinase AMPK. Furthermore, ROS can induce autophagy via TRPML1. An increase in mitochondrial ROS levels or exogenous oxidants directly stimulates TRPML1-mediated Ca^2+^ release from lysosomes, thus triggering calcineurin-dependent TFEB nuclear translocation.^1107^^,^^1111^ Furthermore, a role for TRPML1 in the fusion of autophagic vesicles with lysosomes has been postulated.^1112^^,^^1113^ On the other hand, Cao et al^1114^ claimed that TRPML1 is required for the recovery of enlarged lysosomes and proposed a role for TRPML1 in lysosomal fission. Despite these controversies, the role of TRPML1 in autophagy modulation via multiple pathways is well established.

Less evidence is available for the exact role of TRPML3 in autophagy, but recent results by Kim et al^1076^ showed that TRPML3 activation increases autophagy while TRPML3 inhibition suppresses it. Kim et al^1076^ identified PI3P as a physiological activator of TRPML3 to release Ca^2+^ from the phagophore, thus promoting autophagy. The possibility that TRPML3 modulates autophagy independently from TRPML1 gained considerable momentum with these new results.

Besides being an autophagy regulator, regulation of lysosomal exocytosis is another well established role of TRPML1.^1100^^,^1115., 1116., 1117. Upon TRPML1 stimulation, lysosomes move to the plasma membrane (visualized, eg, by LAMP1 translocation). Lysosomes can fuse directly with the plasma membrane and release their content into the extracellular space. Increase in local Ca^2+^ seems to trigger the fusion process,^1118^^,^^1119^ and the concept that the lysosome releases Ca^2+^ by itself via TRPML1 for this process has gained much interest.^1096^^,^^1100^^,^^1115^^,^^1116^^,^1120., 1121., 1122., 1123., 1124. Lysosomes containing GOF mutants of TRPML1 can also undergo uncontrolled lysosomal exocytosis.^1125^ By contrast, in TRPML1 KO mice, Park et al^1126^ found that fusion of lysosomes with secretory organelles leads to uncontrolled exocytosis. How these findings can be reconciled with the accumulating evidence that TRPML1 is a positive regulator of lysosomal exocytosis remains unresolved.

There is also evidence for a role of TRPML3 in lysosomal exocytosis under certain conditions, ie, when the lumen of the lysosome gets neutralized and TRPML3 becomes more active. Miao et al^1106^ showed that TRPML3 is required for bacterial expulsion, specifically for uropathogenic *Escherichia coli* (UPEC) release from infected cells, through lysosomal exocytosis. UPEC, after infecting bladder epithelial cells, are targeted for degradation by the autophagic machinery. However, UPEC escapes degradation by increasing the lysosomal pH. TRPML3 activity is triggered by an increase in pH, and UPEC can be exocytosed; nevertheless, it is also a potentially important protective mechanism in other infectious diseases.

### C Expression pattern and primary physiological roles of TRPMLs

TRPML1 is ubiquitously expressed. TRPML2 is predominantly found in immune cells, including macrophages, dendritic cells, and B and T cells (for recent reviews see Chao et al^1127^ and Spix et al^1128^; Table 9). TRPML3 is likewise found to be expressed in immune cells, eg, in alveolar macrophages in the lung (Table 9).^1087^ TRPML3 is also found in skin and inner ear melanocytes, hair cells of the inner ear, olfactory sensory neurons, principal cells of the collecting duct in the kidney, and in endocrine glands and secretory cells, as recently shown by a whole-body analysis of TRPML3 expression in a GFP-reporter mouse model (Table 9).^1062^^,^^1129^ Thus, TRPML3 was found to be expressed in the adenohypophysis of the pituitary gland, the cortex of the adrenal gland, the parathyroid gland (presumably in chief cells), and testes (presumably in spermatozoa). For comparison with human expression data, please refer to Grimm et al^1129^ or the Human Protein Atlas.^452^

### D Human diseases associated with TRPMLs and mouse models

LOF mutations in TRPML1 lead to progressive neurodegeneration in humans, as discussed above. Mutations also affect the eye and stomach. Patients suffer, eg, from corneal clouding, retinal degeneration, achlorhydria, and iron deficiency resulting from an absence of acid secretion in the stomach. However, TRPML1 is ubiquitously expressed, and endolysosomal accumulation of macromolecules, lipids, heavy metal ions like Zn^2+^ and Fe^2+^, and probably others in endolysosomes throughout the body of MLIV patients must be assumed.1063., 1064., 1065., 1066., 1067., 1068. In addition to brain defects, potential defects due to lack or dysfunction of TRPML1 in the kidney have been proposed.1130., 1131., 1132., 1133., 1134. Other organs may also be affected, eg, liver, as MLIV patient fibroblasts were shown to accumulate cholesterol^1117^ or spleen, where loss of TRPML1 results in defective red blood cell clearance by macrophages.^1115^

MLIV goes along with a strongly reduced quality of life and overall life expectancy. Currently, no curative treatment is available, although treatment of several LOF mutations with small-molecule TRPML1 agonists has been proposed.^1067^ Most promising candidates for such an approach would be patients with mutations in TRPML1 that do not result in strong mislocalization of the protein or mutations that have a loss of PI(3,5)P_2_ sensitivity, while synthetic small-molecule agonists are still able to activate the channel, with the prerequisite that they must retain some lysosomal localization.

Another proposed strategy is the replacement of the TRPML1 function by enhancing the activity of an alternative Ca^2+^-permeable cation channel in endolysosomes, eg, 2-pore channel 2 (TPC2; see Prat Castro et al^1135^ and Scotto Rosato et al^1117^). TRPML1 is permeable for Na^+^, K^+^, and Ca^2+^ but also for Zn^2+^, Fe^2+^, and other metal ions. While activation by TPC2 could potentially rescue the functions associated with Na^+^/Ca^2+^ permeability, the accumulation of metal ions in the lysosomal lumen, such as Zn^2+^ or Fe^2+^, may require additional strategies. While TRPML1 LOF mutations or KO result in severe disease in human and mouse (the murine MLIV phenotype is very similar to the human phenotype),^1099^^,^^1136^^,^^1137^ no disease-causing LOF or KO phenotypes for TRPML2 or TRPML3 are known in humans. Apparently, homozygous mutations in TRPML2 and TRPML3, resulting in an early stop (TRPML2^K329∗^ and TRPML3^R390∗^) in humans, are not lethal (Table 9).^1075^ Early stop mutations or complete loss of TRPML1 in humans are likewise not lethal, but result in severe phenotypes. Whether homozygous carriers of TRPML2^K329∗^ and TRPML3,^R390∗^ identified in East and South Asia, are severely ill or have other health disadvantages, is not known. Several heterozygous carriers were also identified in North Borneo (Dusun people).

A homozygous mutation in TRPML2, common in certain African populations, is TRPML2^K370Q^. TRPML2^K370Q^ reportedly disrupts the ability of the channel protein to enhance viral infections, raising the possibility of an altered susceptibility to certain viral infections in homozygous carriers of this and possibly other TRPML2 polymorphisms.^1069^

Disease-causing mutations for TRPML3 have been described only in mice. The GOF mutations, A419P (Va) and A419/P/I362T (VaJ), cause the Varitint-waddler phenotype characterized by circling behavior, deafness, and coat color dilution.1070., 1071., 1072., 1073., 1074.^,^^1083^^,^^1084^ Introducing A419P or A419P/I362T mutations into WT human TRPML3 likewise result in GOF effects.^1070^ The Varitint-waddler phenotype can be rescued by overexpression of plasma membrane ATPase, suggesting that cytosolic Ca^2+^ overload due to the TRPML3 GOF mutation A419P is causative for the observed effects.^1074^ The additional mutation of I362T in VaJ results in a slightly less severe phenotype, which nevertheless shows similar Ca^2+^ overload as well as circling behavior, deafness, and coat color dilution.^1070^^,^^1083^ The reason for VaJ being milder remains unclear. Of note, however, I362T is located next to E361, which was reported to affect the Na^+^ sensitivity of TRPML3, increasing channel activity.^1065^

Besides diseases associated directly with mutations in TRPMLs, KO mouse models and other functional studies have revealed additional potential roles of TRPMLs in physiology and pathophysiology, from immune cell function and cancer to gastrointestinal, kidney, cardiovascular, neurodegenerative, lung, and infectious diseases.

TRPML1 activity is strongly reduced with increasing pH; hence, a backup channel getting engaged when TRPML1 is blocked, eg, as in the case of bladder epithelial cell infection with UPEC, which neutralize the lysosomal lumen to evade degradation, seems an elegant solution.^1106^ TRPML3 can fulfill that function as it gets activated when the pH in the lysosomal lumen increases, while under normal lysosomal pH conditions, it would be largely silent. Other indications for roles of TRPMLs in infectious diseases relate to TRPML1, but increasingly also to TRPML2 and TRPML3. Thus, TRPML1 was shown to play a role in *Helicobacter pylori* infection,^1138^ where virulence factor vacuolating cytotoxin A promotes more severe disease development and gastric colonization. Virulence factor vacuolating cytotoxin A targets TRPML1 to disrupt endolysosomal trafficking and autophagy, an effect that could be reversed by direct activation of TRPML1 with a small-molecule agonist, leading to the clearance of intracellular bacteria. Furthermore, Khan et al^1139^ reported on the role of TRPML1 in cooperation with the big-conductance Ca^2+^ activated K^+^ channel (BK) in HIV infection and proposed that TRPML1/BK coactivation leads to an enhanced acidification of endolysosomes, resulting in an increased degradation of Tat protein, which facilitates HIV replication. TRPML2 was postulated to enhance viral infections, eg, yellow fever virus, influenza A virus, and equine arteritis virus infections, and the human TRPML2 variant, TRPML2^K370Q^, discussed before, reportedly shows a LOF phenotype with respect to viral enhancement.^1069^ Quite to the contrary, Gibbs et al^1140^ found that TRPML2 acts also as an inward rectifying Mg^2+^ channel on endolysosomes and can thus deprive *Salmonella Typhi* of Mg^2+^, restricting growth.

Xu et al. recently showed that suppressing either TRPML3 or BK helps bacterial survival, whereas increasing either TRPML3 or BK favors bacterial clearance.^1141^

Hence, in sum, it is currently claimed that activation of TRPML1 and TRPML3 would be beneficial in treating certain infectious diseases, while in the case of TRPML2, it remains unclear whether activation or inhibition may be more beneficial. This may, of course, also depend on the type of infection.

Generally, all 3 TRPMLs are expressed in a range of immune cells, including different types of macrophages, natural killer cells, dendritic cells, B and T cells, microglia, and astrocytes (Table 9).^1127^^,^^1128^^,^1142., 1143., 1144. In the mouse lung, TRPML3 is almost exclusively expressed in alveolar macrophages^1088^ and in 2 independent KO mouse models (Mcoln3^tm1.2Hels^ and Mcoln3^tm1.1Jga^), an increased susceptibility to develop an emphysema-like phenotype was found. KO mice showed differences in lung function and histological parameters such as elastance and compliance, or the mean linear chord length (mean free distance in the air spaces), pointing to an emphysema-like phenotype compared with WT mice, which was further and more strongly exacerbated in KO mice compared with WT mice after elastase or tobacco smoke treatment. In broncho-alveolar fluid and the supernatant of cultured alveolar macrophages, increased levels of matrix metalloproteinase 12 (macrophage elastase) were detected, a known risk factor for emphysema and COPD development.^1088^^,^^1145^ The authors further found that the relative TRPML3 expression was increased in samples from human smokers with COPD compared with healthy smokers. TRPML3 expression was also increased in smokers compared with nonsmokers. This was interpreted as being a potential compensatory mechanism to increase the uptake of excess matrix metalloproteinase 12 and potentially other MMPs with the help of TRPML3.

TRPML1, in addition, was shown to play an important role in the gastrointestinal tract. Thus, Sahoo et al^1068^ found that TRPML1 overexpression or activation in mouse parietal cells induced gastric acid secretion, while TRPML1 inhibitors blocked it. This is in accordance with human MLIV patients who are reportedly achlorhydric. Mechanistically, TRPML1 was found to play a role in gastric acid secretion in parietal cells by regulating the trafficking and exocytosis of H^+^/K^+^-ATPase-rich tubulovesicles after histamine stimulation.^1068^ Chandra et al^1098^ found that Trpml1 KO mice have significant impairments in basal and histamine-stimulated gastric acid secretion.

There are also reports of progressive renal failure in MLIV patients, and blockade of TRPML1 was found to suppress the interaction of lysosomes and multivesicular bodies, leading to increased exosome release from mouse podocytes.^1146^ In addition, Nakamura et al^1130^ suggested a role of lipidated LC3 interacting with TRPML1 to release lysosomal Ca^2+^ essential for TFEB activation during kidney injury and lysosomal damage response.^1133^

In 2 recent works published by the same group,^1147^^,^^1148^ it is claimed that inhibition of TRPML1 has a protective role in myocardial ischemia/reperfusion injury. Mechanistically, this was attributed to a restoration of impaired cardiomyocyte autophagy by blocking TRPML1, which gets activated by ROS elevation, following myocardial ischemia/reperfusion injury. Activated TRPML1, releasing lysosomal Zn^2+^, reportedly blocks autophagic flux in cardiomyocytes by disrupting the fusion between autophagosomes and lysosomes. This is a surprising finding, as TRPML1, according to the vast majority of publications, is believed to promote autophagy rather than inhibit it. Thus, a large body of evidence suggests that TRPML1 promotes autophagy through activation of TFEB, mediated by lysosomal Ca^2+^ release.^1107^^,^^1108^^,^^1111^^,^^1112^^,^^1149^^,^^1150^ TRPML1 is also much less permeable to Zn^2+^ compared with Ca^2+^, hence a dominant effect of Ca^2+^ on autophagy would be expected.

Several lines of evidence point to a role of TRPMLs in different types of cancer, eg, breast cancer, melanoma, or glioma^1095^^,^1151., 1152., 1153., 1154., 1155., 1156., 1157., 1158. (for recent reviews, see ^1124^^,^^1153^^,^1159., 1160., 1161.). The loss or inhibition of TRPMLs reduces, eg, cancer cell migration and invasion, and roles of TRPMLs in TFEB-mediated gene transcription and lysosomal exocytosis promoting invasiveness and drug resistance in cancer cells, cancer cell nutrient sensing, and antitumor immunity have been proposed. Collectively, the data suggest that TRPMLs, in particular TRPML1, stimulate oncogenesis by enhancing survival, growth, invasiveness, and mitochondrial activity of cancer cells.^1160^

Two recent papers^1162^^,^^1163^ are challenging this view. Xing et al^1163^ claim that TRPML1 activation inhibits autophagy (similar works^1147^^,^^1164^ discussed above) and that this autophagy inhibition suppresses cancer (melanoma) metastasis. Similarly, Du et al^1162^ suggest that TRPML1 small molecule activation induces Zn^2+^ release mediated cell death in metastatic melanoma, emphasizing that instead of inhibition, activation of TRPML1 may be beneficial in treating metastasis formation in cancer, at least in melanoma.

Due to the neurodegenerative phenotype in MLIV disease and several studies showing TRPML1 activation to rescue lysosomal storage and neurodegenerative disease phenotypes, TRPML1 appears to be a promising novel drug target for the treatment of such diseases. We will, therefore, in the following chapter, focus on this topic and discuss it in more detail after a brief discussion of currently available pharmacological tools to modulate TRPMLs.

### E Pharmacology of TRPMLs

**Fig. 8:**
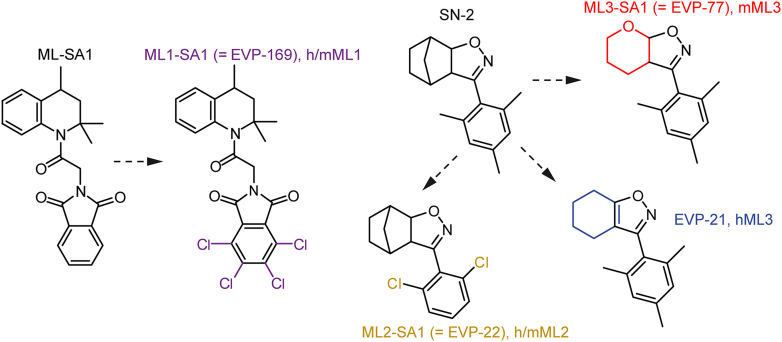
Shown are the chemical structures of ML-SA1 and SN-2 and their derivatives ML1-SA1 (EVP-169; isoform selective agonist for mouse/human TRPML1), ML2-SA1 (EVP-22; isoform selective agonist for mouse/human TRPML2), ML3-SA1 (EVP-77; isoform selective agonist for mouse TRPML3), and EVP-21 (isoform selective agonist for human TRPML3).

It was already mentioned that the currently known endogenous activators of TRPMLs are the phosphoinositides PI(3,5)P_2_ (agonist for all 3 TRPMLs) and PI3P (agonist^1076^ for TRPML3), while PIP_2_ inhibits TRPMLs (Table 9). How about lipophilic small molecule modulators of TRPMLs? In addition to Table 9 presented here, a comprehensive and detailed overview of the currently available pharmacology for TRPMLs has been published recently by Rautenberg et al^1092^ Of note, isoform-selective activators for all 3 TRPMLs have become available in recently: ML1-SA1 (EVP-169)^1088^ for human/mouse TRPML1, ML2-SA1 (EVP-22)^1082^ for human/mouse TRPML2, EVP-21 for human TRPML3, and ML3-SA1 (EVP-77)^1088^^,^^1165^ for mouse TRPML3. ML1-SA1 is structurally related to ML-SA1 published previously^1066^ (Fig. 8). ML2-SA1 is a derivative of the previously published^1087^ structure SN-2; likewise, ML3-SA1 (EVP-77) and EVP-21 are derived from SN-2 (Fig. 8). In contrast to these isoform-selective agonists, ML-SA1 and MK6-83 are not isoform-selective TRPML channel agonists.^1067^^,^^1068^

Regarding inhibitors of TRPMLs, there are currently only isoform-selective antagonists available for TRPML1: EDME (17*β*-estradiol methyl ether)^1095^ and its derivatives PRU-10 and PRU-12, the latter 2 showing reduced efficacy at the estrogen receptor *α* compared with EDME.^1095^ Other available inhibitors^1166^ are ML-SI1 and ML-SI3. From the original publication, it remains, however, unclear which of the stereoisomers of ML-SI1 and ML-SI3 are functionally active. ML-SI1 has 4 different stereoisomers, and currently, only racemic mixtures are commercially available.^1093^ One commercial product sold as a TRPML1 inhibitor is GW405833. This compound differs from the published structure^1166^ as it is not based on an indoline moiety (for details see Rautenberg et al^1092^). Importantly,^1093^^,^^1165^ this compound is inactive on TRPML1. Nevertheless, GW405833 was used at least in 2 publications erroneously as a TRPML1 inhibitor.^1123^^,^^1167^ In addition, GW405833 is also sold as a selective cannabinoid CB2 receptor agonist. Of note, ML-SI1 in the structure as published by Wang et al,^1166^ blocks both TRPML1 and TRPML2 with equal potency.^1093^^,^^1165^

Commercially available ML-SI3 is a racemic mixture of trans-enantiomers.^1093^ Separation of the trans-enantiomers of ML-SI3 revealed that the (–)-enantiomer is an inhibitor of all 3 TRPMLs, while the (+)-enantiomer inhibits TRPML1, but activates TRPML2 and TRPML3 (Table 9).^1093^ The commercially available racemic mixture also activates TRPML2 but blocks TRPML1 and TRPML3.^1093^

### F TRPMLs as therapeutic targets

That loss of TRPML1 leads to severe neurodegeneration (MLIV) is undisputable. But does activation of TRPML1 ameliorate lysosomal storage and neurodegenerative disease phenotypes? In 2012, Shen et al^1066^ claimed that abnormal lipid accumulation (cholesterol, sphingolipids, sphingomyelin) in Niemann-Pick type C1 patient cells can be reversed by TRPML1 activation. It was also shown^1066^^,^^1135^ that sphingomyelin can directly block TRPML1. FIG4 (encoding Sac3 protein) deficiency, which causes a rare peripheral neuropathy with severe motor deficits called Charcot-Marie-Tooth type 4J, results in decreased levels of both PI5P and the endogenous agonist of TRPML1, PI(3,5)P_2_. Zou et al^1168^ showed that TRPML1 activation rescues the abnormal lysosomal storage in FIG4-deficient cells and in ex vivo nervous tissue. Rescue effects of TRPML1 activation were recently confirmed for 2 subtypes of demyelinating Charcot-Marie-Tooth disease in an independent study.^1169^ Amelioration of lysosomal storage in Niemann-Pick type A and Fabry disease, as well as Niemann-Pick type C1 disease, was shown to depend on the BK that forms a physical and functional coupling with TRPML1. Importantly, Ca^2+^ release via TRPML1 activates BK, which in turn facilitates further Ca^2+^ release, enhancing membrane trafficking and lysosomal exocytosis.^1170^^,^^1171^

Tsunemi et al^1122^ found that increased lysosomal exocytosis by TRPML1 activation protects human inducible pluripotent stem cell (iPSC)-derived dopaminergic neurons in a model of familial PD from *α*−synuclein toxicity, caused by mutations in ATP13A2 (CLN12). Another link between PD and TRPML1 has recently been established by Sasazawa et al^1172^ reporting that acrolein, an aldehyde that is significantly elevated in PD patient serum, enhances autophagy via a newly discovered JIP4-TRPML1-ALG2 pathway.

In APP/PS1 double transgenic mice and hippocampal neurons with AD-like alterations, Zhang et al^1173^ found that overexpression of TRPML1 played a neuroprotective role in AD by ameliorating cognitive function and attenuating cognitive impairments. Xu et al.^1123^ reported that TFEB regulates, in a TRPML1-dependent manner, the lysosomal exocytosis of tau and that TFEB loss exacerbates tau pathology and spreading. In another recent study, Somogyi et al^1174^ show that dysfunction of TRPML1 is associated with abnormalities in the endolysosomal system in AD and APOE-ε4 iPSC-derived neurons. Inhibition of PIKfyve, the key enzyme in the production of the TRPML1 agonist PI(3,5)P_2_, recapitulated these results, while effects could be reverted or reduced by the TRPML agonist ML-SA1.

Other examples for TRPML1 activation providing potential therapeutic benefit in neurodegenerative or lysosomal storage diseases are HIV gp120-related lysosomal storage, where TRPML1 activation cleared amyloid β (Aβ) from lysosomal compartments in neurons^1175^^,^ and data suggesting TRPML1 activation to promote autophagy, facilitating the clearance of accumulated *α*-synuclein in both in vitro and in vivo models of MPP+/MPTP-induced Parkinson's disease.^1176^

In contrast to TRPML1, TRPML2 expression is largely absent from the human brain, while TRPML3 appears to be expressed to some extent in the hippocampus, cerebral cortex, and hypothalamus.^1080^ Importantly, TRPML3 activity increases with increasing pH, suggesting that even under conditions of increased lysosomal pH, as often observed in lysosomal storage and neurodegenerative diseases, TRPML3 would still be active to drive lysosomal exocytosis similar to TPC2, the activity of which does likewise not depend on the luminal pH.^1106^^,^^1117^^,^^1135^

Currently, it is unclear how much lysosomal Ca^2+^ release would be beneficial and how it can be finely controlled so that it is, on the one hand, sufficient enough to promote lysosomal exocytosis and autophagy, while on the other hand avoiding potential cytotoxicity due to cytosolic Ca^2+^ overload. The possibility of a hyperactive TRPML1 under certain disease conditions^1177^^,^^1178^ and the controversies around this possibility have been discussed recently.^1080^^,^^1135^ In addition, Zn^2+^, Fe^2+^, and other heavy metal ions, which may be released alongside Ca^2+^ from lysosomes after TRPML1 activation, may pose a risk for increased cytotoxicity.^1179^

In sum, despite compelling and increasing evidence for a beneficial effect of TRPML1 activation in clearing lysosomal storage and promoting lysosomal exocytosis and autophagy, more empirical evidence is needed, as well as safety and chronic dosing studies. It also remains unclear if a defect in lysosomal acidification can be corrected by TRPML1 activation, although some evidence suggests this.^1139^^,^^1175^

## VII TRPP channels

### A Introduction

TRP polycystin (TRPP) channels are Ca^2+^-permeable nonselective cation channels with conserved roles in biological processes such as tubular morphogenesis and left-right patterning of organs in vertebrates.^1180^^,^^1181^ TRPP channels are regarded as the most ancient subfamily of TRP channels, with orthologs in organisms ranging from yeast to mammals.^6^^,^^1182^ The founding member of the TRPP channels, TRPP2, was discovered as the *PKD2* gene product mutated in ADPKD.^1183^ TRPP channels form homotetrameric complexes and heterotetrameric protein assemblies with other TRP channels and polycystin-1 (PC1) family members. The physiological importance of the heteromeric PC1-TRPP2 receptor-channel complex is underscored by the fact that mutations in the *PKD1* gene, encoding PC1, also cause polycystic kidney disease.^1184^ Since the discovery of the genes encoding the founding members of the polycystins, *PKD1* and *PKD2*, 6 additional family members have been identified1185., 1186., 1187., 1188., 1189., 1190. based on sequence and structural homology: *PKD1L1*, *PKD1L2*, *PKD1L3*, *PKDREJ*, *PKD2L1*, and *PKD2L2*. The *PKD2*-like genes encode TRPP channels, whereas the *PKD1*-like genes encode PC1 family proteins that assemble with TRPP channels in a modular fashion. TRPP channels are found in various tissues and regulate calcium signaling in primary cilia and other cellular compartments. Recent advances in determining the structure and function of TRPP channels in homo- and heterotetrameric complexes have provided first insights into the structural basis for channel gating and ion permeation. Pharmacological modulators of TRPP channels are still scarce.

### B Domain topology, assembly, and functional characteristics of individual TRPP channels

The nomenclature of TRPP ion channels is ambiguous. Initial publications named the gene products of *PKD1* and *PKD2* PC1 and polycystin-2. The founding member of the TRPP subfamily, polycystin-2 (PC2), was later classified as TRPP2, and this designation is commonly used in the literature. The latter designation of TRPP2 as TRPP1 has not been widely adopted and has caused confusion, as it has been used for both PC1 and polycystin-2. We therefore advocate for the following TRP nomenclature: 1) only *bona fide* ion channels with 6 TMDs should be named TRPP channels, and 2) PC1-like proteins with 11 TMDs should be referred to with their gene names until a unified protein nomenclature exists (Table 1). Because of ambiguity, TRPP1 and TRPP4 should not be used.

#### 1 Domain topology and assembly

**Fig. 9:**
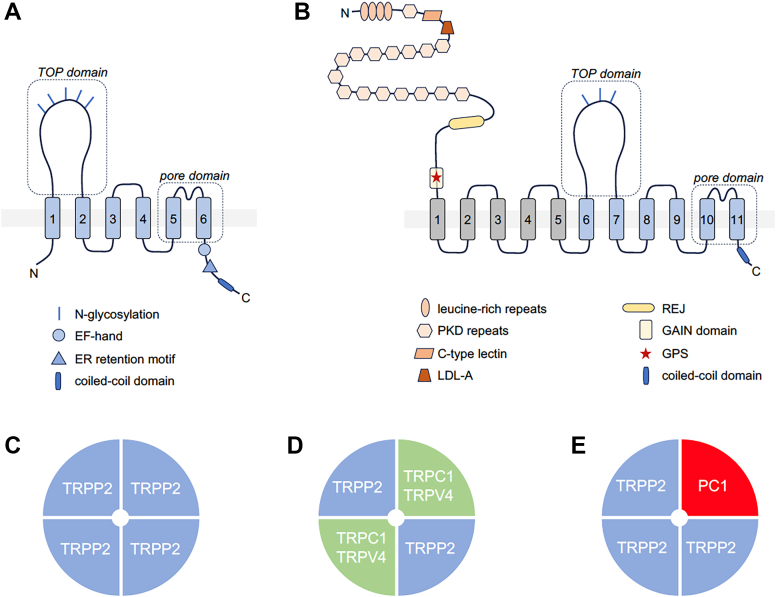
Domain topology and multimeric assemblies of TRPP channels. (A) Domain topology of TRPP2 comprising 6 TMDs (S1–S6). S1–S4 form the voltage sensor domain, the large extracellular loop between S1 and S2 forms the TOP domain, and S5–S6 form the pore domain. TRPP3 and TRPP5 have a similar overall topology with variable C-terminal regulatory motifs. (B) Domain topology of PC1 comprises 11 TMDs (S1–S11) with a large extracellular N-terminal domain. S6–S11 are highly homologous to TRPP2 and contribute to the pore domain in heteromeric PC1-TRPP2 complexes. PKD1L1, PKD1L2, PKD1L3, and PKDREJ have a similar topology with variable N-terminal extracellular domains. (C) TRPP2 and other TRPP channels form homotetrameric complexes, and (D) heteromeric complexes with other TRP channels, including TRPC1 and TRPV4. (E) TRPP2 and PC1 form heteromeric complexes with a 3:1 stoichiometry. The same subunit stoichiometry was shown for PKD1L3-TRPP3 channel complexes. GAIN, GPCR autoproteolysis-inducing; GPS, GPCR proteolytic site; LDL-A, low-density lipoprotein A; REJ, receptor for egg jelly.

Like all TRP channels, TRPP family members have 6 TMDs (S1–S6) and intracellular amino- and carboxy-termini (Fig. 9A). The TM segments S1–S4 form a voltage-sensor domain and the segments S5-S6 constitute the pore domain. A characteristic feature of TRPP ion channels is the large extracellular loop between S1 and S2, consisting of more than 200 amino acids in TRPP2, TRPP3, and TRPP5, respectively. This extracellular loop of TRPP2 contains 5 conserved asparagine-linked glycosylation sites (N299, N305, N328, N362, and N375), which are required for efficient TRPP2 biogenesis and stability.^1191^ The carboxy-terminal region of TRPP2 comprises motifs involved in channel regulation, assembly, and trafficking, including a Ca^2+^-binding EF hand, a CC domain, and an ER retention motif with an acidic amino acid cluster (Fig. 9A).1192., 1193., 1194., 1195., 1196., 1197., 1198., 1199. The EF hand has been implicated in Ca^2+^-dependent regulation of TRPP2.^1198^^,^^1199^ A more recent study, however, questions the hypothesis that Ca^2+^ occupancy of the TRPP2 EF hand is responsible for the regulation of channel activity.^1200^ The acidic cluster is involved in protein trafficking, whereas the 2 CC domains contribute to homo- and heteromerization of TRPP2 subunits.1193., 1194., 1195.^,^^1197^^,^^1199^

TRPP2 contains several predicted and validated phosphorylation sites, some of which have been studied in more detail (S76, S801, S812, and S829).^1192^^,^^1197^^,^1201., 1202., 1203., 1204., 1205., 1206., 1207. Glycogen synthase kinase 3 (GSK3) was shown to phosphorylate serine 76 to promote redistribution of TRPP2 from the plasma membrane to intracellular compartments.^1204^ Phosphorylation of serine 801 is increased by epidermal growth factor stimulation and appears to be protein kinase D-dependent.^1205^ Phosphorylation of serine 812 by PKC K2 contributes to regulation of TRPP2 trafficking and ion channel activity.^1197^^,^^1201^^,^^1207^ Serine 829 phosphorylation by aurora A or protein kinase A has been reported to modulate ion channel function.^1203^^,^^1206^ It should be noted, however, that the functional importance of these TRPP2 modifications has been difficult to evaluate as overexpressed TRPP2 is trapped in the ER, impeding the electrophysiological analysis at the plasma membrane (see below). The overall domain topology of TRPP3 and TRPP5 resembles TRPP2. While motifs such as C-terminal CC domains and phosphorylation sites have also been predicted in these channels, there is still much less information compared with TRPP2.^1208^^,^^1209^ TRPP3 channel function has been shown to be regulated by palmitoylation and phosphorylation at the cytoplasmic N-terminal domain (at cysteine 39 and threonine 39, respectively).^1210^ Furthermore,^1211^ TRPP3 was found to be regulated by cAMP signaling via a cluster of phosphorylation sites at S682, S685, and S686.

#### 2 Homotetrameric TRPP channel complexes

Recently, the first 3D structures of TRPP2 and TRPP3 have been resolved using single-particle cryo-EM.1212., 1213., 1214., 1215., 1216. These studies show that TRPP channels are assembled as homotetrameric complexes with each subunit comprising a voltage-sensor domain (S1–S4), a tetragonal opening for polycystins (TOP) domain, formed by the extracellular loop between S1 and S2, and a pore domain (S5–S6), jointly formed by the 4 subunits. The individual channel subunits interface through their TOP and pore domains, suggesting a role of these subunit interactions in homotetrameric assembly. The structural arrangement of the voltage-sensor and pore domains in TRPP2 and TRPP3 provides mechanistic insights into voltage-dependent gating.

The voltage-sensor domains of the respective TRPP subunits connect to the pore domains of neighboring subunits via an S4-S5 linker helix. This S4–S5 linker helix is thought to communicate the activation state of the voltage-sensor domain to control gating of the pore domain. The S4 segment of TRPP2 and TRPP3 contains 2 positive gating charges, which are thought to move outward in response to membrane depolarization.^1213^^,^^1217^ This outward movement may be coupled to the opening of the pore via lateral displacement.

The TOP domain extends from the S1 and S2 helices on the extracellular side of the voltage-sensor domain. This domain is not found in TRPCs, but is similar to a corresponding domain of TRPMLs.^1055^^,^^1060^ The TOP domain is composed of 5 *β* strands and 2 *α*-helices and forms extracellular contacts with the extracellular loop between S3 and S4, suggesting a functional connection to the voltage-sensor domain. In support of this notion, ADPKD-causing missense mutations in this domain can significantly shift the voltage dependence of TRPP2 opening.^1218^

The pore domain constitutes the ion-conductive pathway and the selectivity filter. TRPP2 and TRPP3 are Ca^2+^-permeable nonselective cation channels. TRPP3 is more selective for Ca^2+^ than TRPP2, probably because the selectivity filter of TRPP3 harbors a second ring of negatively charged aspartate residues that is not present in TRPP2.^1219^ Structural and functional studies suggest that TRPP channels have multiple gates, with the lower gate in the S6 segment being mobilized by uncoiling its secondary helical structure.^1214^ The upper gate is thought to be within the selectivity filter and might be involved in channel inactivation.1214., 1215., 1216. Asparagine 533 in the outer pore loop of mouse TRPP3 was shown to be essential for its voltage-dependent inactivation.^1220^

The carboxy-terminal domains have not been structurally resolved in the reported TRPP2 and TRPP3 structures. However, the structures of isolated fragments containing the EF hand or the CC domain have been determined.^1199^^,^1221., 1222., 1223. The isolated CC domain of TRPP2 forms trimers, which appears to contradict the homotetrameric assembly of whole TRPP channels.^1224^ Yet, the trimeric assembly of the isolated CC domains may be explained by the fact that heteromultimeric assemblies of TRPP channels with PC1 family proteins occur at a 3:1 stoichiometry (see below). While the precise role of the coiled-coil motif for TRPP channel assembly and structure is not known, it appears to be important for channel function, since truncating mutations that delete the CC motif of TRPP2 cause ADPKD (The ADPKD Mutation Database, https://pkdb.mayo.edu/variants).

#### 3 Heteromeric TRPP channel complexes

TRPP2 has been shown to interact with several TRP channels (TRPC1, TRPC3, TRPC4, TRPC5, TRPC7, and TRPV4) in heterologous expression systems.^1225^ There are currently no 3D structures of these heteromeric assemblies, but atomic force studies proposed a 2:2 stoichiometry with an alternating subunit arrangement for TRPP2/TRPC1 and TRPP2/TRPV4 heterotetramers, respectively.^57^^,^^128^^,^^384^^,^^1226^ It has been proposed that the channel properties of these heteromeric TRPP2 complexes are modulated by the TRP subunit composition, adapting the functional properties of TRPP2 to tissue-specific roles, including mechano- and thermosensation.^1226^ However, there is still limited information concerning the physiological role of most of these heteromeric TRPP2 complexes in vivo.

Heteromeric complexes formed by TRPP channels with members of the PC1 family have been shown to play an essential role in biological processes such as tubular morphogenesis and establishment of left-right asymmetry.^1180^ Mutations in the genes encoding PC1 and TRPP2 cause polycystic kidney disease in humans and model organisms (see “Expression pattern and primary physiological roles of TRPP channels” and “Human diseases associated with TRPP channels”). Both proteins interact to form a receptor-ion channel complex.^1227^^,^^1228^ PC1 and related family members (PKD1L1, PKD1L2, PKD1L3, and PKDREJ) are rather large proteins (210–520 kDa) with 11 TMDs (S1–S11). The 6 carboxy-terminal TMDs of PC1 (S6–S11) share high sequence homology with TRPP2. Despite this homology, PC1 is not an ion channel itself, but may contribute to the pore domain of heteromeric complexes with TRPP2. Members of the PC1 family have a large extracellular N-terminal domain, which is thought to be involved in the sensing of mechanical or chemical cues.1229., 1230., 1231., 1232. This domain contains multiple motifs suggesting interaction with cell matrix or extracellular proteins (Fig. 9B).^1233^^,^^1234^ PC1 has the largest extracellular domain with 3074 amino acids, followed by PKD1L1 (1784 amino acids), PKD1L2 (1344 amino acids), PKD1L3 (1083 amino acids), and PKD1REJ (1184 amino acids). The PC1 N-terminal domain contains multiple motifs, including leucine-rich repeats, 15 PKD repeats, an low-density lipoprotein A–related motif, a C-type lectin domain, and a receptor for egg jelly module (Fig. 9B). Interestingly, PC1 shows similarities to the adhesion class GPCRs (adhesion GPCRs). A common feature of PC1 and adhesion GPCRs is a GPCR autoproteolysis-inducing domain and autoproteolytic cleavage of the extracellular amino-terminus at a G protein-receptor-coupled proteolytic site.1235., 1236., 1237. Activation of adhesion GPCRs through a tethered agonist has been proposed to involve a stalk region preceding the first TMD.^1238^ Recent studies^1239^^,^^1240^ suggest that a similar mechanism may apply for the activation of PC1.

The 3D structures of the heteromeric PC1-TRPP2 complex and the PKD1L3-TRPP3 complex were determined using cryo-EM, revealing a 1:3 stoichiometry, which had already been proposed in earlier studies.1241., 1242., 1243.

Owing to the sequence homology to TRPP2, the S6–S11 TMDs of PC1 are arranged with similar symmetry to TRPP2 subunits within the heteromeric structure.^1212^^,^^1214^^,^^1216^^,^^1242^ The same holds true for the highly homologous TOP domains of PC1.^1242^ In PC1 and PKD1L3, the TOP domains extend from the extracellular S6–S7 loop (Fig. 9B). In contrast to homomeric TRPP2 and TRPP3 channels which have symmetric channel selectivity filters, the pore domain of heteromeric PC1-TRPP2 channels is asymmetric due to the contribution of the S10 and S11 segments of PC1-related subunits.^1241^^,^^1242^ Based on the structure of the pore domain, the cation selectivity of these heteromeric channels is predicted to be distinct from homomeric TRPP2 channels, because the PC1 pore loop lacks the aspartate residues found in TRPP2. This prediction is supported by electrophysiological experiments (see below). In the published structure of the PC1-TRPP2 complex, 3 positively charged residues in the pore lining S11 of PC1 (R4100, R4107, and H4111) plug the ion permeation pathway. It has been speculated that lateral displacement S11 of PC1, possibly coupled to conformational changes in distant parts of the complex, may gate the PC1-TRPP2 heteromeric channel.^1181^ Future studies of the structure of the heteromeric PC1-TRPP2 complex in the open state, ideally with a bound activating ligand, are required to unravel its gating mechanism.

The structure of the PKD1L3-TRPP3 complex was determined in a closed and in a Ca^2+^-bound open state.^1241^ Two Ca^2+^-binding sites that are probably involved in gating the channel complex were identified. In the closed state, the PKD1L3-TRPP3 complex is blocked by K2069 from PKD1L3, which appears to plug the ion permeation pathway in the absence of Ca^2+^. At high Ca^2+^ concentrations, K2069 of PKD1L3 is displaced by the Ca^2+^ ion coordinated by the D523 side chain of TRPP3 and main chain carbonyls of both TRPP3 and PKD1L3. The second Ca^2+^ binding site is in the extracellular cleft of the voltage sensor domain within the third TRPP3 subunit of the heteromeric complex. Electrophysiological experiments support the hypothesis that Ca^2+^ binding of the voltage sensor domain of TRPP3 is responsible for Ca^2+^-dependent activation.^1241^ In summary, the structures of the heteromeric TRPP channel complexes have provided mechanistic insights into ion permeation and gating. It should be noted that all structures of the heteromeric TRPP complexes have been determined using truncated forms of the PC1-related subunits. In the heteromeric PC1-TRPP2 structure, PC1 was missing the extracellular N-terminal domain and the intracellular C-terminus. In the PKD1L3-TRPP3 structure, PKD1L3 was missing its N-terminal extracellular domain and the first 5 TMDs (S1–S5).^1241^^,^^1242^

#### 4 Functional characteristics of individual TRPP channels

TRPP channels are Ca^2+^-permeable nonselective cation channels. Their biophysical properties are modulated by differential assemblies with members of the PC1 family. Here, we summarize the functional properties of individual homotetrameric and heteromeric TRPP channel complexes.

##### a TRPP2

TRPP2 function has been studied in the plasma membrane, in the ER, and in primary cilia. The functional analysis of TRPP2 in the plasma membrane has proven difficult because heterologously expressed TRPP2 in mammalian cell lines localizes mostly, if not exclusively, in the ER.^1192^^,^^1197^^,^^1198^^,^^1244^ Despite earlier studies reporting TRPP2 currents after heterologous expression in different cell types,^1245^ many later studies failed to record increased whole cell currents after overexpression of WT TRPP2 (with or without coexpression of PC1).^1197^^,^^1214^ Earlier functional studies of TRPP2 are reviewed elsewhere.^1245^ In the ER, TRPP2 operates as a Ca^2+^ release channel, and different mechanisms have been proposed on how this may affect Ca^2+^ signaling and ER Ca^2+^ homeostasis. One study showed that TRPP2-mediated Ca^2+^ release decreases the ER concentration, thereby regulating the sensitivity of cells to apoptotic stimuli.^1246^ Another study proposed that TRPP2 amplifies ER Ca^2+^ release via Ca^2+^-dependent activation of TRPP2,^1198^ whereas others reported increased Ca^2+^ release from the ER through direct association with the inositol trisphosphate receptor.^1247^ Reconstitution of TRPP2 proteins isolated from the ER was used to record single-channel currents and Ca^2+^ regulation of the channel.^1198^^,^^1207^

Recent progress in the electrophysiological characterization of TRPP2 channels has been achieved through 2 methodological breakthroughs: (1) direct electrophysiological recordings from primary cilia,^1248^^,^^1249^ and (2) GOF mutations in TRPP2 enabling the electrophysiological characterization in the plasma membrane.^1250^

TRPP2 localizes to the membrane of primary cilia.^1251^ Patch-clamp recordings from cilia showed that endogenous and heterologous TRPP2 channels have a cation permeability profile of K^+^ > Na^+^ >> Ca^2+^ with a single channel conductance of 139 pS (in the presence of K^+^). TRPP2 has a 10-fold higher permeability for Na^+^ than for Ca^2+^ ions.^1249^^,^^1252^^,^^1253^ Despite the relatively low Ca^2+^ selectivity, opening of TRPP2 channels can trigger Ca^2+^ signals in cilia and other cellular compartments due to the huge Ca^2+^ concentration gradient with an extracellular concentration that is 10,000 times higher than the intracellular Ca^2+^ concentration. Together with the negative membrane potential, this provides a big electrochemical driving force for Ca^2+^ to enter cells. TRPP2 is voltage-dependent with an outwardly rectifying current-voltage relationship. This voltage dependence is modulated through the intracellular Ca^2+^ concentration.^1200^^,^^1249^ Furthermore, TRPP2 whole cell cation currents at the plasma membrane could be recorded in *Xenopus* oocytes over-expressing a TRPP2 F604P GOF mutant, which has enabled functional studies of TRPP2 at the plasma membrane^1250^ and studies of disease-associated missense mutations in the pore loop of TRPP2 that alter its channel function.^1254^

In addition to homomeric complexes, TRPP2 forms heteromeric complexes with members of the PC1 family, which modulate its functional properties. The PC1-TRPP2 channel complex has been studied the most because of its involvement in ADPKD (see “Expression pattern and primary physiological roles of TRPP channels”). Despite intense research efforts, many functional features of this channel complex remain poorly understood. This can be explained by the fact that heterologous expression of PC1 together with TRPP2 does not give rise to constitutively active channels in the plasma membrane.^1253^^,^^1255^ Initial studies reporting increased whole cell currents upon co-expression of PC1 and TRPP2 in the plasma membrane^1196^^,^^1256^ could not be reproduced by others.^1253^^,^^1255^

Two recent studies have provided insights into the channel function of the PC1-TRPP2 complex. In the first study in *Xenopus* oocytes, co-expression of PC1 with TRPP2 harboring 2 GOF mutations (L677A/N681A) resulted in altered ion selectivity, with greater Ca^2+^ permeability compared with the TRPP2 mutant channels alone, suggesting a contribution of PC1 to the selectivity filter.^1257^ In a second study, the TRPP2 F604P GOF mutant was coexpressed with PC1 containing a strong N-terminal signal peptide to increase plasma membrane trafficking.^1255^ Kidney epithelial cells coexpressing these constructs showed constitutive outwardly rectifying ion currents, whereas coexpression of WT PC1 and TRPP2 produced no currents. Interestingly, the C-type lectin domain from the PC1 N-terminus was used as a soluble activator of the PC1-TRPP2 F604P complex, suggesting that extracellular ligands binding to the complex can modulate channel activity.^1255^ Furthermore, it was shown that cilia-enriched oxysterol 7*β*,27-dihydroxycholesterol is required for TRPP2 ion channel activation.^1258^ The key takeaway from these studies is that heteromeric PC1-TRPP2 channels without GOF mutations appear to be constitutively closed, and active mutant channels in the heteromeric complex are more Ca^2+^-permeable than homomeric TRPP2 channels. The identification of the physiological activation mechanism of the PC1-TRPP2 complex remains one of the most important future challenges, because it will enable the study of the biophysical properties of the native WT complex and downstream signaling pathways, which may be dysregulated in ADPKD.

The PKD1L1-TRPP2 complex is required for the establishment of left-right organ asymmetry (see “Expression pattern and primary physiological roles of TRPP channels”).1259., 1260., 1261. Cilia-mediated asymmetric Ca^2+^ signals in the embryonic node have been shown to result in asymmetric gene expression to establish left-right asymmetry.^1259^^,^^1260^^,^^1262^^,^^1263^ Genetic data from humans, mice, and zebrafish implicate the PKD1L1-TRPP2 complex in the generation of these asymmetric Ca^2+^ signals.1259., 1260., 1261.^,^^1264^ However, there are no direct measurements of PKD1L1-TRPP2 channels in the embryonic node to date. Future work will have to determine the biophysical properties of this complex and its activation mechanism.

Heteromeric TRPP channel complexes with PKDREJ have been proposed to play a role in fertilization. PKDREJ-TRPP2 and PKDREJ-TRPP3 co-immunoprecipitate when over-expressed in HEK293 cells.^1265^ To date, there are no functional channel data of these heteromeric complexes.

In summary, the modular assembly of TRPP2 with different members of the PC1 family appears to enable tissue-specific functions that are tuned to specific physiological requirements, eg, responsiveness to different, yet to be identified, ligands that activate the respective heteromeric complexes.

##### b TRPP3

Unlike TRPP2, ion currents from homomeric TRPP3 channels can be measured from the plasma membrane when heterologously expressed.^1208^ TRPP3 is an outwardly rectifying nonselective cation channel which conducts mono- and divalent cations.^1214^ TRPP3 is more Ca^2+^-selective than TRPP2 with a Ca^2+^ permeability that is 15 times higher than that for Na^+^, probably because of an additional aspartate (D525) in the selectivity filter.^1219^ TRPP3 has properties of voltage-dependent channels, such as voltage-dependent inactivation and tail currents after membrane repolarization.^1217^^,^^1220^ Similar to TRPP2, TRPP3 activity is modulated^1219^ by intracellular Ca^2+^. In heterologous expression systems (*Xenopus* oocytes and mammalian cells), TRPP3 has been shown to be activated by acidic and alkaline extracellular pH,^1266^^,^^1267^ and has been proposed to play a role in sour taste transduction (see “Expression pattern and primary physiological roles of TRPP channels”).

Heteromeric PKD1L1-TRPP3 channels have been shown to regulate the ciliary Ca^2+^ concentration.^1268^ Endogenous PKD1L1-TRPP3 channels have been measured directly by patch-clamping of primary cilia in fibroblasts and retinal pigment epithelial cells. These currents recorded from cilia were activated^1248^ by ATP and blocked by Gd^3+^. High membrane pressure increased the open probability of heteromeric PKD1L1-TRPP3 channels, but there is currently no data suggesting a direct role of this heteromeric complex in ciliary mechanotransduction. Since homomeric TRPP3 channels can be measured at the plasma membrane and in cilia, the contribution of the PKD1L1 subunit to the functional pore can be determined by comparing the permeation properties of the homomeric and heteromeric channels.^1214^^,^^1219^^,^^1248^ The single-channel conductance of homomeric TRPP3 channels is larger than the conductance of heteromeric PKD1L1-TRPP3 channels (156 pS versus 96 pS, respectively; with Na^+^ as charge carrier). The Ca^2+^ selectivity over Na^+^ of monomeric TRPP3 channels is higher than that of PKD1L1-TRPP3 channels (15- versus 6-fold, respectively). These alterations of the biophysical properties of the heteromeric complex are likely explained by the contribution of the PKD1L1 pore domain (S10-S11) to the selectivity filter of the heteromeric PKD1L1-TRPP3 complex. The precise structural features determining these biophysical properties remain to be investigated.

Co-expression of PKD1L3 and TRPP3 in HEK293 cells, *Xenopus* oocytes, and HEK cells results in ion currents that are activated by extracellular Ca^2+^ and pH changes (acidic and alkaline).^1269^^,^^1270^ PKD1L3-TRPP3 operates as nonselective cation channel with preference for Ca^2+^ over Na^+^ (P_Ca2+_/P_Na+_ ≈ 11). Interestingly, the pH- or Ca^2+^-activated currents have no voltage dependence.^1271^^,^^1272^ Since the regulation by Ca^2+^ and pH is also observed in homomeric TRPP3 channels, it is difficult to distinguish whether this regulation is a feature of homo- or heteromeric channels in an overexpression system. Taken together, PKD1L3 and TRPP3 form complexes, but their functional features and their physiological relevance require further investigation.

##### c TRPP5

The biophysical properties and the physiological function of TRPP5 channels are the least well characterized of the TRPP channels. It has been reported that overexpression of TRPP5 in HEK293 cells produces single-channel currents with a conductance of 25 pS that are not voltage-sensitive.^1265^^,^^1273^ There are no reports of endogenous TRPP5 currents.

### C Expression pattern and primary physiological roles of TRPP channels

#### 1 Expression pattern

The genes encoding TRPP channels are expressed in many organs in vertebrates. Transcriptome analyses have detected mRNA of TRPP channels in nearly all human and mouse tissues. Targeted studies focusing on individual TRPP channels have confirmed and expanded these findings: *PKD2* and *PKD2L1* transcripts are present in numerous fetal and adult tissues, including the heart, brain, lungs, spleen, testes, ovaries, and kidneys.^1183^^,^^1188^^,^^1189^^,^^1209^^,^^1272^^,^^1274^ *PKD2L2* expression appears to be more restricted to the brain and testis.^1185^^,^^1189^ Splice variants of TRPP channels have been identified, but their functional properties are currently unknown.^1183^^,^^1185^^,^^1188^^,^^1189^

*PKD2* expression is modulated by post-transcriptional regulation. The RNA-binding protein bicaudal C (BICC1) and the microRNA group 17 (*miR-17*) have been reported to regulate TRPP2 expression levels in opposite directions.1275., 1276., 1277. *miR-17* has been shown to repress *Pkd2* expression by binding on the 3'-untranslated region of *Pkd2* mRNA. This may be of physiological relevance since overexpression of *miR-17* in the kidneys of transgenic mice leads to kidney cysts. In contrast, loss of *miR-17* reduced cyst growth in a mouse model with polycystic kidney disease caused by *Kif3a* KO.^1275^ Conversely, BICC1 enhances *Pkd2* mRNA stability and translation efficiency.^1277^ Loss of *Bicc1* results in cystic kidneys in model organisms and antagonizes the repressive activity of *miR-17*.^1277^ Since *miR-17* targets many genes, including several genes associated with cystic kidney disease, it remains to be determined whether the effects of *miR-17* on kidney cysts are caused solely by the reduction of TRPP2 expression.

TRPP2 and PC1 have also been studied in invertebrate model organisms, which provided fundamental biological insights such as the discovery that the polycystins localize in primary cilia.1278., 1279., 1280., 1281., 1282., 1283. In *Caenorhabditis elegans* and *Drosophila melanogaster*, *Pkd2* expression is restricted to ciliated cells, namely male-specific sensory neurons and spermatozoa, respectively.^1278^^,^^1283^ In the meantime, a convergence of additional findings from mammalian model organisms suggests that defective ciliary signaling plays an important role in the pathogenesis of polycystic kidney disease and related disorders, which are now collectively called ciliopathies.^1284^ In addition to primary cilia, PC1 and TRPP2 have been found in the ER and the lateral membrane.^1244^^,^^1285^ More recently, fragments of PC1 have also been detected in mitochondria.1286., 1287., 1288.

The physiologically relevant cellular localization of the PC1-TRPP2 complex is debated, but considerable evidence suggests that this channel complex functions in primary cilia or in the plasma membrane. However, a function in other membranes, for example, in the ER, cannot be ruled out and requires further studies. Trafficking of PC1 and TRPP2 to the plasma membrane or cilia appears to be interdependent, supporting the hypothesis that the heteromeric complex, rather than homomeric assemblies of each subunit, is the functionally relevant channel complex at these locations.^1289^^,^^1290^ However, it has also been reported^1291^ that TRPP2 traffics to cilia without PC1. In addition to PC1, multiple other proteins have been shown to be involved in the trafficking of TRPP2 to cilia and the plasma membrane.^1197^^,^^1244^^,^^1292^^,^^1293^

The identification of specific cell types expressing TRPP proteins in vivo has been challenging. While TRPP2-specific antibodies enable detection of TRPP2 by Western blot in the kidney, in the heart, and in other organs, the unequivocal detection of the cellular and subcellular distribution of TRPP2 protein in vivo has been hampered by low expression levels and the lack of antibodies with sufficient sensitivity and specificity for immunohistochemical and immunofluorescence studies. The same applies to TRPP3 and TRPP5. In the future, this limitation may be overcome by the introduction of epitope tags to endogenously expressed TRPP channels,^1287^ or by generating TRPP-reporter alleles to detect cell types expressing these channels using combinatorial genetic approaches, which have been successfully employed to detect the expression of other TRP channels in specific cell types.^1294^

#### 2 Primary physiological roles of TRPP channels

The physiological roles of TRPP channels have been studied in several model organisms with a focus on TRPP2 function because of its relevance in human disease.

##### a TRPP2

**Fig. 10:**
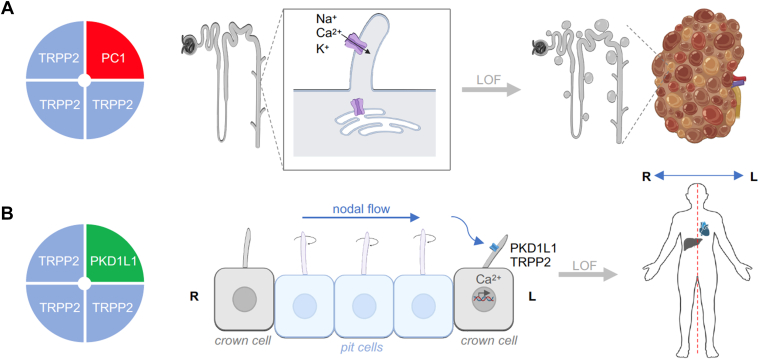
Physiological roles of heteromeric TRPP channels and pathologies caused by loss of function. (A) Left panel: heteromeric TRPP2-PC1 channels regulate the morphology of epithelial tubules in the kidney and other organs (eg, liver and pancreas). Middle panel: The channels operate in the primary cilium and ER and are thought to trigger Ca^2+^ signals. The activation mechanism of the TRPP2-PC1 complex and the downstream effectors controlling tubular morphology are unknown. Right panel: LOF of the TRPP2-PC1 complex results in focal cyst formation in the nephron, resulting in polycystic kidney disease. (B) Left panel: Heteromeric TRPP2-PKD1L1 complexes regulate left-right patterning during embryonic development. Middle panel: Left-right patterning in the embryonic node is driven by motile cilia in pit cells, creating a leftward nodal flow. Sensory cilia in perinodal crown cells detect flow-mediated mechanical or chemical signals via ciliary TRPP2-PKD1L1 channels. This triggers asymmetric Ca^2+^ signals in the embryonic node, resulting in asymmetric gene expression, which specifies left-right asymmetry. Right panel: LOF of the TRPP2-PKD1L1 complex causes left-right asymmetry defects.

The primary physiological roles of TRPP2 in vertebrates are the regulation of tubular morphogenesis and the establishment of organ left-right asymmetry.^1180^^,^^1295^ The importance of TRPP2 in controlling the morphology of epithelial tubules was first recognized when *PKD2* was cloned as the second gene mutated in ADPKD patients.^1183^ The requirement of TRPP2 for the formation of properly shaped tubules and for left-right patterning was later confirmed in mouse and zebrafish.1296., 1297., 1298., 1299. Loss of TRPP2 results in polycystic kidney disease in mice,^1299^ and pronephric cysts in zebrafish larvae.^1297^ Constitutive *Pkd2* KO mice develop cystic kidneys, edema, and hemorrhage and die *in utero* around midgestation.1298., 1299., 1300. Kidney cysts originate from dilatations along all nephron segments mimicking cyst formation in human ADPKD, with the notable exception that cyst formation in ADPKD is focal due to loss of heterozygosity in individual tubule cells, whereas PKD mouse models display much more widespread cyst formation due to *Pkd2* inactivation in all tubular epithelial cells. A mouse model with a *Pkd2* allele prone to spontaneous recombination (*Pkd2*^*WS25*^) mimics the loss of heterozygosity through somatic mutations in ADPKD and shows focal cyst formation similar to the human disease.^1299^ Conditional inactivation of *Pkd2* in specific cell types prevents embryonic lethality and enables studies of organ-specific functions of TRPP2.^1300^ Mutations in *PKD1* in humans or KO of *Pkd1* in mice also cause polycystic kidney disease.^1301^ Multiple lines of evidence, including the similarity of KO phenotypes of *Pkd1* and *Pkd2*, the coassembly of PC1 and TRPP2 in a heteromeric complex, and their interdependence of trafficking to cilia, support the notion that the PC1-TRPP2 complex rather than homomeric assemblies of these proteins are critical for the proper regulation of tubular shape (Fig. 10A). How Ca^2+^ signals triggered by this complex control the shape of epithelial tubes remains to be determined.

A second important physiological function of TRPP2 is its role in the establishment of left-right asymmetry. Loss of TRPP2 in mice and zebrafish causes left-right asymmetry defects that have been shown to be caused by impaired TRPP2-mediated Ca^2+^ signaling in the embryonic node.^1261^^,^1296., 1297., 1298. Left-right asymmetry is controlled by Ca^2+^-dependent asymmetric gene expression in cells on one side of the embryonic node, a transient concave structure in the midline of the postgastrulation embryo.^1302^ The cells in the embryonic node are ciliated. So-called pit cells in the embryonic node generate fluid flow directed toward the left side by the beating of motile cilia. Perinodal crown cells possess immotile primary cilia, which are required to sense the fluid flow to trigger intracellular Ca^2+^ signals (Fig. 10B).^1261^^,^^1302^ It is still debated whether the signal or stimulus sensed by these cilia is mechanical or chemical.^1261^^,^^1262^^,^1296., 1297., 1298.^,^^1303^ Notably, *Pkd1* KO mice do not display left-right asymmetry defects despite otherwise extensive phenotypic similarities to *Pkd2* KO mice. However, loss of PKD1L1 causes left-right asymmetry defects in humans and model organisms.^1259^^,^^1260^^,^^1264^ Since Ca^2+^ transients in perinodal crown cells require both TRPP2 and PKD1L1, and both channel subunits localize to primary cilia, PKD1L1-TRPP2 heteromeric channels probably function as sensors of nodal flow in the embryo (Fig. 10B).

TRPP2 channels are broadly expressed and likely have additional physiological functions. This is supported by the embryonic lethality of constitutive *Pkd2* KO mice, which is not caused by the kidney phenotype.^1299^^,^^1300^ Instead, the embryonic lethality in *Pkd2* KO mice appears to be caused by vascular defects in the placenta.^1300^ TRPP2 and PC1 appear to have a role in vascular integrity, since loss of function of both proteins causes cardiovascular phenotypes, ranging from cardiac valve defects to aneurysms and abnormal vascular permeability in KO mice.^1300^^,^^1304^^,^^1305^ Extrarenal manifestations of ADPKD patients support a role of PC1 and TRPP2 in the cardiovascular system.^1306^ In cardiomyocytes, TRPP2 has been reported to regulate Ca^2+^ release through ryanodine receptors through direct association.^1307^ A recent study showed that natriuretic peptide production requires TRPP2 in the heart, and loss of this pathway may contribute to the development of hypertension in ADPKD.^1308^ Studies of TRPP2 in the regulation of vascular tone are contradictory. In arterial smooth muscle cells, TRPP2 is proposed to contribute to systemic blood pressure and to the myogenic response in cerebral arteries through vasoconstriction.^1309^^,^^1310^ In vascular endothelial cells, TRPP2 was reported to mediate vasodilation through activation of nitric oxide synthase.^1311^ In addition, TRPP2 and Filamin-A have been proposed to regulate pressure sensing in mouse vascular smooth muscle cells, by fine-tuning stretch-activated channels to adapt the vascular myogenic response.^1312^ These seemingly paradoxical functions of TRPP2 in the vasculature might be explained by differences in specific locations and cell types within the vasculature. Further studies are required for a comprehensive understanding of TRPP2 function in the vasculature and other organs.

##### b TRPP3

The physiological functions of TRPP3 are much less well understood. The phenotypes of TRPP3 (*Pkd2l1*) KO models suggest functions in the CNS, cardiomyocytes, and early development. *Pkd2l1* KO mice show hippocampal and thalamo-cortical hyperexcitability with increased susceptibility to seizures.^1313^ Like TRPP2, TRPP3 localizes to primary cilia. TRPP3 channel activity has been measured in neurons contacting the subependymal cerebrospinal fluid. These neurons have protrusions with a primary cilium that extends into the central canal, where it is thought to sense mechanical or chemical signals from the cerebrospinal fluid.^1274^^,^^1314^^,^^1315^ In zebrafish, related neurons contacting the cerebrospinal fluid were shown to be mechanosensitive cells. The detection of cerebrospinal fluid flow through these neurons was shown to require mechanosensitive TRPP3 channels.^1315^

The organismal function of heteromeric PKD1L1-TRPP3 channels remains poorly understood. PKD1L1-TRPP3 channels have been reported^1268^ to control the Ca^2+^ concentration in cilia and to regulate Hedgehog-dependent transcription of glioma-associated oncogene homolog 1. The physiological consequences of these cellular events in vivo remain to be determined.

Heteromeric PKD1L3-TRPP3 channels have been proposed as a candidate sour taste receptor in gustatory cells.^1272^^,^^1316^ TRPP3 is expressed in some gustatory type III cells, and acid-evoked Ca^2+^ responses and optogenetic activation of these cells support a role of these cells in sour taste perception.^1270^^,^^1316^^,^^1317^ However, the role of the PKD1L3-TRPP3 in sour taste transduction is controversial. Mice with genetic ablation of TRPP3-expressing cells were shown to be completely devoid of acid responses in electrophysiology recordings to sour stimuli, supporting a role of these cells in sour taste reception.^1316^ Based on these and additional results showing acid activation of the complex, PKD1L3-TRPP3 channels were proposed to form the sour taste receptor.^1270^ Subsequent studies in PKD1L3-deficient mice, however, showed normal sour taste responsiveness in behavioral and electrophysiological experiments.^1318^

##### c TRPP5

Mouse TRPP5 mRNA and protein expression have been reported in spermatocytes and spermatids, but its role in male reproduction or other physiological functions has not been studied yet.^1319^

### D Human diseases associated with TRPP channels

#### 1 TRPP2

TRPP2 was first identified as the gene product of *PKD2*, the second causative gene for ADPKD.^1183^ Mutations in *PKD2* account for ∼15% of ADPKD cases, mutations in *PKD1* for ∼80%, and a few additional genes for the remaining 5%.^1184^^,^^1320^ ADPKD is by far the most common genetic cause of kidney failure and affects ∼1/1000 individuals in the general population.^1320^^,^^1321^ The disease is characterized by polycystic kidneys, with cyst development starting in the fetus and continuing through a patient’s lifetime. Continuous development and growth of cysts compresses the remaining tubules. In the majority of patients, this results in reduced kidney function and ultimately kidney failure. The clinical course of ADPKD is highly variable, but ∼50% of patients have kidney failure by 60 years of age.^1321^ Multiple extra-renal clinical manifestations, including liver cysts, pancreas cysts, intracranial aneurysms, and cardiac valvular disease, show that ADPKD is a systemic disorder.^1320^^,^^1321^ These extrarenal clinical manifestations point to functions of TRPP2 and PC1 in multiple organs, which are continuing to be studied in conditional mouse models.

Hundreds of unique ADPKD mutations have been identified, which are spread across *PKD1* and *PKD2* without obvious mutational hotspots (The ADPKD Mutation Database, https://pkdb.mayo.edu/variants). Patients with *PKD1* mutations tend to have more severe disease compared with those with *PKD2* mutations, and truncating mutations usually result in a more severe phenotype than nontruncating missense mutations.^1322^ There is significant inter- and intrafamilial variability in ADPKD symptoms even in patients with the same germline mutation. This suggests the existence of genetic, environmental, and epigenetic modifiers of ADPKD.

Each human kidney has about 1,000,000 nephrons. However, cysts develop only in a very small fraction (1%–5%) of nephrons. The focal nature of cyst formation in ADPKD can be explained by the 2-hit model.^1323^^,^^1324^ According to this model, a germline mutation (first hit) and a somatic mutation (second hit) in the normal allele are required for cyst formation in ADPKD. The loss of heterozygosity in kidney cells leads to the complete loss of functional polycystin proteins, which causes focal cyst formation. Thus, even though the mode of inheritance of ADPKD is dominant, the process of cyst formation is recessive at the cellular level. Multiple lines of evidence ranging from genetic analyses of cyst epithelia in patients to mouse models support the 2-hit model.^1299^^,^^1324^^,^^1325^

#### 2 TRPP3

To date, no variants in *PKD2L1*, the gene encoding TRPP3, have been associated with human disease.

#### 3 TRPP5

Like for TRPP3, there are no reports of human disease associated with variants in TRPP5.

### E Pharmacological modulators of TRPP channels

There is very limited information on pharmacological modulators of TRPP channels. No validated specific activators or blockers of TRPP2 are available because of the difficulty of measuring TRPP2 channel activity in heterologous expression systems. A recent study showed that some TRPML agonists (MK6-83, ML2-SA1, SF-21, SF-22, SF-23, SF-24, SF-31, SF-32, SF-33, SF-41, SF-71, SN-2, and rapamycin) inhibit the activity of TRPP2 with a F604P GOF mutation at high concentrations (see “Pharmacological modulators of TRPP channels”). Two of these TRPML agonists, ML-SA1 and SF-51, further activate the TRPP2 F604P channel, but not WT TRPP2, at low concentrations and inactivate it at higher concentrations.^1326^ TRPP3 is activated by acidic pH (see above), and is blocked by flufenamic acid at rather high concentrations (0.5 mM).^1208^ Furthermore, TRPP3 has been shown to be inhibited by amiloride, phenamil, benzamil, and 5-(*N*-ethyl-*N*-isopropyl)amiloride with an order of potency of phenamil > benzamil > 5-(*N*-ethyl-*N*-isopropyl)amiloride > amiloride, with IC_50_ values of 0.14, 1.1, 10.5, and 143 *μ*M, respectively.^1327^ There is still only one study reporting TRPP5 channel measurements,^1273^ and no pharmacological modulators of TRPP5 are available to date.

### F Ongoing or completed clinical trials with TRPP channels as therapeutic targets

There are no ongoing or completed clinical trials with TRPP channels as therapeutic targets. The majority of ADPKD is caused by mutations in *PKD1,* which led to the hypothesis that pharmacological activation of TRPP2 might mitigate the disease. However, there are no validated pharmacological activators of WT TRPP2 to date, and it remains to be determined whether pharmacological activation of TRPP2 can compensate for the loss of PC1, which is thought to be an essential subunit of the heteromeric PC1-TRPP2 complex. The development of ivacaftor and related drugs for the treatment of cystic fibrosis has shown the efficacy of potentiators and correctors of mutated ion channels harboring missense mutations.^1328^ It is conceivable that similar approaches might be applicable for ADPKD.

## VIII Conclusions and outlook

Recent experiments using animal disease models and human genetic studies have linked TRP channels to various pathophysiological processes, highlighting their broad therapeutic potential. Moreover, significant progress has been made in developing potent pharmacological agents targeting TRP channels in conjunction with electrophysiological and structural analysis of these proteins, which provides the mechanistic basis for innovative treatments of a wide array of human disorders. Despite the relevance of TRP channels as pivotal therapeutic targets for the treatment of human diseases, the clinical modulation of TRP channels has turned out to be more challenging than initially anticipated. The following 3 preclinical issues deserve future attention. (1) There still is a pressing need to further our understanding of the pathophysiological role of TRP channels, their exact contribution to cellular, tissue, and organismal homeostasis and dysfunction, including activation mechanisms in a native environment and reliable tissue expression with high spatial resolution. (2) Unwanted side effects, as noted in clinical trials, may arise from either off-target or off-tissue interactions of drug candidates. Recent advances in molecular approaches, such as single-particle cryo-EM, in combination with AI-guided computational methods, will refine the development of modality-specific and activity-dependent modulators, which can be validated through in-depth biophysical analyses. To limit off-tissue side effects, the direct local or topical application of TRP channel modulators appears to be an appropriate strategy. Long-term toxicity of topically applied chemical probes and drug candidates can be averted by controlled local inactivation of compounds, for instance, by introducing photoswitches. (3) To foster clinical translation, reliable and robust preclinical disease models must be developed, including genetically modified mouse models, in vitro human-derived organoids, and engineered human tissue cultured in biomimetic chambers. In this regard, progress in cellular reprogramming of iPSCs holds the promise of providing relevant preclinical models for early validation of TRP channel modulators.

## Conflict of interest

Thomas Gudermann functions as an Associate Editor of Pharmacological Reviews. All other authors declare no conflicts of interest.
